# Therapeutic potential of tranilast for the treatment of chronic graft-versus-host disease in mice

**DOI:** 10.1371/journal.pone.0203742

**Published:** 2018-10-11

**Authors:** Shin Mukai, Yoko Ogawa, Hideyuki Saya, Yutaka Kawakami, Kazuo Tsubota

**Affiliations:** 1 Deaprtment of Ophthalmology, Keio University School of Medicine, Tokyo, Japan; 2 Division of Cellular Signaling, Institute for Advanced Medical Research, Keio University, School of Medicine, Tokyo, Japan; 3 Division of Gene Regulation, Institute for Advanced Medical Research, Keio University, School of Medicine, Tokyo, Japan; University of Kentucky, UNITED STATES

## Abstract

Chronic graft-versus-host disease (cGVHD) is a marked complication of hematopoietic stem cell transplantation, and multiple organs can be affected by cGVHD-induced inflammation and fibrosis. In clinical settings, immunosuppressive agents have been the last resort to treat cGVHD. However, it has been only partially effective for cGVHD. Hence, efficacious treatment of cGVHD is eagerly awaited. Our previous work suggested that oxidative stress was elevated in cGVHD-disordered lacrimal glands and that epithelial-to-mesenchymal transition (EMT) was implicated in fibrosis caused by ocular cGVHD. In addition, our recent article demonstrated that thioredoxin interaction protein (TXNIP) and transcription factor nuclear factor kappa-light-chain-enhancer of activated B cells (NF-𝛋B) were associated with the development of cGVHD. After our search for effective drugs, we chose tranilast to combat systemic cGVHD. Tranilast is known to (1) act as an inhibitor of the inflammatory molecules TXNIP and NF-κB and (2) exert anti-fibrotic, anti-EMT and anti-oxidative effects. To investigate the effectiveness of tranilast for cGVHD, we used an MHC-compatible, multiple minor histocompatibility antigen-mismatched murine model of cGVHD. Tranilast or a solvent-vehicle were orally given to the allogeneic bone marrow transplantation (allo-BMT) recipients from the day before allo-BMT (Day-1) to Day 27 after allo-BMT. Their cGVHD-vulnerable organs were collected Day 28 after allo-BMT and analyzed by using various methods such as histology, immunohistochemistry and immunoblotting. As indicated by our results, tranilast alleviated cGVHD-elicited inflammation and fibrosis by suppressing the expression and/or activation of TXNIP and NF-κB and preventing EMT. Taken together, although this strategy may not be a complete cure for cGVHD, tranilast could be a promising medication to ameliorate cGVHD-triggered disabling symptoms.

## Introduction

Chronic graft-versus-host disease (cGVHD) is an extensive complication of allogeneic hematopoietic stem cell transplantation (allo-HSCT) and can impact patients’ quality of life in a detrimental manner. In many cases, cGVHD appears 6 months or later and its symptoms are similar to those of autoimmune diseases.[1, 2] Virtually all organs are vulnerable to cGVHD, and severe inflammation and fibrosis induced by cGVHD are highly problematic.[3, 4] For example, cGVHD patients can suffer from disabling symptoms such as dry eye, skin rashes, diarrhea and respiratory failure.[3, 4] Although there is a dearth of information about the exact mechanisms of cGVHD, minor histocompatibility differences between donor and recipient cells are thought to play a detrimental role in the establishment and development of cGVHD.[1] Substantial time and effort have been invested into the creation of effective remedies for cGVHD over the last decades, and several efficacious therapies for cGVHD in mice have recently been reported. [5–9] Nonetheless, there remains a considerable unfulfilled need in the treatment of cGVHD.

Thus, we were urged to devise sophisticated strategies to tackle cGVHD. As indicated by our earlier work, oxidative stress was augmented in the lacrimal glands disordered by cGVHD, and epithelial-to-mesenchymal transition (EMT) detrimentally contributed to fibrosis elicited by ocular cGVHD.[10, 11] Furthermore, our recent report suggested that thioredoxin interaction protein (TXNIP) and transcription factor nuclear factor kappa-light-chain-enhancer of activated B cells (NF-κB) were correlated with the pathogenic processes of cGVHD.[12] NF-κB is known to be detrimentally involved in the onset and progression of a variety of inflammatory diseases.[13–15] Prior to our study, Abedi and co-workers demonstrated that NF-κB is highly activated in organs disordered by cGVHD and that suppression of NF-κB could be an effective remedy for cGVHD.^9^ TXNIP is reported to activate the inflammasome nod-like receptor family, pyrin domain containing 3 (NLRP3) and whereby cause extensive inflammation.[16]

On the basis of these findings, we sought potentially effective drugs for cGVHD and encountered the commercially available compound tranilast (TL) (**S1 Fig**). TL is reported to (1) serve as an inhibitor of the inflammation-associated molecules TXNIP and NF-κB and (2) have anti-fibrotic, anti-EMT and anti-oxdative effects.[17–20] Clinical practice has been utilizing this chemical entity to treat asthma, atopic dermatitis, keloid and autoimmune disorders over the past decades.[21] This demonstrable fact underpins the safety of TL, and our previous results indicated that a topical application of TL could be efficacious for the treatment of ocular cGVHD.[22]

Hence, it was envisioned that systemic injection of TL could be a promising strategy to cure cGVHD-triggered systemic inflammation and fibrosis. In this article, we report our innovative utilization of the medically compelling compound TL for the treatment of systemic cGVHD.

## Methods

Eight-week-old B10.D2 were purchased from Sankyo Laboratory, Inc. (Tokyo, Japan). All the scientific experiments on mice were performed according to the Animal Welfare Act at Keio University School of Medicine. Our protocols for experiments on animals were approved by the animal care and use committee at Keio University (Approval Number: 09152).

### Bone marrow transplantation

Bone marrow transplantation (BMT) was conducted to experimentally induce cGVHD in mice.[23] In the case where the donors were B10.D2 mice and the recipients were BALB/c mice, it was allogeneic BMT (allo-BMTs) to produce a murine model of cGVHD. The recipients were irradiated with 700 cGy prior to the BMT, and the lethal irradiation was preformed using a Gammacel 137 Cs source (Hitachi Medico, Ltd, Tokyo, Japan). A suspension containing 1 x 10^6^ bone marrow cells and 2 x 10^6^ spleen cells from the donors (BM + SC) or 1 x 10^6^ bone marrow cells from the donors (BM only) was administered to each of the recipient mice via tail vein. The donor cells were suspended in RPMI 1640 (Life Technologies Japan Ltd, Tokyo, Japan).

### Treatment of allogeneic BMT recipient mice with Tranilast

We performed allo-BMT as stated above, and the BM + SC recipient mice were divided into 2 groups. Tranilast (TL) (Kissei Pharmaceutical. Co. Ltd., Tokyo, Japan) was suspended in a mixture of PBS and carboxymethylcellulose (CMC). One group was treated with tranilast (2 mg/kg) (Aldrich, St. Louis, MO), and the other was given the solvent-vehicle PBS-CMC by oral administration. This was also applicable to the BM-only recipient mice. We administered tranilast or the solvent-vehicle to the allogeneic BMT recipients twice per day from the day before BMT (Day -1) to Day 27 after BMT. They were euthanized by cervical dislocation Day 28 after BMT. In this study, the following cGVHD-susceptible organs were analyzed: extra-orbital lacrimal glands, the proximal part of small intestine, dorsum skin, liver, salivary glands, lung, large intestine and eyes.

### Histological analysis and immunohistochemistry

Four weeks after BMT, extra-orbital lacrimal glands, the proximal part of small intestine, dorsum skin, liver, salivary glands, lung, large intestine and eyes were collected from the transplant recipients. These samples were subsequently fixed with 10% neutral-buffered formalin and embedded in paraffin. The paraffin blocks were cut into 7μm-thick sections, and then stained with (1) hematoxylin and eosin, (2) Mallory’s trichrome[24, 25] and (3) antibodies used in this study. For immunohistochemical assays, paraffin was removed in the first instance, followed by the recovery of the antigens using either of the following 2 antigen retrieval methods. To stain the sections with a CD45 antibody (30-F11, BD Pharmingen, San Jose, CA) or an E-cadherin antibody (24e10, Cell Signaling Technology, Danvers, MA), they were immersed in the antigen retrieval solution (Target Retrieval Solution; Dako, Glostrup, Denmark) and then boiled with a microwave oven for 10 min. In the case of an HSP47 antibody (M16.10A1, Stress Gen Biotechnologies Corp, San Diego, CA), the sections were soaked in the antigen retrieval solution (Dako) and subsequently heated at 120°C with an autoclave for 20 min. After the antigen retrieval, the sections were blocked with 10% normal goat serum, and the reactions between the antigens in tissue sections and the primary antibodies were conducted at 4°C overnight. The sections were then treated with fluorophore-labelled secondary antibodies at RT for 45 minutes and mounted with an anti-fading mounting medium (Fluorescent Mounting Medium; Dako). Fluorescence images were taken with an LSM confocal microscope (Carl Zeiss, Jena, Germany). As for the counting of HSP47^+^ cells, five areas of each tissue section were randomly photographed under 400X magnification, and the number of HSP47^+^ cells in the individual images was subsequently determined.

The following secondary antibodies were used in this study: goat anti-mouse IgG (H+L) secondary antibody, Alexa Fluor 488 conjugate (Molecular Probes, Eugene, OR), goat anti-rat IgG (H+L) secondary antibody, Alexa Fluor 568 conjugate (Molecular Probes) and goat anti-rabbit IgG (H+L) secondary antibody, Alexa Fluor 488 conjugate (Molecular Probes). With respect to isotype controls, rat IgG2b, κ (eB149/10H5, eBioscience, San Diego, CA) and rabbit IgG (Cell Signaling Technology) were utilized for CD45, E-cadherin and HSP47, respectively.

### Immunohistochemistry for frozen tissue sections

Four weeks after BMT, liver and extra-orbital lacrimal glands were collected from the transplant recipient mice. These samples were subsequently frozen in OCT compound without any fixation to furnish fresh frozen blocks. The frozen blocks were then cut into 7μm-thick sections and preserved at -80°C until they were used.

In order to stain CD4 (RM4-5, eBioscience) or CD68 (FA-11, AbD Serotec, Kidlington, UK), the fresh frozen sections were thawed at 37°C, fixed with acetone at RT for 20 min and washed with PBS (3 x 3min).

The sections were then blocked with 10% normal goat serum at RT for 30min. After the serum was rinsed off, the tissue sections were incubated with the primary antibodies at 4°C overnight. The sections were then treated with fluorophore-labelled secondary antibodies at room temperature for 45 minutes and mounted with an anti-fading mounting medium (Fluorescent Mounting Medium; Dako) with 4',6-diamidino-2-phenylindole (DAPI) (Thermo Fischer/Molecular Probes). Fluorescence images were taken with an LSM confocal microscope (Carl Zeiss, Jena, Germany). In this immunohistochemical examination, the following secondary antibodies were used: goat anti-rat IgG (H+L) secondary antibody, Alexa Fluor 488 conjugate (Molecular Probes) and goat anti-rat IgG (H+L) secondary antibody and Alexa Fluor 568 conjugate (Molecular Probes). With respect to isotype controls, rat IgG2a,κ (eBioscience) and rat IgG2a (54447, R&D Systems, Minneapolis, MN) were utilized for CD4 and CD68, respectively.

### Immunoblotting analysis

The tissues of interest were placed in Eppendorf tubes, and pre-cooled RIPA buffer was added to the tubes. The tissues were then homogenized using an electric homogenizer. After the samples were on ice for 1h, they were centrifuged at 15000 rpm at 4°C for 5 min. The supernatants were subsequently collected in fresh tubes on ice and used as cell lysates. An equal amount of 5X Laemmli buffer was added to each cell lysate, followed by protein denaturation at 100°C for 5 min. Equal amounts of protein from each sample were loaded into the wells of SDS-PAGE gels and then resolved. The proteins were transferred from the gels to membranes at 15 V for 20 min. The membranes were blocked with 5% skim milk or 5% BSA in 1 x TBST (a mixture of tris-buffered saline and tween 20) at RT for 1h. The membranes were then incubated with primary antibodies at 4 Covernight. The primary antibodies were diluted 1000 times with 5% skim milk or 5% BSA in 1 x TBST. After the primary antibody incubation, the membranes were washed with 1 x TBST (3 x 10 min), subjected to secondary antibody at RT for 1h and then washed with 1 x TBST (3 x 10 min) and 1 x TBS (2 x 10 min). The proteins of interest were visualized using either of the following two methods. (1) Colorimetric detection of the target proteins was conducted using BCIP/NBT substrate (Promega, WI). (2) Signals were developed with an enhanced chemoluminescence (ECL) detection reagent (GE Healthcare, Littlecalfont, UK), and the target proteins were subsequently visualized with a LAS 4000 mini chemiluminescence imaging system (Fujifilm/GE Healthcare). Densitometric analysis of the obtained protein bands was conducted by the use of the image processing software ImageJ. The primary antibodies used in this experiment were as follows: TXNIP (D5F3E, Cell Signaling Technology), NF-κB (Abcam), 4-HNE (HNEJ-2, Japan Institute for the Control of Aging, Shizuoka, Japan), HSP47 (Stress Gen Biotechnologies Corp), IL-6 (Abcam), CTGF (Abcam) and β-actin (AC-15, Abcam). With regards to the secondary antibodies, (1) when the protein bands were visualized by developing a color, either an alkali phosphatase (AP)-conjugated anti-mouse IgG antibody (Promega) or an AP-conjugated anti-rabbit IgG antibody (Promega) was used, and (2) either an HRP-conjugated anti-mouse antibody (Thermo Fisher Scientific) or an HRP-conjugated anti-rabbit antibody (Thermo Fisher Scientific) was required to detect the target proteins by ECL.

### Enzyme linked immunosorbent assay (ELISA)

Blood was collected from the mice in each group and subsequently centrifuged at 4000 rpm for 10 min. The levels of MCP-1, tumor necrosis factor-α (TNF-α) and interferon-γ (IFN-γ) in the obtained sera were measured utilizing ELISA sets (Becton Dickinson).

In the case of reactive oxygen species (ROS), IL-17, IL-10 and Foxp3, ELISA kits were used: ROS (MyBiosource, San Diego), IL-17 (R & D Systems, Minneapolis), IL-10 (Chondrex, Redmond), Foxp3 (Cloud-Clone, Houston) These assays were conducted according to the protocols provided by the above manufacturers.

### Statistical analysis

Unpaired Student’s t-test was used to determine the statistical significance between the 2 groups of interest. One-way ANOVA was utilized for the 4 groups of interest. Differences are considered significant in the case of P < 0.05. The acquired data are presented as means ± SD.

## Results

### Histological and immunohistochemical investigation into cGVHD-vulnerable organs

**Fig 1:**
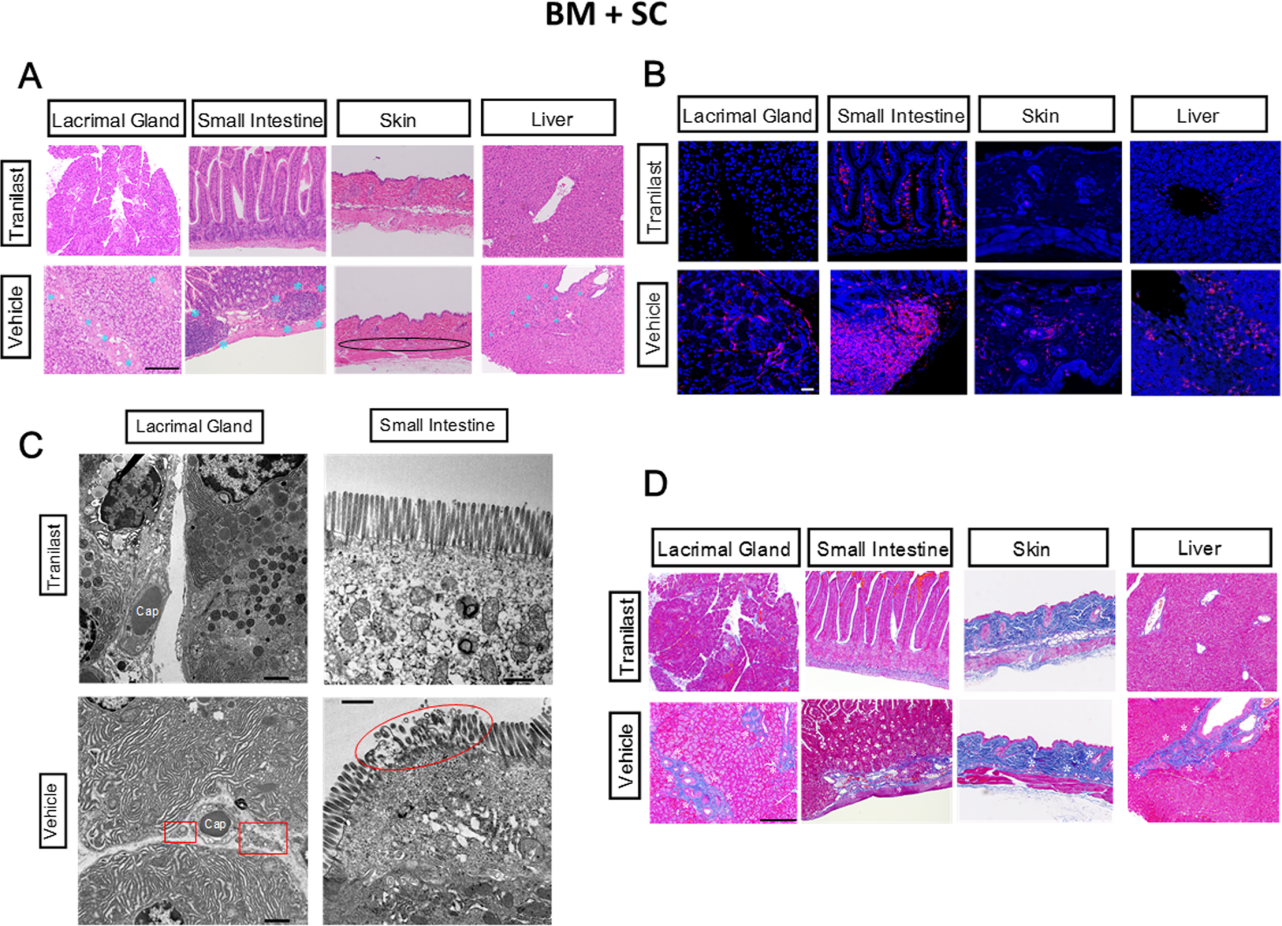
Reduction of cGVHD-triggered systemic inflammation and fibrosis by oral gavage of TL. (A) HE pictures of organs collected from TL- and vehicle-medicated BM+SC recipient mice. The photographs were taken at 200x magnification, and the scale bar is 200 μm. Extensively inflamed portions are shown with blue asterisks. In the picture of the vehicle-medicated skin, loss of fatty tissues was indicated by an ellipse. The enlarged versions of the pictures are shown in **S16 and S17 Figs.** (B) Immunostaining for the generic leukocyte marker CD45 in organs collected from TL- and vehicle-medicated BM+SC recipient mice. Cell membrane and nuclei are stained red and blue, respectively. The images were taken at 200x magnification, and the scale bar is 20 μm. The enlarged versions of the pictures are shown in **S20 and S21 Figs**. (C) Electron micrographs of the lacrimal glands and small intestine collected from TL- and vehicle-medicated BM+SC recipient mice. The pictures of stroma of the lacrimal glands (left) and epithelial cells of the small intestine (right) were taken at 2000x magnification and at 5000x magnification, respectively. The scale bar is 5 μm. Cap: Capillary. In the pictures of the vehicle- medicated lacrimal glands, cell debris is shown with a rectangle. In the photograph of the vehicle-injected small intestine, an ellipse is placed where microvilli were demolished. The enlarged versions of the pictures are shown in **S24 Fig**. (D) Mallory’s staining for organs collected from TL- and vehicle-medicated BM+SC recipient mice. The pictures were taken at 200x magnification, and the scale bar is 200 μm. Excessively fibrotic areas are shown with white asterisks. The enlarged versions of the pictures are shown in **S25 and S26 Figs**. Figures from one of three similar experiments are shown. (A, C, D) BM+SC+TL: n = 10, BM+SC+Vehicle: n = 10.

As stated in the Method section, we treated allo-BMT recipient mice with TL or the solvent vehicle. The first step toward our primary aim was histological analysis of cGVHD-prone organs in the BM + SC recipients. As judged by HE pictures, cGVHD-elicited inflammation was profoundly subdued in the TL-medicated organs in contrast to their vehicle-medicated counterparts (**Fig 1A, S2A and S16–S19 Figs**). Furthermore, immunofluorescence images suggested that there were less immune infiltrates (CD45^+^ cells) in the TL-injected organs compared with the vehicle-injected ones (**Fig 1B, S2B and S20–S23 Figs**). In particular, abnormal influx of CD4^+^ cells and macrophages were suppressed in the TL-treated lacrimal glands and liver (**S3 and S4 Figs**). Moreover, electron micrographs of the lacrimal glands and small intestine indicated (1) that the stroma in the TL-treated lacrimal glands had less cell fragments compared with the vehicle-treated counterparts, (2) that there were more secretary vesicles in the TL-medicated lacrimal glands in comparison to their vehicle-treated partners and (3) the microvilli in the TL-injected small intestine seemed to be almost intact in contrast to those in its vehicle-injected equivalent (**Fig 1C, S24 Fig**). With respect to fibrosis induced by cGVHD, Mallory staining revealed that while the vehicle-treated organs were affected by aberrant fibrosis, their TL-treated equivalents were virtually untouched by cGVHD-triggered fibrosis (**Fig 1D, S2C Fig, and S25–S28 Figs**). Body weight reduction caused by cGVHD was also prevented by TL (**S7 Fig**). In the case of BM-only recipient mice, TL also suppressed abnormal immune cell infiltration, fibrosis and body weight loss in comparison with the solvent-vehicle (**S5, S6 and S29–S40 Figs**). Furthermore, in the BM + SC groups, there was no significant difference in (1) the number of T cells in the spleens or (2) neutrophil counts in peripheral blood collected from the TL- and vehicle-medicated mice. (**S8 and S9 Figs**). These findings suggest that TL could suppress systemic inflammation and fibrosis caused by cGVHD without preventing the engraftment of bone marrow cells.

### Assessment of the degree of inflammation and fibrosis

**Fig 2:**
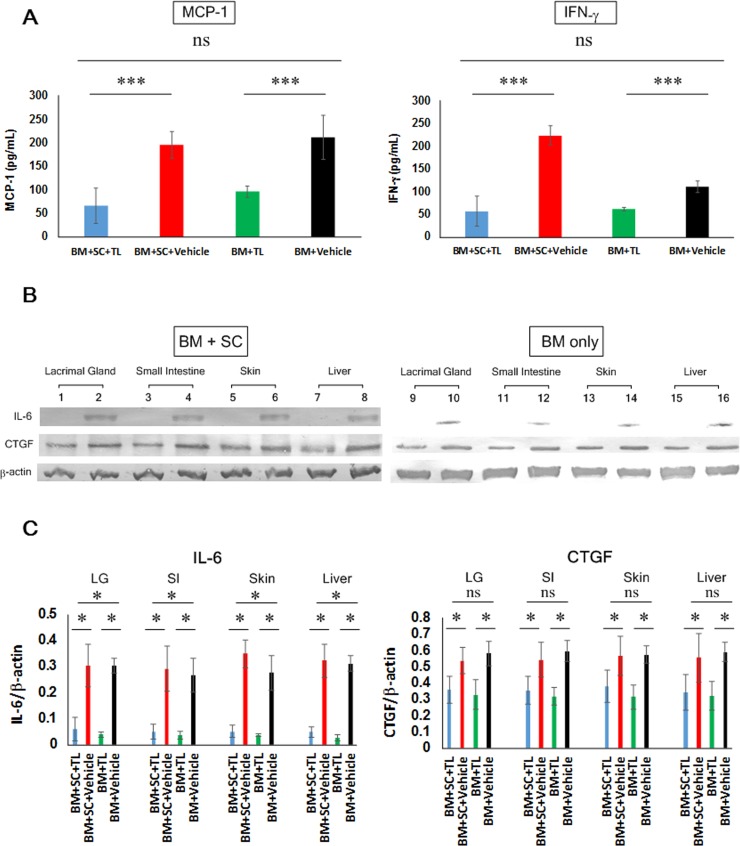
Suppression of inflammatory and fibrotic markers by oral administration of TL. (A) ELISA was carried out to measure the inflammatory indicators MCP-1 and IFN-γ in sera collected from TL-dosed mice (BM+SC+TL: blue, BM+TL: green) and their vehicle-dosed counterparts (BM+SC+Vehicle: red, BM+Vehicle: black) 28 days after BMT. Data from one of two similar experiments are shown. Unpaired Student’s t-test was used to determine the statistical significance between the 2 groups of interest. One-way ANOVA was utilized for the 4 groups of interest. The data are presented as means ± SD, BM+SC+TL: n = 10, BM+SC+Vehicle: n = 10, BM+TL: n = 6, BM+Vehicle: n = 6 ***P<0.001. (B) Immunoblot assays for the inflammatory marker IL-6 and the fibrotic marker CTGF was conducted. (BM+SC Lanes 1, 3, 5, 7: TL-treated organs, Lanes 2, 4, 6, 8: vehicle-treated organs, BM only Lanes 9, 11, 13, 15: TL-treated organs, Lanes 10, 12, 14, 16: vehicle-treated organs) Note: A series of immunoblots is shown in **S10 Fig**, and blots of IL-6, CTGF and β-actin are taken from **S10 Fig**. (C) IL-6 and CTGF in each organ were subsequently quantified by densitometry. TL-injected organs (BM+SC+TL: blue, BM+TL: green) and their vehicle-injected partners (BM+SC+Vehicle: red, BM+Vehicle: black). Data from one of two similar experiments are shown. Unpaired Student’s t-test was used to determine the statistical significance between the 2 groups of interest. One-way ANOVA was utilized for the 4 groups of interest. The data are presented as means ± SD. BM+SC+TL: n = 10, BM+SC+Vehicle: n = 10, BM+TL: n = 6, BM+Vehicle: n = 6. *P<0.05.

Next, we more closely assessed the degree of cGVHD inflammation and fibrosis in the TL- and vehicle-treated allo-BMT recipient mice. Enzyme linked immunosorbent assay (ELISA) revealed that the serum levels of inflammation-associated molecule macrophage chemoattractant protein-1 (MCP-1)[26, 27] and interferon-γ (IFN-γ)[28, 29] was considerably lower in the TL-dosed BM+SC recipients compared with their vehicle-dosed counterparts (**Fig 2A**). As judged by immunoblot analysis, the expression of inflammatory marker interleukin (IL)-6^25^ and fibrotic indicator connective tissue growth factor (CTGF)^26^ was suppressed in the TL-injected organs (BM+SC+TL) in contrast to their vehicle-injected partners (BM+SC+Vehicle) (**Figs 2B and 2C, S10–S12 Figs)**. These findings were also applicable to the BM-only groups (BM+TL vs BM+Vehicle) (**Fig 2A–2C, S10–S12 Figs**) These data also indicated that TL could subdue cGVHD-triggered systemic inflammation and fibrosis.

### Repression of TXNIP, NFκB and oxidative stress

**Fig 3:**
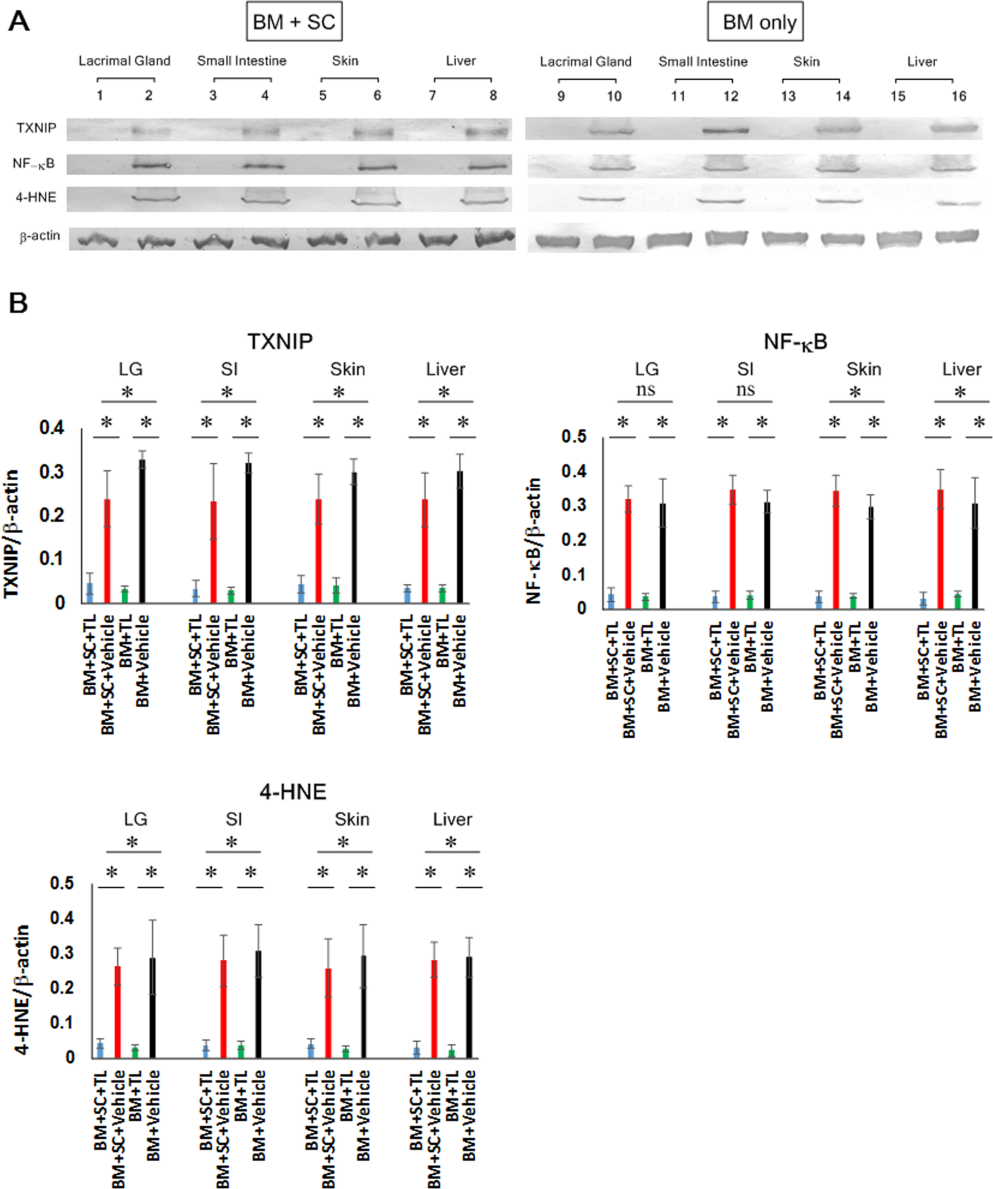
Repression of TXNIP, NF-κB and oxidative stress in cGVHD-susceptible organs by oral administration of TL. (a) Immunoblot investigation into TXNIP, NF-κB and 4-HNE was conducted. (BM+SC Lanes 1, 3, 5, 7: TL-treated organs, Lanes 2, 4, 6, 8: vehicle-treated organs, BM only Lanes 9, 11, 13, 15: TL-treated organs, Lanes 10, 12, 14, 16: vehicle-treated organs) Note: A series of immunoblots is shown in **S10 Fig**, and blots of TXNIP, NF-κB, 4-HNE and β-actin are taken from **S10 Fig**. (b) TXNIP, NF-κB and 4-HNE in the individual organs were subsequently quantified by densitometry. TL-medicated organs (BM+SC+TL: blue, BM+TL: green) and their vehicle-medicated equivalents (BM+SC+Vehicle: red, BM+Vehicle: black). Data from one of two similar experiments are shown. Unpaired Student’s t-test was used to determine the statistical significance between the 2 groups of interest. One-way ANOVA was utilized for the 4 groups of interest. The data are presented as means, ± SD. BM+SC+TL: n = 10, BM+SC+Vehicle: n = 10, BM+TL: n = 6, BM+Vehicle: n = 6. *P<0.05.

Our next task was to investigate the expression and/or activation of the proinflammatory molecules TXNIP and NF-κB in cGVHD-susceptible organs. Immunoblot assays for TXNIP and NF-κB indicated that the expression of TXNIP and activation of NF-κB was profoundly repressed in the TL-medicated organs (BM+SC+TL) in contrast to their vehicle-treated counterparts (BM+SC+Vehicle) (**Fig 3A and 3B, S10, S11 and S13 Figs**). Furthermore, some groups reported that the expression of TXNIP was associated with the elevation of oxidative stress.[30] Thus, immunoblot analysis was carried out to examine the level of oxidative stress marker 4-hydroxynonenal (4-HNE) in cGVHD-vulnerable organs (**Fig 3A and 3B, S10, S11 and S13 Figs**). These observations were also applicable to the BM-only groups (BM+TL vs BM+Vehicle) (**Fig 3A and 3B, S10, S11, and S13 Figs**). Our data indicated that oxidative stress was significantly reduced in the TL-treated organs by contrast with their vehicle-treated partners.

### ROS, IL-17, IL-10 and Foxp3 in sera

**Fig 4:**
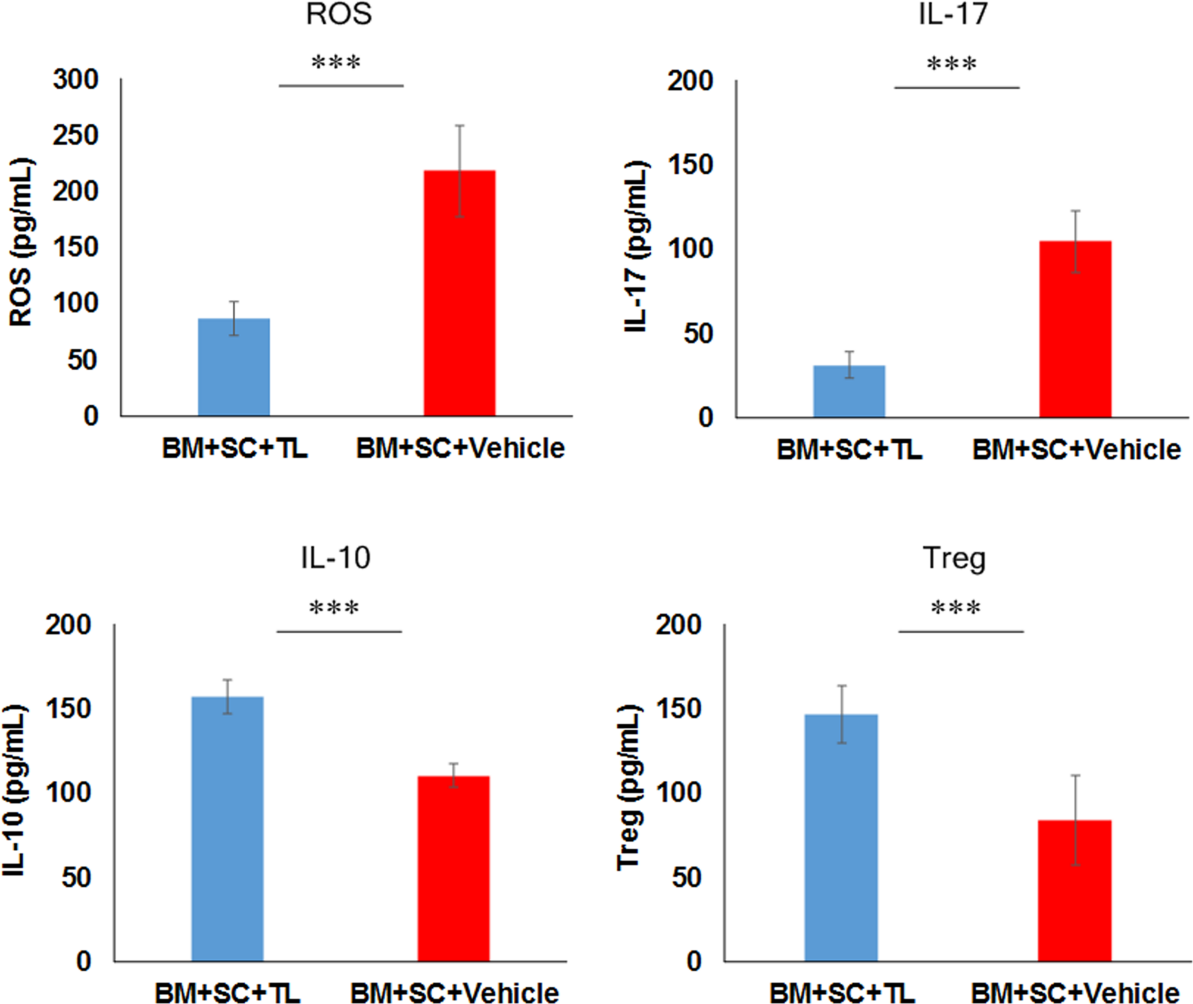
ROS, IL-17, IL-10 and Foxp3 in sera. ELISA was carried out to measure the levels of ROS, IL-17, IL-10 and Foxp3 in sera collected from TL-dosed mice (BM+SC+TL: blue) and their vehicle-dosed counterparts (BM+SC+Vehicle: red) 28 days after BMT. Data from one of two similar experiments are shown. Unpaired Student’s t-test was used to determine the statistical significance between the 2 groups of interest. The data are presented as means ± SD, BM+SC+TL: n = 10, BM+SC+Vehicle: n = 10. ***P<0.001.

We subsequently attempted to examine the levels of ROS, IL-17, IL-10 and Foxp3 in sera collected from the BM+SC recipient mice. The sera from the TL-treated mice showed the lower levels of ROS and IL-17 than those form their vehicle-treated counterparts (**Fig 4**). In contrast, the levels of IL-10 and Foxp3 in the sera from the TL-medicated mice were greater than those from their vehicle-medicated equivalents. (**Fig 4**)

### Suppression of cGVHD-elicited abnormal production of collagen bundles from fibroblasts

**Fig 5:**
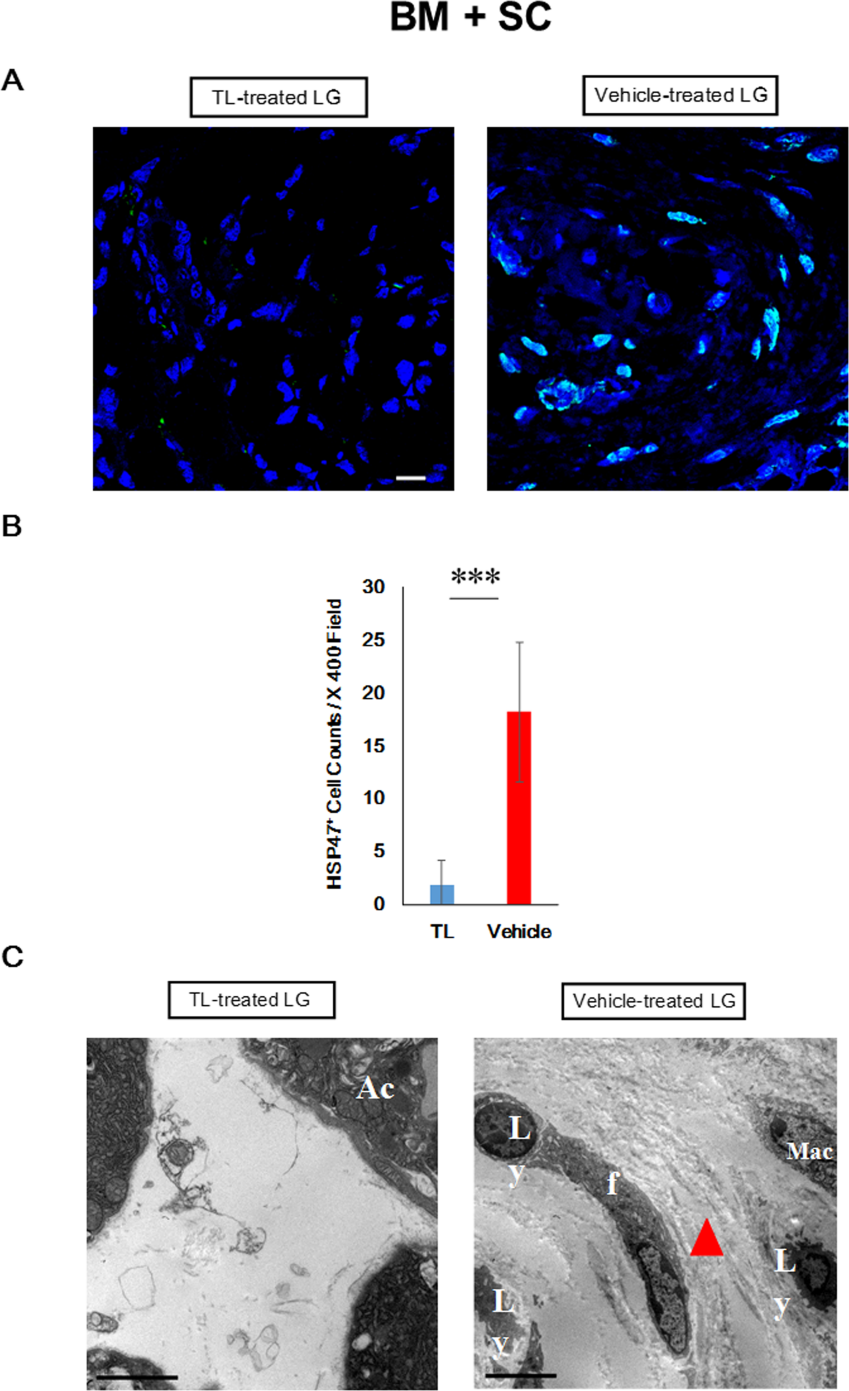
Prevention of cGVHD-induced influx of HSP47^+^ fibroblasts into the lacrimal glands. (a) Immunofluorescence images of HSP47^+^ cells in the lacrimal glands collected from TL- and vehicle-treated BM+SC recipient mice. HSP47 and cell nuclei are stained green and blue, respectively. The images were taken at 400x magnification, and the scale bar is 10 μm. The enlarged versions of the pictures are shown in **S41 Fig**. (b) The density of HSP47^+^ cells in the lacrimal glands collected from TL- and vehicle-treated BM+SC recipient mice. (BM+SC+TL: blue, BM+SC+Vehicle: red) Data from one of two similar experiments are shown. Unpaired Student’s t-test was used to determine the statistical significance between the 2 groups of interest. The data are presented as means, ± SD. BM+SC+TL: n = 3, BM+SC+Vehicle: n = 3. ***P<0.001. (c) Electron micrographs of the lacrimal glands collected from TL- and vehicle-treated BM+SC recipient mice. The pictures were taken at 5000x magnification and at 5000x magnification, respectively. The scale bar is 5 μm. Ly; lymphocyte, f, fibroblast, Mac; macrophage, Ac; Acinus. In the image of the vehicle-medicated lacrimal gland, an aberrant collagen bundle is shown with a red triangle. The enlarged versions of the pictures are shown in **S42 Fig**.

Our next attempt was to gain some insights into how cGVHD-induced fibrosis was repressed by oral administration of TL. Our previous work showed that dysfunctional fibroblasts were implicated in pathogenic fibrosis in lacrimal glands disordered by cGVHD.[31] Thus, we were driven to investigate whether the undesired phenomenon was subdued by TL. Immunefluorescence images suggested that the number of activated fibroblasts (HSP47^+^ cells) in the TL-treated lacrimal glands (BM+SC+TL) was significantly smaller than that in their vehicle-treated counterparts (BM+SC+Vehicle) (**Fig 5A, and 5B, S41 Fig**). Furthermore, electron micrographs indicated that the excessive generation of collagen bundles from fibroblasts was substantially suppressed in the TL-medicated lacrimal glands in contrast to their vehicle-medicated equivalents (**Fig 5C, S42 Fig**).

### Prevention of cGVHD-elicited EMT in multiple organs

**Fig 6:**
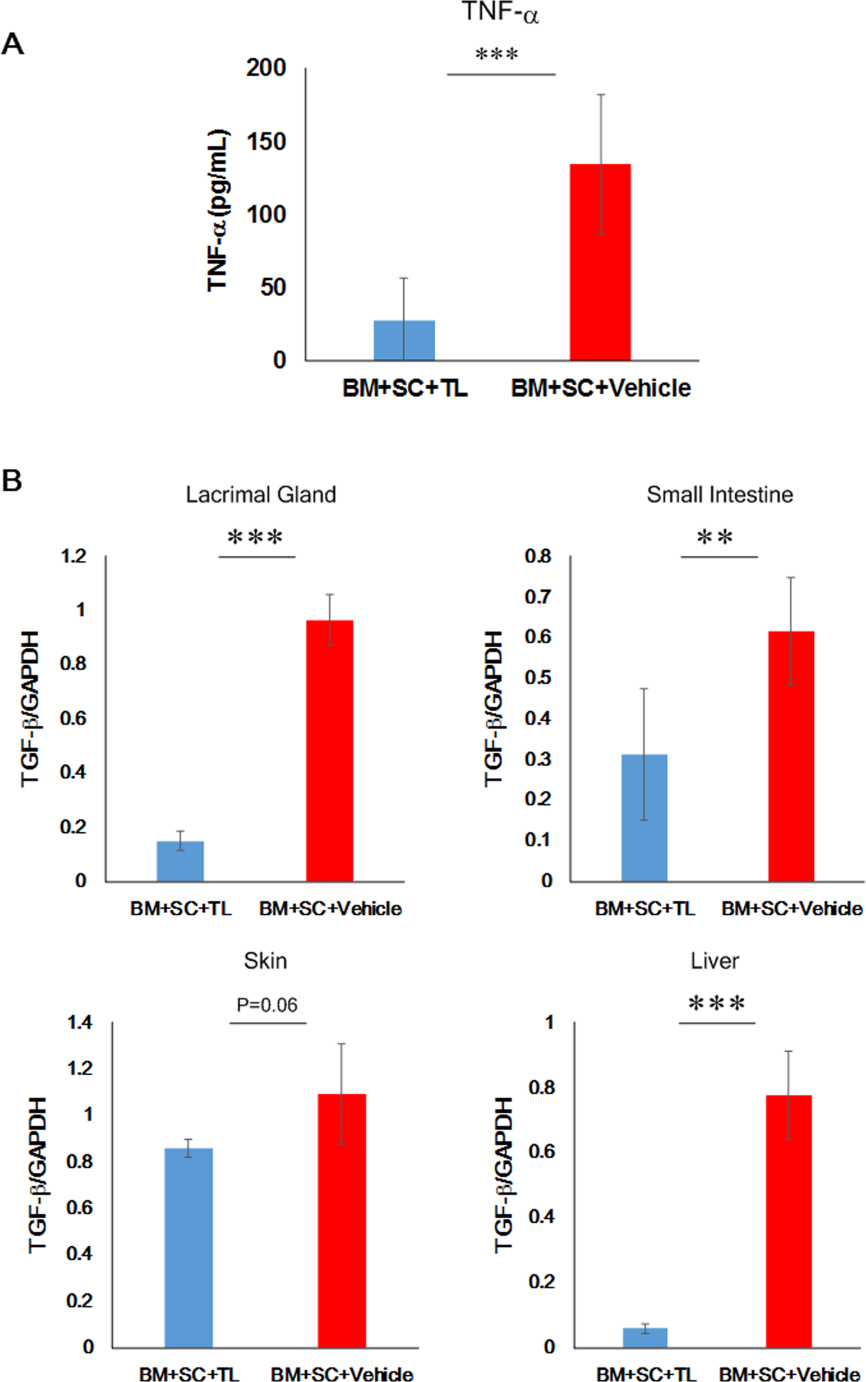
Reduction of TNF-α and TGF-β in organs treated with TL. (a) ELISA was carried out to measure the levels of TNF-α in sera collected from TL-dosed mice (BM+SC+TL: blue) and their vehicle-dosed counterparts (BM+SC+Vehicle: red) 28 days after BMT. Data from one of two similar experiments are shown. Unpaired Student’s t-test was used to determine the statistical significance between the 2 groups of interest. The data are presented as means ± SD. BM+SC+TL: n = 10, BM+SC+Vehicle: n = 10. ***P<0.001. (b) qPCR for TGF-β in TL-medicated organs (BM+SC+TL: blue) and their vehicle-medicated counterparts (BM+SC+Vehicle: red). Data from one of two similar experiments are shown. Unpaired Student’s t-test was used to determine the statistical significance between the 2 groups of interest. The data are presented as means ± SD. BM+SC+TL: n = 4–6, BM+SC+Vehicle n = 5–6. *P<0.05, **P<0.01, ***P<0.001.

**Fig 7:**
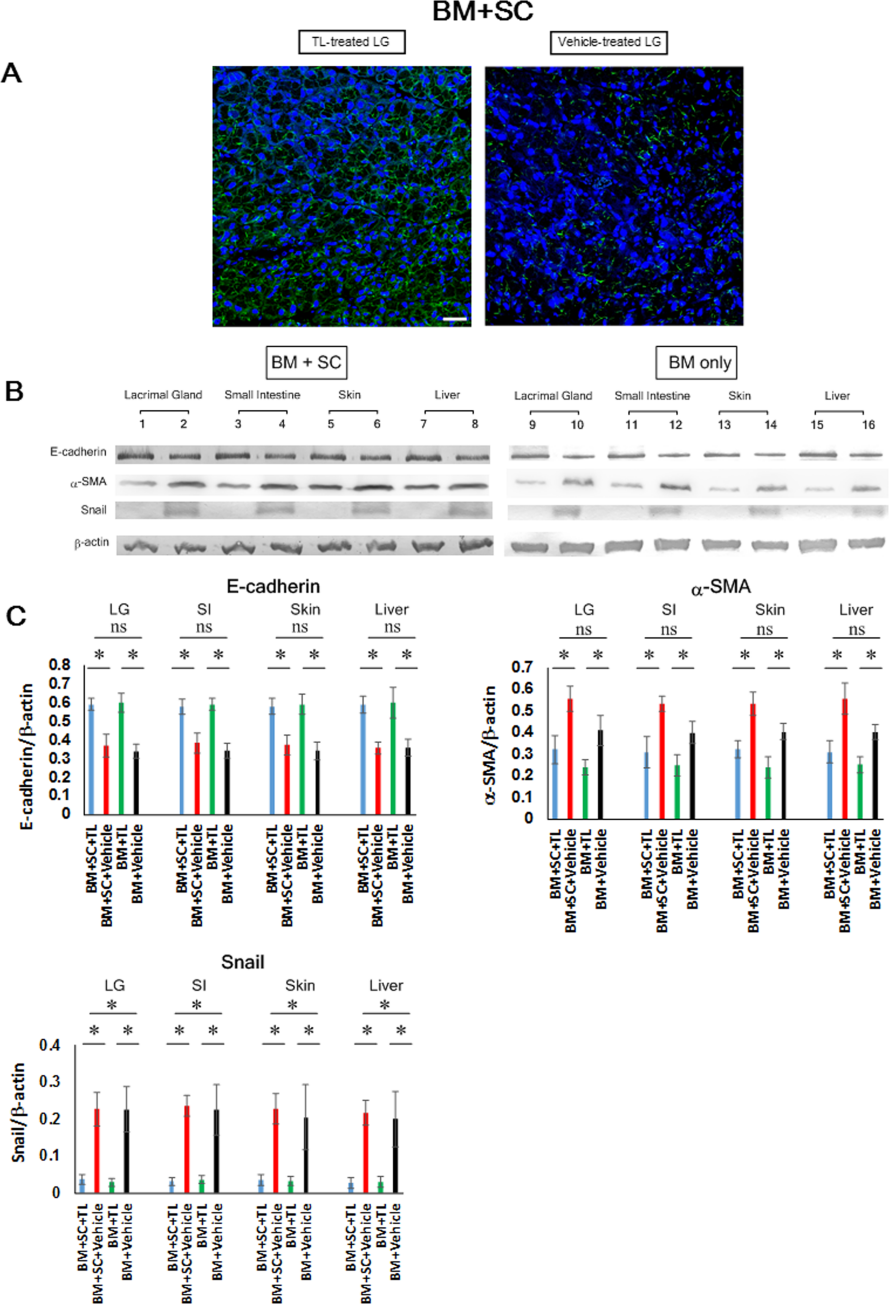
Inhibition of cGVHD-elicited EMT by oral administration of TL. **(**A) Immunofluorescence images of E-cadherin in the lacrimal glands collected from TL- and vehicle-treated BM+SC recipient mice. E-cadherin and cell nuclei are stained green and blue, respectively. The pictures were photographed at 200x magnification, and the scale bar is 20 μm. (B) Immunoblot analysis of E-cadherin, α-SMA and Snail was performed. (BM+SC Lanes 1, 3, 5, 7: TL-treated organs, Lanes 2, 4, 6, 8: vehicle-treated organs, BM only Lanes 9, 11, 13, 15: TL-treated organs, Lanes 10, 12, 14, 16: vehicle-treated organs) Note: A series of immunoblots is shown in **S10 Fig**, and blots of E-cadherin, α-SMA, Snail and β-actin are taken from **S10 Fig**. (C) E-cadherin, α-SMA and Snail in the individual organs were subsequently quantified by densitometry. Unpaired Student’s t-test was used to determine the statistical significance between the 2 groups of interest. One-way ANOVA was utilized for the 4 groups of interest. TL-medicated organs (BM+SC+TL: blue, BM+TL: green) and their vehicle-medicated equivalents (BM+SC+Vehicle: red, BM+Vehicle: black). Data from one of two similar experiments are shown. The data are presented as means, ± SD. BM+SC+TL: n = 10, BM+SC+Vehicle: n = 10, BM+TL: n = 6, BM+Vehicle: n = 6. *P<0.05.

Our other focus was on EMT caused by cGVHD. Reportedly, EMT is implicated in the development of fibrosis triggered by ocular cGVHD,[11] and TL is able to inhibit EMT induced by TNF-α or TGF-β[20] Hence, we investigated the levels of EMT markers and their relevant molecules in the TL- and vehicle-dosed allo-BMT recipient mice. The serum level of TNF-α in the TL-injected mice (BM+SC+TL) was vastly lower than that in their vehicle-injected counterparts (BM+SC+Vehicle) (Fig 6A). Moreover, the mRNA expression of TGF-β in the TL-treated organs (BM+SC+TL) was less than that in their vehicle-treated partners (BM+SC+Vehicle) (Fig 6B; S14 Fig). Thus, we subsequently examined EMT markers in cGVHD-vulnerable organs. As indicated by immunohistochemical and immunoblot analysis, the expression of the EMT marker E-cadherin was maintained in the TL-treated organs in contrast to their vehicle-treated counterparts (BM+SC+TL vs BM+SC+Vehicle and BM+TL vs BM+Vehicle)(**Fig 7A–7C, S10, S11, S15 and S43 Figs**). In addition, although it seems that the protein level of α-SMA in the TL-injected organs was normal, the overexpression of α-SMA was observed in their vehicle-medicated equivalents (BM+SC+TL vs BM+SC+Vehicle and BM+TL vs BM+Vehicle) (**Fig 7B, and 7C, S10, S11, and S15 Figs**). Moreover, Snail was expressed at lower level in the TL-injected organs in comparison to their vehicle-injected counterparts (BM+SC+TL vs BM+SC+Vehicle and BM+TL vs BM+Vehicle) (**Fig 7B and 7C, S10, S11 and S15 Figs**).

## Discussion

To date, allogeneic HSCT (allo-HSCT) has been conducted to treat patients suffering from haematological malignancies. However, the health of allogeneic HSCT recipients can be jeopardized by cGVHD. Since allo-HSCT is increasingly prevalent, the demand of remedies for extensive complications elicited by cGVHD is rising. cGVHD sufferers are usually treated with immunosuppressive agents such as cyclosporine, however the results have been far from satisfactory, because medication with immunosuppressive drugs frequently elicits severe adverse effects including viral infections. Accordingly, clinical settings are eagerly awaiting sophisticated methods to reduce disabling symptoms of cGVHD. A significant component of this proof-of-concept study was the development of the therapy for cGVHD without adverse effects. After our search for effective drugs, we chose TL to combat systemic cGVHD. TL is known to (1) act as an inhibitor of the inflammatory molecules TXNIP and NF-κB and (2) exert anti-fibrotic, anti-EMT and anti-oxidative effects. [17–20] Over and above, the symptoms of cGVHD are similar to those of autoimmune disorders, and tranilast is reportedly effective for autoimmune diseases.[21]

In this study, we investigated experimental cGVHD in mice. As evidenced by our previous studies of chronic GVHD in the human lacrimal glands [31–35], the developmental processes of lacrimal gland cGVHD in this mouse model showed a striking resemblance to those in chronic GVHD patients [5, 10, 23, 36]. The laboratory mice were a powerful research tool to model abnormal inflammation and fibrosis in the human lacrimal glands affected by cGVHD. Moreover, previous articles showed that inflammation and fibrosis were induced systemically in this murine model.[5, 36, 37] Hence, our efforts were undertaken to tackle systemic cGVHD by the use of this well-established mouse model.

Several experiments indicated that when BM + SC recipient mice were orally dosed with TL, abnormal infiltration of immune cells (such as CD4+ cells and macrophages) into tissues, systemic fibrosis and weight loss were reduced. Our previous article indicated that donor-derived bone marrow stromal cells could be a key player in the development of cGVHD and that systemic cGVHD could be induced (BM-Only recipient mice could develop systemic cGVHD).[36] A series of experiments indicated; (1) when BM-only recipients were treated with TL, abnormal immune cell migration, systemic fibrosis and weight reduction were mitigated, (2) there was no significant difference in T cell counts in the spleen collected from TL- and vehicle-treated BM + SC recipients and (3) neutrophils in peripheral blood taken from TL- and vehicle-treated BM + SC recipients were similar in number. Hence, it appears that TL worked for cGVHD, but not by killing donor T cells and that did not disturb the engraftment of bone marrow cells.[38]

Thus, we subsequently attempted to confirm the effectiveness of TL for cGVHD by closely examining the allo-BMT recipient mice. Our results suggested that the cGVHD-induced augmentation of MCP-1 and IFN-γin sera was suppressed by TL. MCP-1 is known to control the trafficking of monocytes/macrophages and whereby be involved in inflammation.[26, 27] Abnormal expression of IFN-γ is reported to be implicated in a wide range of inflammation-related diseases.[28, 29] Moreover, our data suggested that the cGVHD-elicited overexpression of IL-6 and CTGF was profoundly repressed in the organs treated with TL It is conceivable that IL-6 is a chemical signal which plays a decisive role in the differentiation of naïve T cells into regulatory T cells or proinflammatory T cells.[39, 40] Hence, IL-6 can be a dependable marker of inflammation, and many researchers interested in cGVHD have accorded attention to IL-6.^35^ The aberrant expression of CTGF is presumed to play a role in a variety of fibrosis-related diseases, it can be a reliable indicator of fibrosis.[41] These outcomes underpins the efficacy of TL for systemic inflammation and fibrosis caused by cGVHD.

A series of experiments indicated that oral administration of TL could alleviate cGVHD-caused systemic inflammation. Thus, we subsequently investigated whether TL could function as a suppressor of TXNIP, NF-κB and oxidative stress. [17–20] Our investigation into the TL- and vehicle-treated organs indicated that TL showed the capacity to repress the expression of TXNIP and the oxidative stress marker 4-HNE, and the activation of NF-κB in cGVHD-affected organs. These 3 undesired phenomena elicited by cGVHD are presumed to be associated with each other (e.g. the elevation of oxidative stress results in the expression of TXNIP and the activation of NF-κB.),[18, 42] and thereby a vicious cycle can be formed in organs affected by cGVHD. Hence, it is conceivable that TL has the potential to break the negative cycle and whereby subdue cGVHD-triggered inflammation.

As stated above, the level of IL-6 in the TL-treated mice was reduced. Consequently, the Treg level was higher in the TL-treated allo-BMT recipient mice and the IL-17 level is higher in their vehicle-treated counterparts. As with the oxidative stress marker 4-HNE, TL suppressed the level of ROS. Furthermore, TL prevented the decrease of the anti-inflammatory cytokine IL-10.

Several anti-fibrotic therapies for cGVHD have been reported thus far, [43, 44] and our results suggested that TL could be a new addition to them. Thus, we attempted to uncover how TL mitigate fibrosis induced by cGVHD, and our focus was on fibroblasts. Reportedly, dysfunctional fibroblasts are implicated in the progression of fibrosis caused by ocular cGVHD.[31] Thus, immunohistochemical and electron micrographic analysis of fibroblasts in the lacrimal glands was conducted, and our results indicated that TL suppressed the infiltration of HSP47^+^ fibroblasts producing aberrant collagen bundles. This observation can partly account for the prevention of cGVHD-triggered fibrosis by oral administration of TL.

Next, we delved into the efficacy of TL for cGVHD-induced EMT. EMT is a phenomenon in which epithelial cells are deprived of their cell polarity and cell-to-cell adhesion and thereby acquire the migratory properties of mesenchymal stem cells.[11] It has been reported that EMT plays a detrimental role in the developmental processes of fibrosis caused by ocular cGVHD and that TL can restrain TNF-α- and TGF-β-induced EMT.[11, 20] As evidenced by our investigation, when allo-BMT recipients mice were medicated with TL, the increase of TNF-αin sera was suppressed and the mRNA expression of TGF-βin their organs was repressed. Consequently, EMT in their cGVHD-vulnerable organs was subdued. The following EMT markers were addressed in this study: E-cadherin, α-SMA and Snail. Several pieces of evidence demonstrate (1) that deprivation of E-cadherin, which is expressed in epithelial cells, can result in EMT (2) that EMT can arise from overexpression of α-SMA in vascular smooth muscle cells and myoepithelial cells and (3) Snail is conceived to subdue the expression of E-cadherin.[45, 46] As judged by our data, the prevention of EMT by oral administration of TL could also lead to the reduction of cGVHD-elicited fibrosis.

## Conclusion

This proof-of-concept study has suggested that although oral administration of TL may not be able to completely cure cGVHD, it could ameliorate severe symptoms of cGVHD in a safe and effective manner. Furthermore, TL is currently wending its way through clinical trials for cGVHD patients, and the outcomes will be reported in due course. Hence, our novel strategy has the potential to advance the treatment of cGVHD.

## Supporting information

S1 Checklist ARRIVE guidelines checklist. (PDF)

S1 Fig Structure of tranilast (TL). (PDF)

S2 Fig Reduction of cGVHD-triggered systemic inflammation and fibrosis by oral gavage of TL. (A) HE pictures of organs collected from TL- and vehicle-medicated BM+SC recipient mice. The photographs were taken at 200x magnification, and the scale bar is 200 μm. Extensively inflamed portions are shown with blue asterisks. In the picture of the vehicle-medicated eye, arrows were placed where its conjunctiva was severely damaged. The enlarged versions of the pictures are shown in **S18, and S19 Figs.** (B) Immunostaining for the generic leukocyte marker CD45 in organs collected from TL- and vehicle-medicated BM+SC recipient mice. Cell membranes and nuclei are stained red and blue, respectively. The images were taken at 200x magnification, and the scale bar is 20 μm. The enlarged versions of the pictures are shown in **S22 and S23 Figs**. (C) Mallory’s staining for organs collected from TL- and vehicle-medicated BM+SC recipient mice. The pictures were taken at 200x magnification, and the scale bar is 200 μm. Excessively fibrotic areas are shown with white asterisks. The enlarged versions of the pictures are shown in **S28 and S29 Figs**.
(PDF)

S3 Fig Reduction of cGVHD-triggered influx of CD4+ cells by oral gavage of TL. Immunostaining for CD4^+^ cells in the lacrimal glands and liver collected from TL- and vehicle-medicated BM+SC recipient mice. Cell membranes and nuclei are stained red and blue, respectively. The images were taken at 200x magnification, and the scale bar is 20 μm.
(PDF)

S4 Fig Reduction of cGVHD-triggered influx of macrophages by oral gavage of TL. Immunostaining for macrophages in the lacrimal glands and liver collected from TL- and vehicle-medicated BM+SC recipient mice. Cell membranes and nuclei are stained green and blue, respectively. The images were taken at 200x magnification, and the scale bar is 20 μm.
(PDF)

S5 Fig Reduction of cGVHD-triggered systemic inflammation and fibrosis by oral gavage of TL. (A) HE pictures of organs collected from TL- and vehicle-medicated BM-Only recipient mice. The photographs were taken at 200x magnification, and the scale bar is 200 μm. Extensively inflamed portions are shown with blue asterisks. In the picture of the vehicle-medicated skin, loss of fatty tissues was indicated by an ellipse. The enlarged versions of the pictures are shown in **S29 and S30 Figs**. (B) Immunostaining for the generic leukocyte marker CD45 in organs collected from TL- and vehicle-medicated BM-Only recipient mice. Cell membranes and nuclei are stained red and blue, respectively. The images were taken at 200x magnification, and the scale bar is 20 μm. The enlarged versions of the pictures are shown in **S33 and S34 Figs**. (C) Mallory’s staining for organs collected from TL- and vehicle-medicated BM-Only recipient mice. The pictures were taken at 200x magnification, and the scale bar is 200 μm. Excessively fibrotic areas are shown with white asterisks. The enlarged versions of the pictures are shown in **S37 and S38 Figs**.
(PDF)

S6 Fig Reduction of cGVHD-triggered systemic inflammation and fibrosis by oral gavage of TL. (A) HE pictures of organs collected from TL- and vehicle-medicated BM-Only recipient mice. The photographs were taken at 200x magnification, and the scale bar is 200 μm. Extensively inflamed portions are shown with blue asterisks. In the picture of the vehicle-medicated eye, the thinning and decrease of meibomian glands were indicated with a circle. The enlarged versions of the pictures are shown in **S31 and S32 Figs.** (B) Immunostaining for the generic leukocyte marker CD45 in organs collected from TL- and vehicle-medicated BM-Only recipient mice. Cell membranes and nuclei are stained red and blue, respectively. The images were taken at 200x magnification, and the scale bar is 20 μm. The enlarged versions of the pictures are shown in **S35 and S36 Figs**. (C) Mallory’s staining for organs collected from TL- and vehicle-medicated BM-Only recipient mice. The pictures were taken at 200x magnification, and the scale bar is 200 μm. Excessively fibrotic areas are shown with white asterisks. The enlarged versions of the pictures are shown in **S39, and S40 Figs**.
(PDF)

S7 Fig Prevention of body weight loss by TL. (B) The body weight change in a group of TL-dosed BM+SC recipient mice (■), a group of vehicle-dosed BM+SC recipient mice (♦), a group of TL-dosed BM-Only recipient mice (●) and a group of vehicle-dosed BM-Only recipient mice (▲). Unpaired Student’s t-test was used to determine the statistical significance between the 2 groups of interest. One-way ANOVA was utilized for the 4 groups of interest. The values are means ± SD, BM+SC+TL: n = 10, BM+SC+Vehicle: n = 10, BM+TL: n = 6, BM+Vehicle: n = 6. **P<0.01, ***P<0.001.
(PDF)

S8 Fig The number of T cells in the spleen. (a) Flow cytometry was carried out to count T cells in the spleen collected from TL- and vehicle-treated BM+SC recipient mice 28 days after BMT. (BM+SC+TL: blue, BM+SC+Vehicle: red) Data from one of two similar experiments are shown. Unpaired Student’s t-test was used to determine the statistical significance between the 2 groups of interest. The data are presented as means ± SD, BM+SC+TL: n = 5, BM+SC+Vehicle: n = 5
(PDF)

S9 Fig The number of neutrophils in the spleen. (a) Flow cytometry was carried out to count neutrophils in peripheral blood collected from TL- and vehicle-treated BM+SC recipient mice 21 days after BMT. (BM+SC+TL: blue, BM+SC+Vehicle: red) Data from one of two similar experiments are shown. Unpaired Student’s t-test was used to determine the statistical significance between the 2 groups of interest. The data are presented as means ± SD, BM+SC+TL: n = 4, BM+SC+Vehicle: n = 4
(PDF)

S10 Fig Immunoblot analysis of the proteins of interest. Immunoblot assays for IL-6, CTGF, TXNIP, NF-κB, 4-HNE, E-cadherin, α-SMA, Snail and β-actin in the individual samples was conducted. (BM+SC Lanes 1, 3, 5, 7: TL-treated organs, Lanes 2, 4, 6, 8: vehicle-treated organs, BM only Lanes 9, 11, 13, 15: TL-treated organs, Lanes 10, 12, 14, 16: vehicle-treated organs).
(PDF)

S11 Fig Immunoblot analysis of the proteins of interest. Immunoblot assays for IL-6, CTGF, TXNIP, NF-κB, 4-HNE, E-cadherin, α-SMA, Snail and β-actin in the individual samples was conducted. (BM+SC Lanes 1, 3, 5: TL-treated organs, Lanes 2, 4, 6: vehicle-treated organs, BM only Lanes 7, 9, 11: TL-treated organs, Lanes 8, 10, 12: vehicle-treated organs).
(PDF)

S12 Fig Suppression of inflammatory and fibrotic markers by oral administration of TL. **(A)** Immunoblot assays for the inflammatory marker IL-6 and the fibrotic marker CTGF was conducted. (BM+SC Lanes 1, 3, 5: TL-treated organs, Lanes 2, 4, 6: vehicle-treated organs, BM only Lanes 7, 9, 11: TL-treated organs, Lanes 8, 10, 12: vehicle-treated organs) Note: A series of immunoblots is shown in **S11 Fig**, and blots of IL-6, CTGF and β-actin are taken from **S11 Fig**. (C) IL-6 and CTGF in each organ were subsequently quantified by densitometry. TL-injected organs (BM+SC+TL: blue, BM+TL: green) and their vehicle-injected partners (BM+SC+Vehicle: red, BM+Vehicle: black). Data from one of two similar experiments are shown. Unpaired Student’s t-test was used to determine the statistical significance between the 2 groups of interest. One-way ANOVA was utilized for the 4 groups of interest. The data are presented as means ± SD. BM+SC+TL: n = 8, BM+SC+Vehicle: n = 8, BM+TL: n = 6, BM+Vehicle: n = 6. *P<0.05.
(PDF)

S13 Fig Repression of TXNIP, NF-κB and oxidative stress in cGVHD-susceptible organs by oral administration of TL. (A) Immunoblot investigation into TXNIP, NF-κB and 4-HNE was conducted. (BM+SC Lanes 1, 3, 5: TL-treated organs, Lanes 2, 4, 6: vehicle-treated organs, BM only Lanes 7, 9, 11: TL-treated organs, Lanes 8, 10, 12: vehicle-treated organs) Note: A series of immunoblots is shown in **S11 Fig**, and blots of TXNIP, NF-κB, 4-HNE and β-actin are taken from **S11 Fig**. (C) TXNIP, NF-κB, 4-HNE in each organ were subsequently quantified by densitometry. TL-injected organs (BM+SC+TL: blue, BM+TL: green) and their vehicle-injected partners (BM+SC+Vehicle: red, BM+Vehicle: black). Data from one of two similar experiments are shown. Unpaired Student’s t-test was used to determine the statistical significance between the 2 groups of interest. One-way ANOVA was utilized for the 4 groups of interest. The data are presented as means ± SD. BM+SC+TL: n = 8, BM+SC+Vehicle: n = 8, BM+TL: n = 6, BM+Vehicle: n = 6. *P<0.05.
(PDF)

S14 Fig Reduction of TGF-β in organs treated with TL. qPCR for TGF-β in TL-medicated organs (BM+SC+TL: blue) and their vehicle-medicated counterparts (BM+SC+Vehicle: red). Data from one of two similar experiments are shown. Unpaired Student’s t-test was used to determine the statistical significance between the 2 groups of interest. The data are presented as means ± SD. BM+SC+TL: n = 4–6, BM+SC+Vehicle n = 5–6. *P<0.05, **P<0.01, ***P<0.001.
(PDF)

S15 Fig Inhibition of cGVHD-elicited EMT by oral administration of TL. (A) Immunoblot analysis of E-cadherin, α-SMA and Snail was performed. (BM+SC Lanes 1, 3, 5: TL-treated organs, Lanes 2, 4, 6: vehicle-treated organs, BM only Lanes 7, 9, 11: TL-treated organs, Lanes 8, 10, 12: vehicle-treated organs) Note: A series of immunoblots is shown in **S11 Fig**, and blots of E-cadherin, α-SMA, Snail and β-actin are taken from **S11 Fig**. (C) TXNIP, NF-κB, 4-HNE in each organ were subsequently quantified by densitometry. TL-injected organs (BM+SC+TL: blue, BM+TL: green) and their vehicle-injected partners (BM+SC+Vehicle: red, BM+Vehicle: black). Data from one of two similar experiments are shown. Unpaired Student’s t-test was used to determine the statistical significance between the 2 groups of interest. One-way ANOVA was utilized for the 4 groups of interest. The data are presented as means ± SD. BM+SC+TL: n = 8, BM+SC+Vehicle: n = 8, BM+TL: n = 6, BM+Vehicle: n = 6. *P<0.05.
(PDF)

S16 Fig The enlarged versions of the HE pictures of the lacrimal glands and small intestine collected from TL- and vehicle-medicated BM+SC recipient mice shown in Fig 1A. The images were taken at 200x magnification, and the scale bar is 200 μm. Severely inflamed portions are shown with blue asterisks.
(PDF)

S17 Fig The enlarged versions of the HE pictures of the skin and liver collected from TL- and vehicle-medicated BM+SC recipient mice shown in Fig 1A. The images were taken at 200x magnification, and the scale bar is 200 μm. Severely inflamed portions are shown with blue asterisks. In the picture of the vehicle-medicated skin, an ellipse is placed where the fatty tissues were lost.
(PDF)

S18 Fig The enlarged versions of the HE pictures of the salivary glands and lung collected from TL- and vehicle-medicated BM+SC recipient mice shown in S2A Fig. The images were taken at 200x magnification, and the scale bar is 200 μm. Severely inflamed portions are shown with blue asterisks.
(PDF)

S19 Fig The enlarged versions of the HE pictures of the large intestine and eye collected from TL- and vehicle-medicated BM+SC recipient mice shown in S2A Fig. The images were taken at 200x magnification, and the scale bar is 200 μm. Severely inflamed portions are shown with blue asterisks. In the picture of the vehicle-medicated eye, arrows were placed where its conjunctiva was severely damaged.
(PDF)

S20 Fig The enlarged versions of the fluorescence images of the lacrimal glands and small intestine collected from TL- and vehicle-medicated BM+SC recipient mice shown in Fig 2B. CD45 and cell nuclei are stained red and blue, respectively. The images were taken at 200x magnification, and the scale bar is 20 μm.
(PDF)

S21 Fig The enlarged versions of the fluorescence images of the skin and liver collected from TL- and vehicle-medicated BM+SC recipient mice shown in Fig 2B. CD45 and cell nuclei are stained red and blue, respectively. The images were taken at 200x magnification, and the scale bar is 20 μm.
(PDF)

S22 Fig The enlarged versions of the fluorescence images of the salivary glands and lung collected from TL- and vehicle-medicated BM+SC recipient mice shown in S2B Fig. CD45 and cell nuclei are stained red and blue, respectively. The images were taken at 200x magnification, and the scale bar is 20 μm.
(PDF)

S23 Fig The enlarged versions of the fluorescence images of the large intestine and eye collected from TL- and vehicle-medicated BM+SC recipient mice shown in S2B Fig. CD45 and cell nuclei are stained red and blue, respectively. The images were taken at 200x magnification, and the scale bar is 20 μm.
(PDF)

S24 Fig The enlarged versions of the electron micrographs of the lacrimal glands and small intestine collected from TL- and vehicle-medicated BM+SC recipient mice shown in Fig 1C. The pictures of stroma of the lacrimal glands (left) and epithelial cells of the small intestine (right) were taken at 2000x magnification and at 5000x magnification, respectively. The scale bar is 5 μm. Cap: Capillary. In the pictures of the vehicle-medicated lacrimal glands, cell debris is shown with a rectangle. In the photograph of the vehicle-injected small intestine, an ellipse is placed where microvilli were demolished.
(PDF)

S25 Fig The enlarged versions of the Mallory pictures of the lacrimal glands and small intestine collected from TL- and vehicle-medicated BM+SC recipient mice shown in Fig 1D. The pictures were taken at 200x magnification, and the scale bar is 200 μm. Excessively fibrotic areas are shown with white asterisks.
(PDF)

S26 Fig The enlarged versions of the Mallory pictures of the skin and liver collected from TL- and vehicle-medicated BM+SC recipient mice shown in Fig 1D. The pictures were taken at 200x magnification, and the scale bar is 200 μm. Excessively fibrotic areas are shown with white asterisks.
(PDF)

S27 Fig The enlarged versions of the Mallory pictures of the salivary glands and lung collected from TL- and vehicle-medicated BM+SC recipient mice shown in S2C Fig. The pictures were taken at 200x magnification, and the scale bar is 200 μm. Excessively fibrotic areas are shown with white asterisks.
(PDF)

S28 Fig The enlarged versions of the Mallory pictures of the large intestine and eye collected from TL- and vehicle-medicated BM+SC recipient mice shown in S2C Fig. The pictures were taken at 200x magnification, and the scale bar is 200 μm. Excessively fibrotic areas are shown with white asterisks.
(PDF)

S29 Fig The enlarged versions of the HE pictures of the lacrimal glands and small intestine collected from TL- and vehicle-medicated BM-Only recipient mice shown in S5A Fig. The images were taken at 200x magnification, and the scale bar is 200 μm. Severely inflamed portions are shown with blue asterisks.
(PDF)

S30 Fig The enlarged versions of the HE pictures of the skin and liver collected from TL- and vehicle-medicated BM-Only recipient mice shown in S5A Fig. The images were taken at 200x magnification, and the scale bar is 200 μm. Severely inflamed portions are shown with blue asterisks. In the picture of the vehicle-medicated skin, loss of fatty tissues was indicated by an ellipse.
(PDF)

S31 Fig The enlarged versions of the HE pictures of the salivary glands and lung collected from TL- and vehicle-medicated BM-Only recipient mice shown in S6A Fig. The images were taken at 200x magnification, and the scale bar is 200 μm. Severely inflamed portions are shown with blue asterisks.
(PDF)

S32 Fig The enlarged versions of the HE pictures of the large intestine and eye collected from TL- and vehicle-medicated BM-Only recipient mice shown in S6A Fig. The images were taken at 200x magnification, and the scale bar is 200 μm. Severely inflamed portions are shown with blue asterisks. In the picture of the vehicle-medicated eye, the thinning and decrease of meibomian glands were indicated with a circle.
(PDF)

S33 Fig The enlarged versions of the fluorescence images of the lacrimal glands and small intestine collected from TL- and vehicle-medicated BM-Only recipient mice shown in S5A Fig. CD45 and cell nuclei are stained red and blue, respectively. The images were taken at 200x magnification, and the scale bar is 20 μm.
(PDF)

S34 Fig The enlarged versions of the fluorescence images of the skin and liver collected from TL- and vehicle-medicated BM-Only recipient mice shown in S5A Fig. CD45 and cell nuclei are stained red and blue, respectively. The images were taken at 200x magnification, and the scale bar is 20 μm.
(PDF)

S35 Fig The enlarged versions of the fluorescence images of the salivary glands and lung collected from TL- and vehicle-medicated BM-Only recipient mice shown in S6B Fig. CD45 and cell nuclei are stained red and blue, respectively. The images were taken at 200x magnification, and the scale bar is 20 μm.
(PDF)

S36 Fig The enlarged versions of the fluorescence images of the large intestine and eye collected from TL- and vehicle-medicated BM-Only recipient mice shown in S6B Fig. CD45 and cell nuclei are stained red and blue, respectively. The images were taken at 200x magnification, and the scale bar is 20 μm.
(PDF)

S37 Fig The enlarged versions of the Mallory pictures of the lacrimal glands and small intestine collected from TL- and vehicle-medicated BM-Only recipient mice shown in S5C Fig. The pictures were taken at 200x magnification, and the scale bar is 200 μm. Excessively fibrotic areas are shown with white asterisks.
(PDF)

S38 Fig The enlarged versions of the Mallory pictures of the skin and liver collected from TL- and vehicle-medicated BM-Only recipient mice shown in S5C Fig. The pictures were taken at 200x magnification, and the scale bar is 200 μm. Excessively fibrotic areas are shown with white asterisks.
(PDF)

S39 Fig The enlarged versions of the Mallory pictures of the salivary glands and lung collected from TL- and vehicle-medicated BM-Only recipient mice shown in S6C Fig. The pictures were taken at 200x magnification, and the scale bar is 200 μm. Excessively fibrotic areas are shown with white asterisks.
(PDF)

S40 Fig The enlarged versions of the Mallory pictures of the large intestine and eye collected from TL- and vehicle-medicated BM-Only recipient mice shown in S6C Fig. The pictures were taken at 200x magnification, and the scale bar is 200 μm. Excessively fibrotic areas are shown with white asterisks.
(PDF)

S41 Fig The enlarged versions of the fluorescence images of the lacrimal glands collected from TL- and vehicle-medicated BM+SC recipient mice shown in Fig 5A. HSP47 and cell nuclei are stained green and blue, respectively. The images were taken at 400x magnification, and the scale bar is 10 μm.
(PDF)

S42 Fig The enlarged versions of the fluorescence images of the lacrimal glands collected from TL- and vehicle-medicated BM+SC recipient mice shown in Fig 5C. The pictures were taken at 5000x magnification and at 5000x magnification, respectively. The scale bar is 5 μm. Ly; lymphocyte, f, fibroblast, Mac; macrophage, Ac; Acinus. In the image of the vehicle-medicated lacrimal gland, an aberrant collagen bundle is shown with a red triangle.
(PDF)

S43 Fig The enlarged versions of the fluorescence images of the lacrimal glands collected from TL- and vehicle-medicated BM+SC recipient mice shown in Fig 7A. E-cadherin and cell nuclei are stained green and blue, respectively. The pictures were photographed at 200x magnification, and the scale bar is 20 μm.
(PDF)
