# Supplementary figures and images for: Therapeutic potential of tranilast for the treatment of chronic graft-versus-host disease in mice
Source: PLoS One. 2018 Oct 11;13(10):e0203742. doi: 10.1371/journal.pone.0203742 (PMC6181285; doi:10.1371/journal.pone.0203742)

S1 Fig.

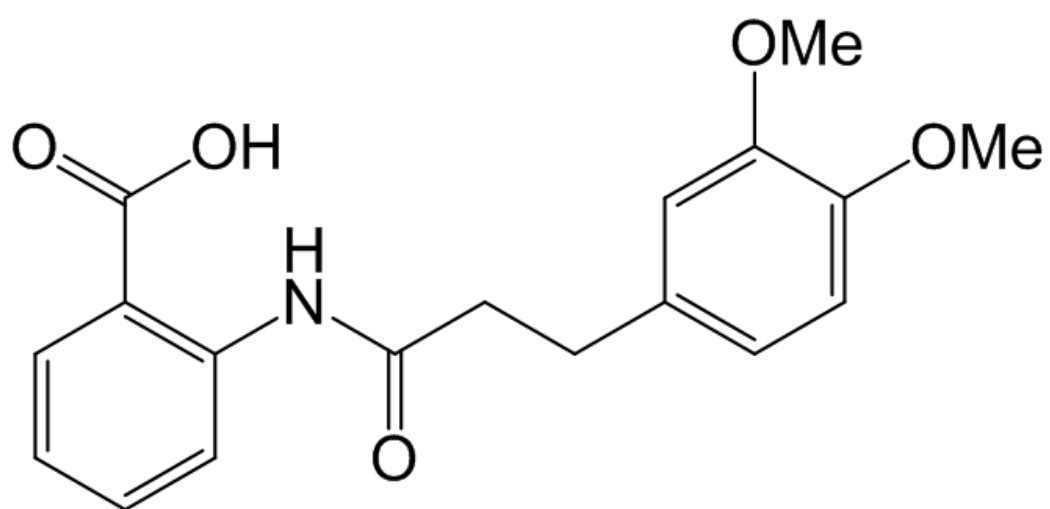

Tranilast

Supplement: S1 Fig — (PDF) [file pone.0203742.s002.pdf]

S2 Fig.

BM+SC

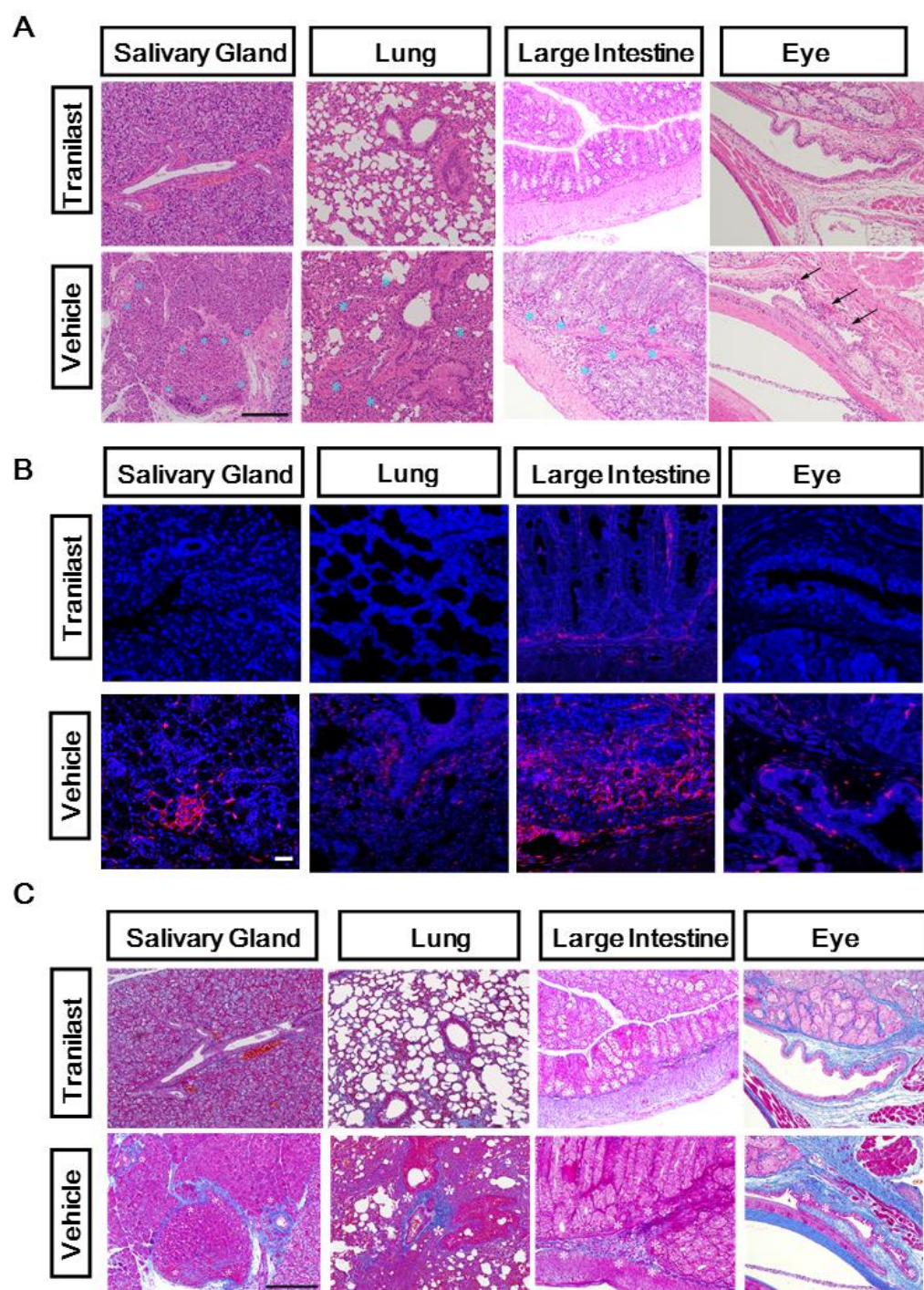

Supplement: S2 Fig — (A) HE pictures of organs collected from TL- and vehicle-medicated BM+SC recipient mice. The photographs were taken at 200x magnification, and the scale bar is 200 μm. Extensively inflamed portions are shown with blue asterisks. In the picture of the vehicle-medicated eye, arrows were placed where its conjunctiva was severely damaged. The enlarged versions of the pictures are shown in S18, and S19 Figs. (B) Immunostaining for the generic leukocyte marker CD45 in organs collected from TL- and vehicle-medicated BM+SC recipient mice. Cell membranes and nuclei are stained red and blue, respectively. The images were taken at 200x magnification, and the scale bar is 20 μm. The enlarged versions of the pictures are shown in S22 and S23 Figs. (C) Mallory’s staining for organs collected from TL- and vehicle-medicated BM+SC recipient mice. The pictures were taken at 200x magnification, and the scale bar is 200 μm. Excessively fibrotic areas are shown with white asterisks. The enlarged versions of the pictures are shown in S28 and S29 Figs. (PDF) [file pone.0203742.s003.pdf]

S3 Fig.

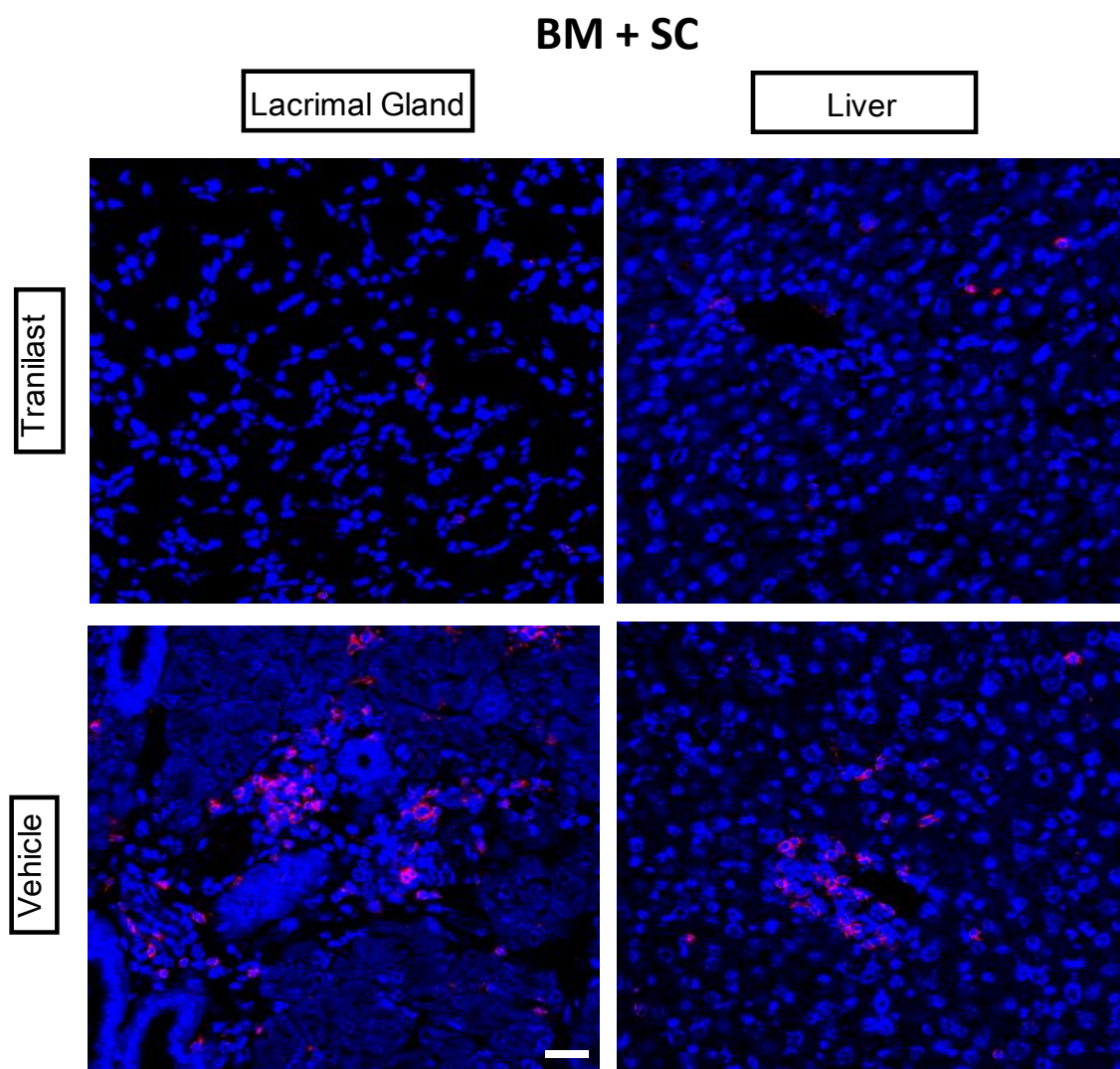

Supplement: S3 Fig — Immunostaining for CD4+ cells in the lacrimal glands and liver collected from TL- and vehicle-medicated BM+SC recipient mice. Cell membranes and nuclei are stained red and blue, respectively. The images were taken at 200x magnification, and the scale bar is 20 μm. (PDF) [file pone.0203742.s004.pdf]

**S4 Fig.**

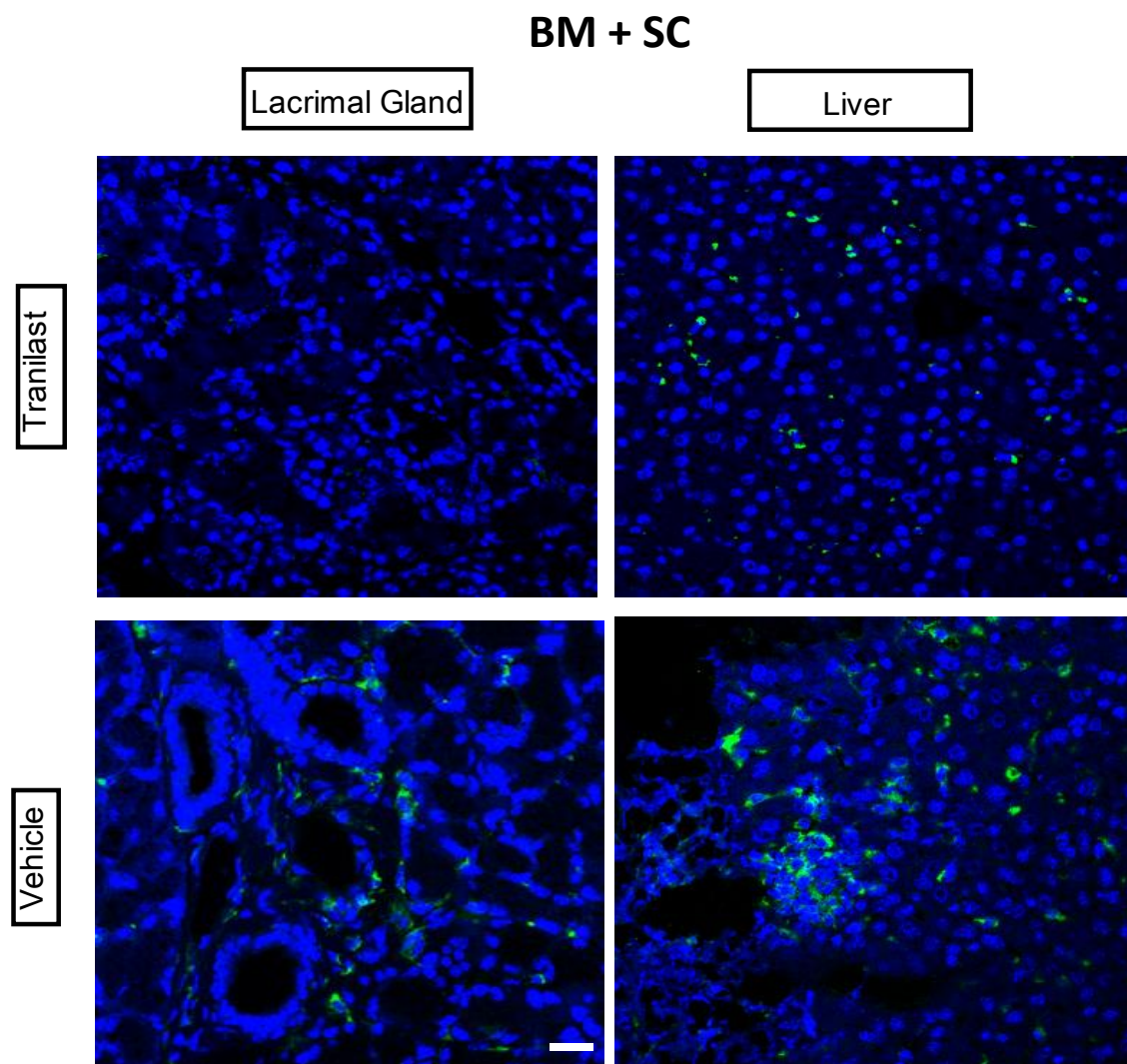

Supplement: S4 Fig — Immunostaining for macrophages in the lacrimal glands and liver collected from TL- and vehicle-medicated BM+SC recipient mice. Cell membranes and nuclei are stained green and blue, respectively. The images were taken at 200x magnification, and the scale bar is 20 μm. (PDF) [file pone.0203742.s005.pdf]

**S5 Fig.**

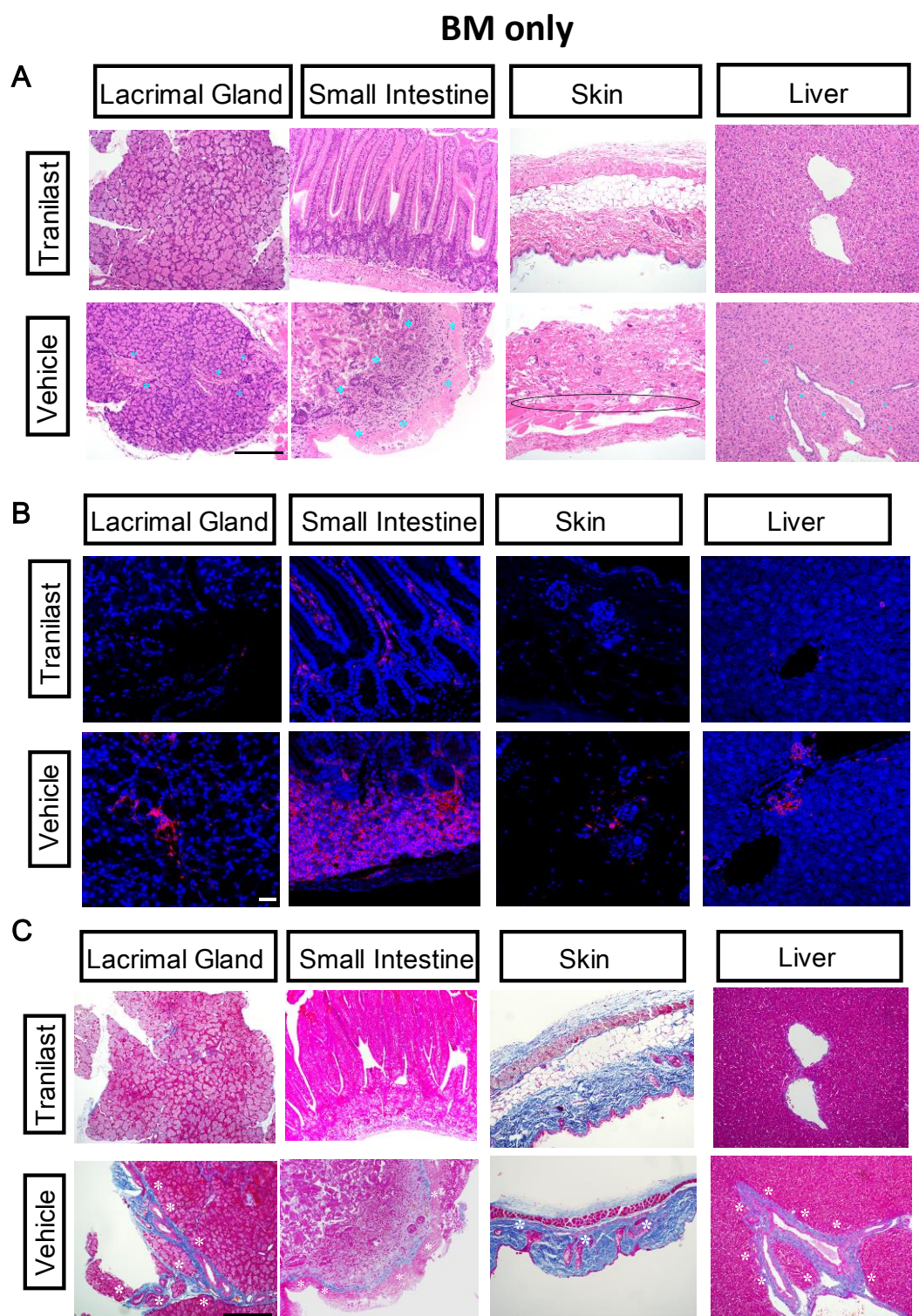

Supplement: S5 Fig — (A) HE pictures of organs collected from TL- and vehicle-medicated BM-Only recipient mice. The photographs were taken at 200x magnification, and the scale bar is 200 μm. Extensively inflamed portions are shown with blue asterisks. In the picture of the vehicle-medicated skin, loss of fatty tissues was indicated by an ellipse. The enlarged versions of the pictures are shown in S29 and S30 Figs. (B) Immunostaining for the generic leukocyte marker CD45 in organs collected from TL- and vehicle-medicated BM-Only recipient mice. Cell membranes and nuclei are stained red and blue, respectively. The images were taken at 200x magnification, and the scale bar is 20 μm. The enlarged versions of the pictures are shown in S33 and S34 Figs. (C) Mallory’s staining for organs collected from TL- and vehicle-medicated BM-Only recipient mice. The pictures were taken at 200x magnification, and the scale bar is 200 μm. Excessively fibrotic areas are shown with white asterisks. The enlarged versions of the pictures are shown in S37 and S38 Figs. (PDF) [file pone.0203742.s006.pdf]

S6 Fig.

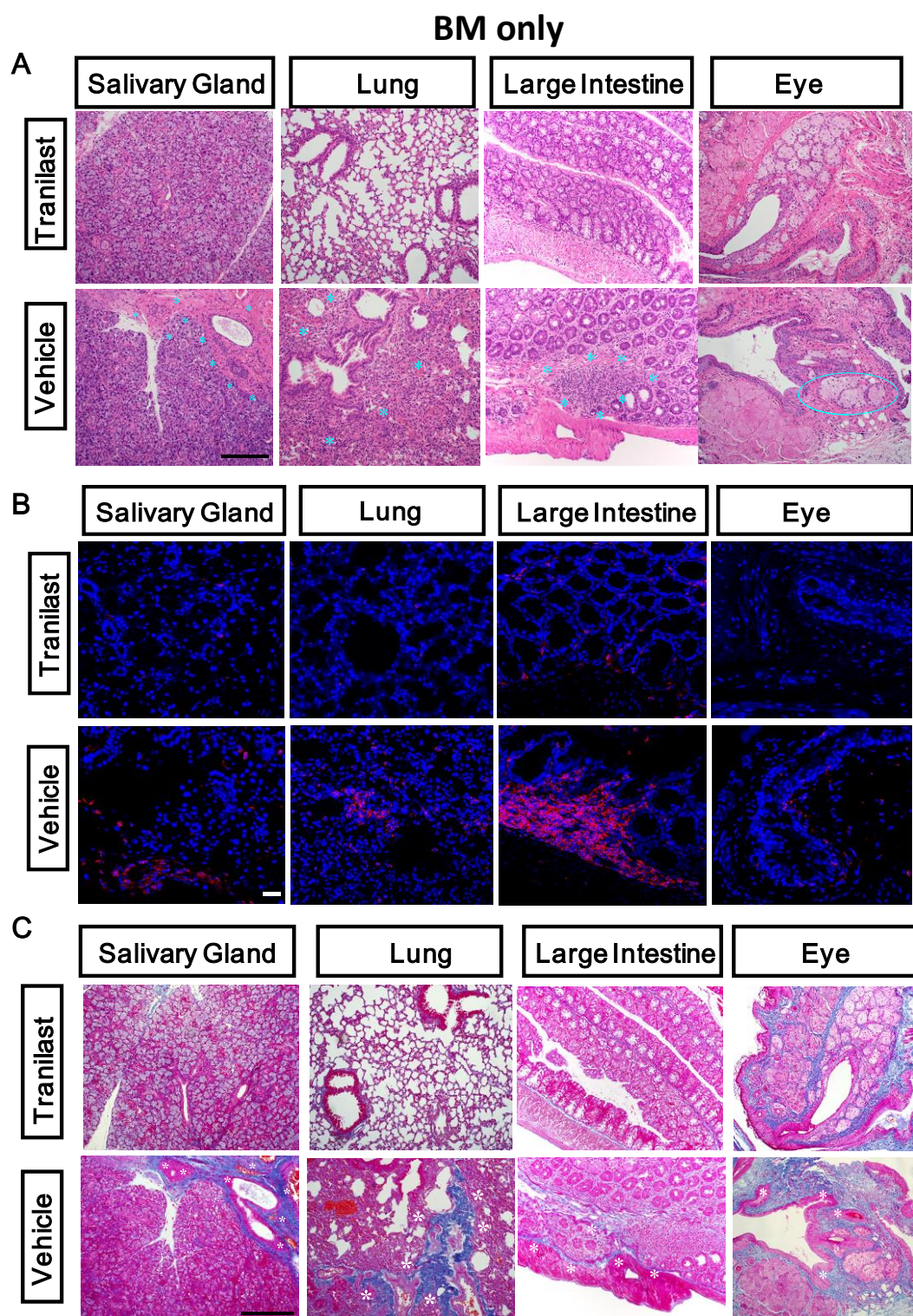

Supplement: S6 Fig — (A) HE pictures of organs collected from TL- and vehicle-medicated BM-Only recipient mice. The photographs were taken at 200x magnification, and the scale bar is 200 μm. Extensively inflamed portions are shown with blue asterisks. In the picture of the vehicle-medicated eye, the thinning and decrease of meibomian glands were indicated with a circle. The enlarged versions of the pictures are shown in S31 and S32 Figs. (B) Immunostaining for the generic leukocyte marker CD45 in organs collected from TL- and vehicle-medicated BM-Only recipient mice. Cell membranes and nuclei are stained red and blue, respectively. The images were taken at 200x magnification, and the scale bar is 20 μm. The enlarged versions of the pictures are shown in S35 and S36 Figs. (C) Mallory’s staining for organs collected from TL- and vehicle-medicated BM-Only recipient mice. The pictures were taken at 200x magnification, and the scale bar is 200 μm. Excessively fibrotic areas are shown with white asterisks. The enlarged versions of the pictures are shown in S39, and S40 Figs. (PDF) [file pone.0203742.s007.pdf]

S7 Fig.

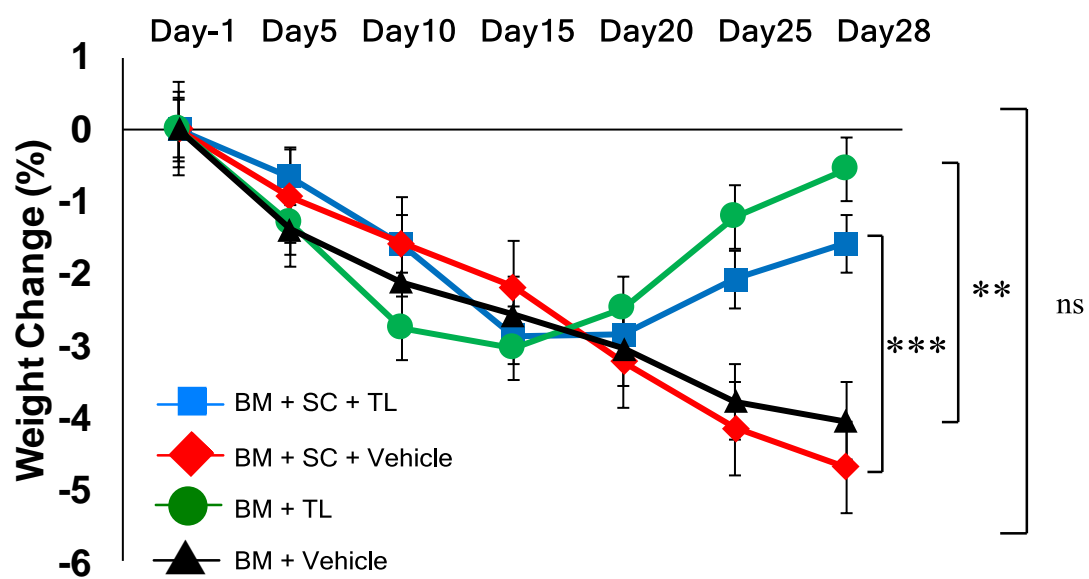

Supplement: S7 Fig — (B) The body weight change in a group of TL-dosed BM+SC recipient mice (■), a group of vehicle-dosed BM+SC recipient mice (♦), a group of TL-dosed BM-Only recipient mice (●) and a group of vehicle-dosed BM-Only recipient mice (▲). Unpaired Student’s t-test was used to determine the statistical significance between the 2 groups of interest. One-way ANOVA was utilized for the 4 groups of interest. The values are means ± SD, BM+SC+TL: n = 10, BM+SC+Vehicle: n = 10, BM+TL: n = 6, BM+Vehicle: n = 6. **P<0.01, ***P<0.001. (PDF) [file pone.0203742.s008.pdf]

**S8 Fig.**

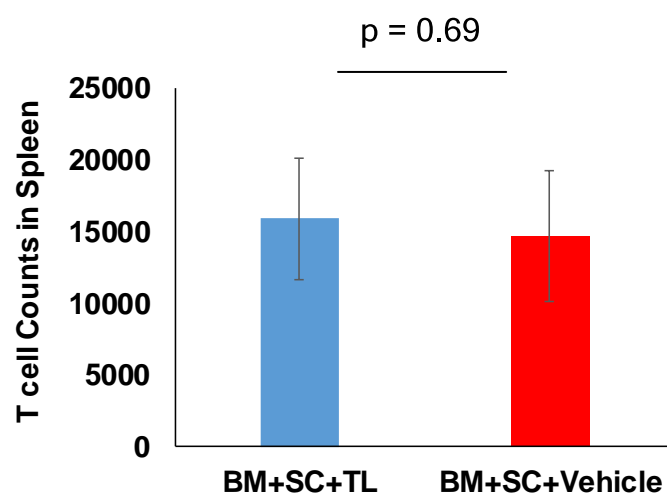

Supplement: S8 Fig — (a) Flow cytometry was carried out to count T cells in the spleen collected from TL- and vehicle-treated BM+SC recipient mice 28 days after BMT. (BM+SC+TL: blue, BM+SC+Vehicle: red) Data from one of two similar experiments are shown. Unpaired Student’s t-test was used to determine the statistical significance between the 2 groups of interest. The data are presented as means ± SD, BM+SC+TL: n = 5, BM+SC+Vehicle: n = 5 (PDF) [file pone.0203742.s009.pdf]

**S9 Fig.**

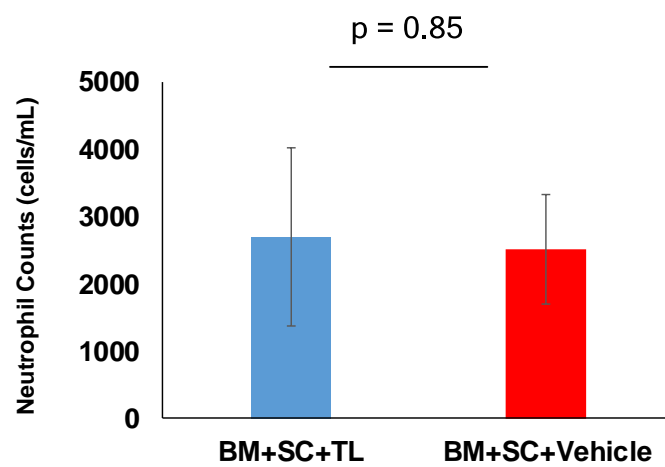

Supplement: S9 Fig — (a) Flow cytometry was carried out to count neutrophils in peripheral blood collected from TL- and vehicle-treated BM+SC recipient mice 21 days after BMT. (BM+SC+TL: blue, BM+SC+Vehicle: red) Data from one of two similar experiments are shown. Unpaired Student’s t-test was used to determine the statistical significance between the 2 groups of interest. The data are presented as means ± SD, BM+SC+TL: n = 4, BM+SC+Vehicle: n = 4 (PDF) [file pone.0203742.s010.pdf]

**S10 Fig.**

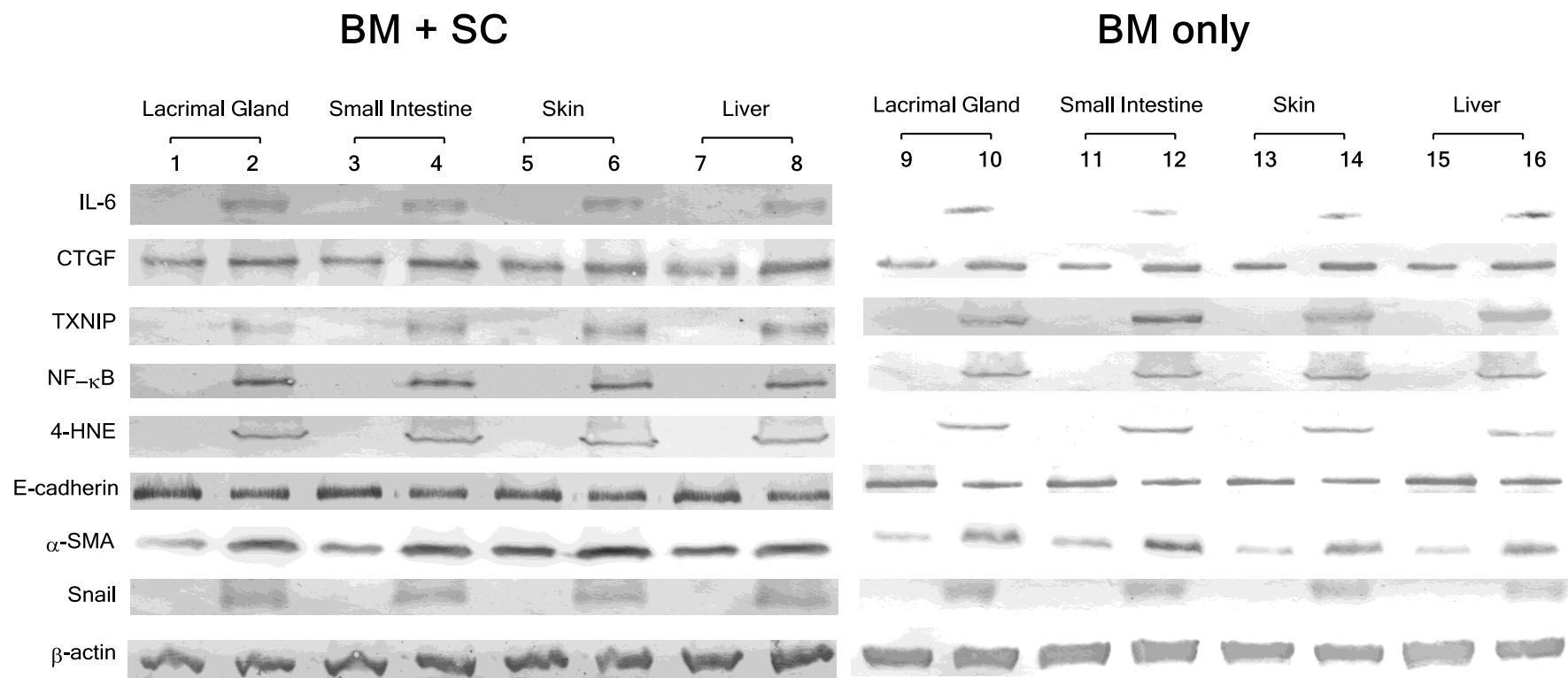

Supplement: S10 Fig — Immunoblot assays for IL-6, CTGF, TXNIP, NF-κB, 4-HNE, E-cadherin, α-SMA, Snail and β-actin in the individual samples was conducted. (BM+SC Lanes 1, 3, 5, 7: TL-treated organs, Lanes 2, 4, 6, 8: vehicle-treated organs, BM only Lanes 9, 11, 13, 15: TL-treated organs, Lanes 10, 12, 14, 16: vehicle-treated organs). (PDF) [file pone.0203742.s011.pdf]

S11 Fig.

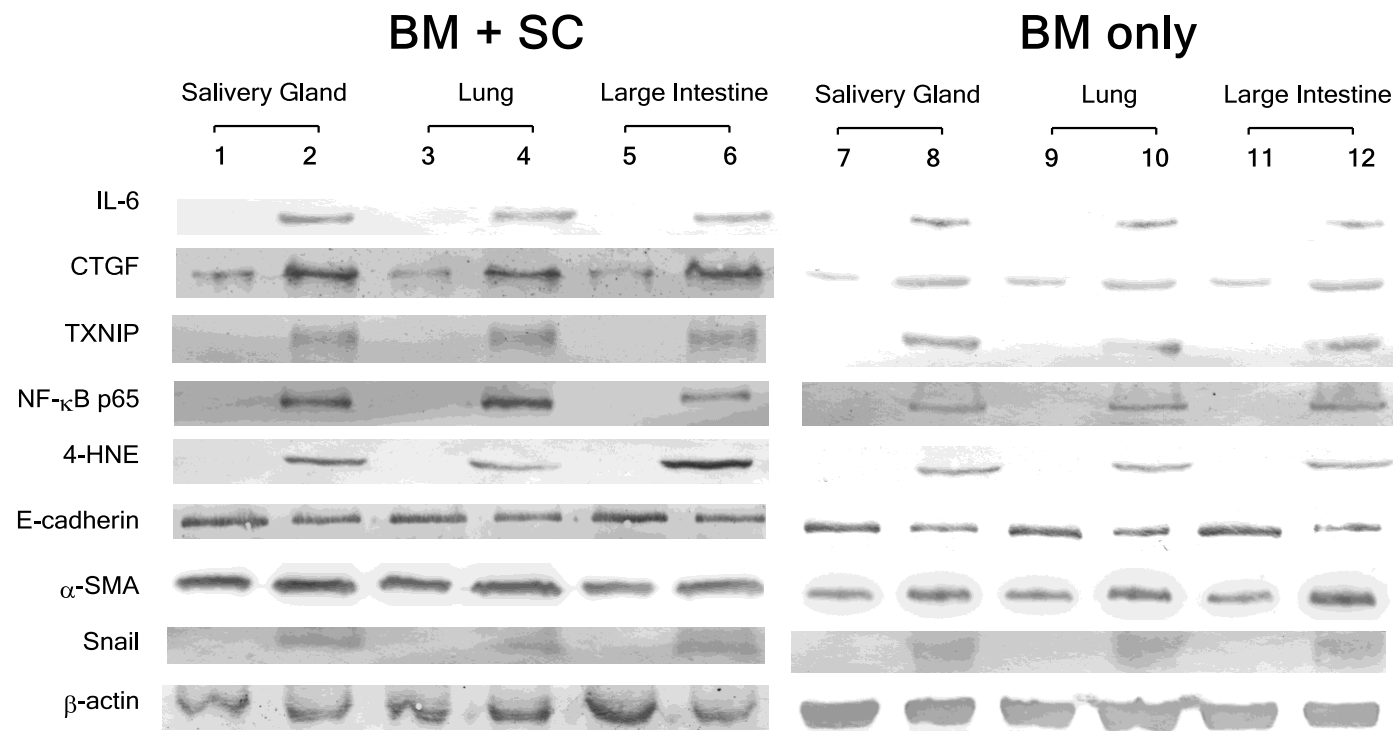

Supplement: S11 Fig — Immunoblot assays for IL-6, CTGF, TXNIP, NF-κB, 4-HNE, E-cadherin, α-SMA, Snail and β-actin in the individual samples was conducted. (BM+SC Lanes 1, 3, 5: TL-treated organs, Lanes 2, 4, 6: vehicle-treated organs, BM only Lanes 7, 9, 11: TL-treated organs, Lanes 8, 10, 12: vehicle-treated organs). (PDF) [file pone.0203742.s012.pdf]

**S12 Fig.**

**A**

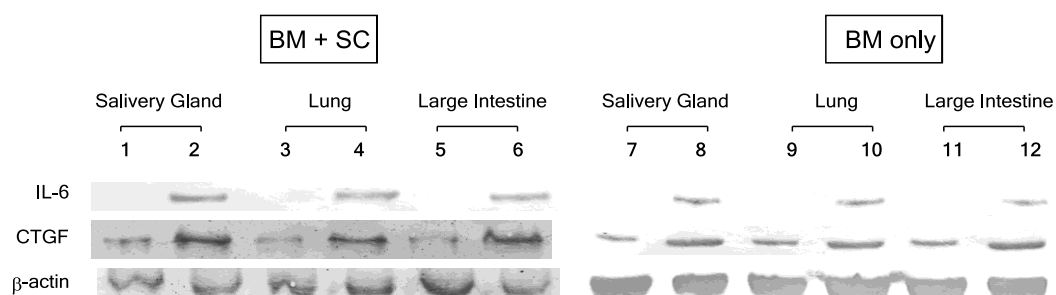

**B**

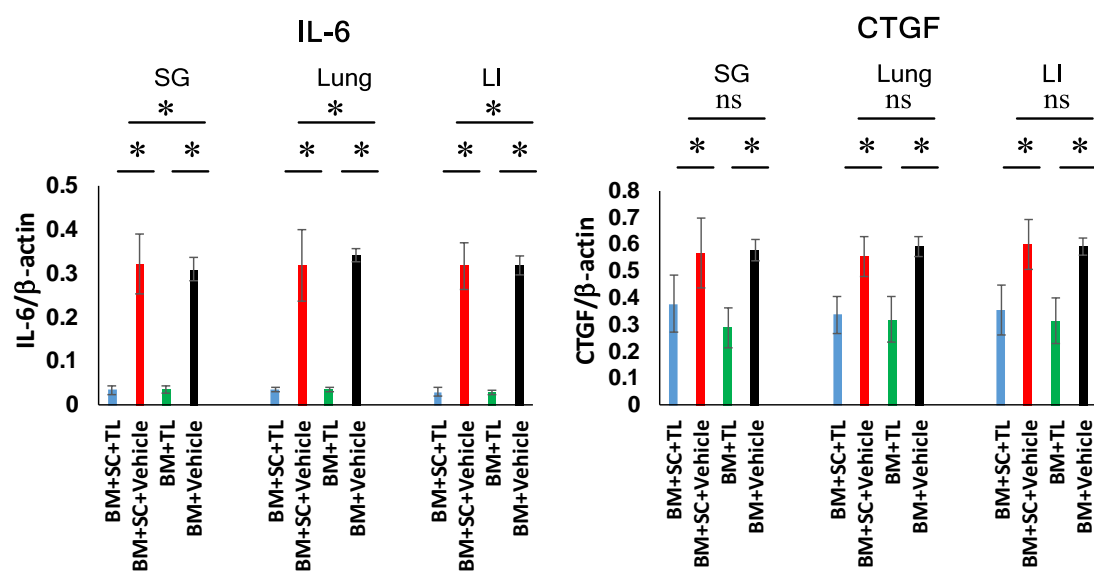

Supplement: S12 Fig — (A) Immunoblot assays for the inflammatory marker IL-6 and the fibrotic marker CTGF was conducted. (BM+SC Lanes 1, 3, 5: TL-treated organs, Lanes 2, 4, 6: vehicle-treated organs, BM only Lanes 7, 9, 11: TL-treated organs, Lanes 8, 10, 12: vehicle-treated organs) Note: A series of immunoblots is shown in S11 Fig, and blots of IL-6, CTGF and β-actin are taken from S11 Fig. (C) IL-6 and CTGF in each organ were subsequently quantified by densitometry. TL-injected organs (BM+SC+TL: blue, BM+TL: green) and their vehicle-injected partners (BM+SC+Vehicle: red, BM+Vehicle: black). Data from one of two similar experiments are shown. Unpaired Student’s t-test was used to determine the statistical significance between the 2 groups of interest. One-way ANOVA was utilized for the 4 groups of interest. The data are presented as means ± SD. BM+SC+TL: n = 8, BM+SC+Vehicle: n = 8, BM+TL: n = 6, BM+Vehicle: n = 6. *P<0.05. (PDF) [file pone.0203742.s013.pdf]

**S13 Fig.**

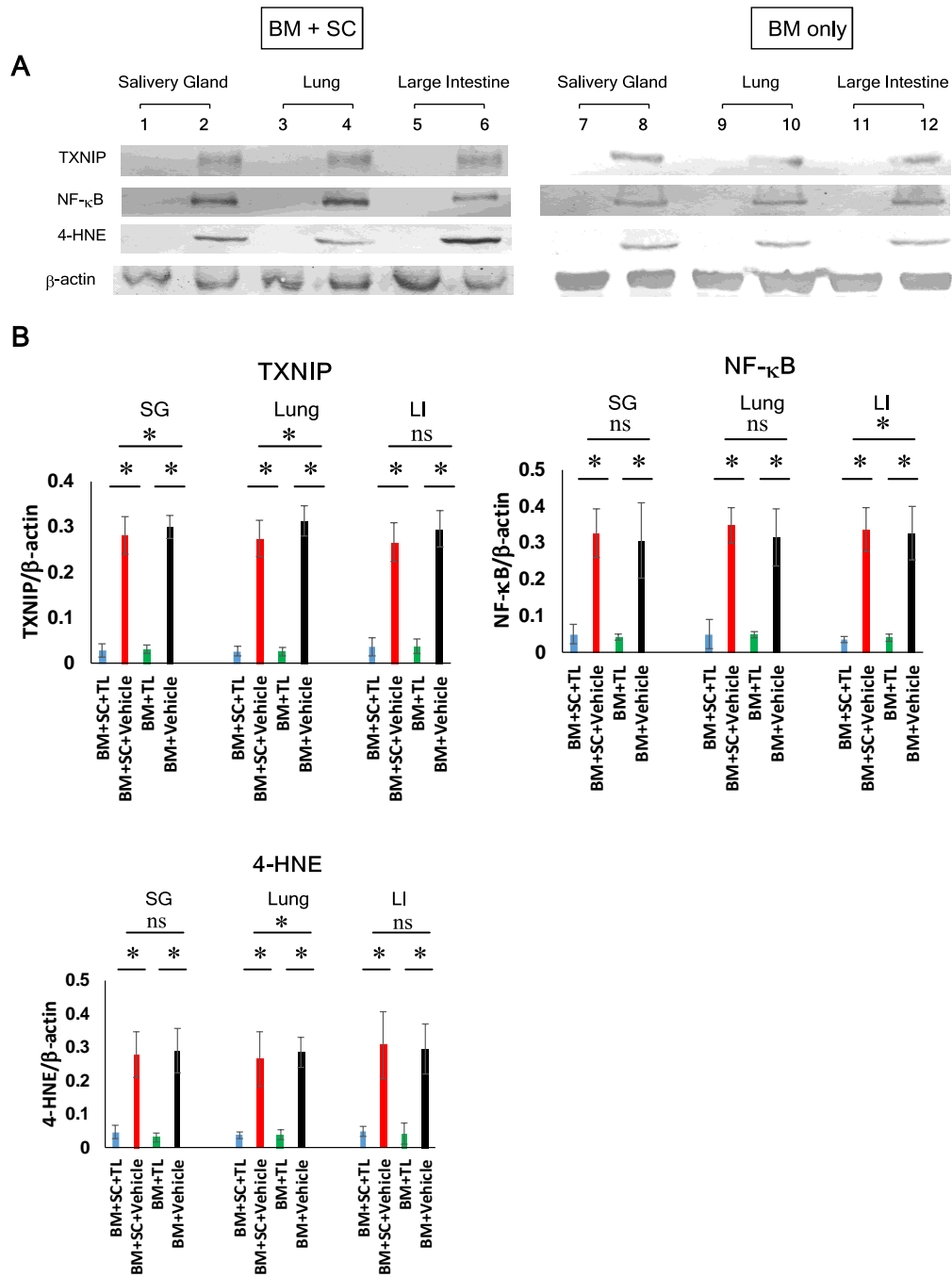

Supplement: S13 Fig — (A) Immunoblot investigation into TXNIP, NF-κB and 4-HNE was conducted. (BM+SC Lanes 1, 3, 5: TL-treated organs, Lanes 2, 4, 6: vehicle-treated organs, BM only Lanes 7, 9, 11: TL-treated organs, Lanes 8, 10, 12: vehicle-treated organs) Note: A series of immunoblots is shown in S11 Fig, and blots of TXNIP, NF-κB, 4-HNE and β-actin are taken from S11 Fig. (C) TXNIP, NF-κB, 4-HNE in each organ were subsequently quantified by densitometry. TL-injected organs (BM+SC+TL: blue, BM+TL: green) and their vehicle-injected partners (BM+SC+Vehicle: red, BM+Vehicle: black). Data from one of two similar experiments are shown. Unpaired Student’s t-test was used to determine the statistical significance between the 2 groups of interest. One-way ANOVA was utilized for the 4 groups of interest. The data are presented as means ± SD. BM+SC+TL: n = 8, BM+SC+Vehicle: n = 8, BM+TL: n = 6, BM+Vehicle: n = 6. *P<0.05. (PDF) [file pone.0203742.s014.pdf]

**S14 Fig.**

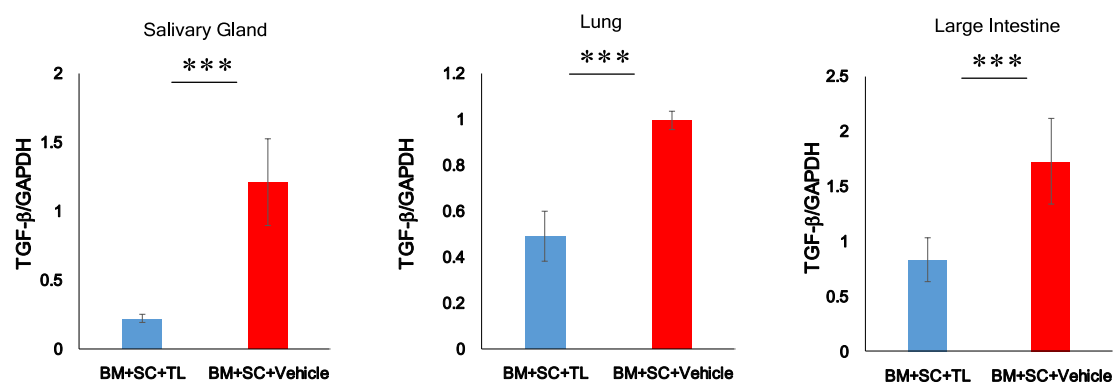

Supplement: S14 Fig — qPCR for TGF-β in TL-medicated organs (BM+SC+TL: blue) and their vehicle-medicated counterparts (BM+SC+Vehicle: red). Data from one of two similar experiments are shown. Unpaired Student’s t-test was used to determine the statistical significance between the 2 groups of interest. The data are presented as means ± SD. BM+SC+TL: n = 4–6, BM+SC+Vehicle n = 5–6. *P<0.05, **P<0.01, ***P<0.001. (PDF) [file pone.0203742.s015.pdf]

**S15 Fig.**

**A**

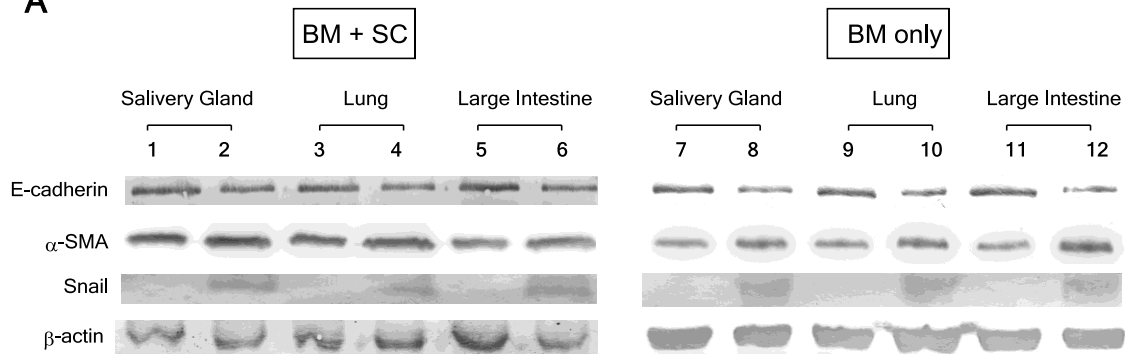

**B**

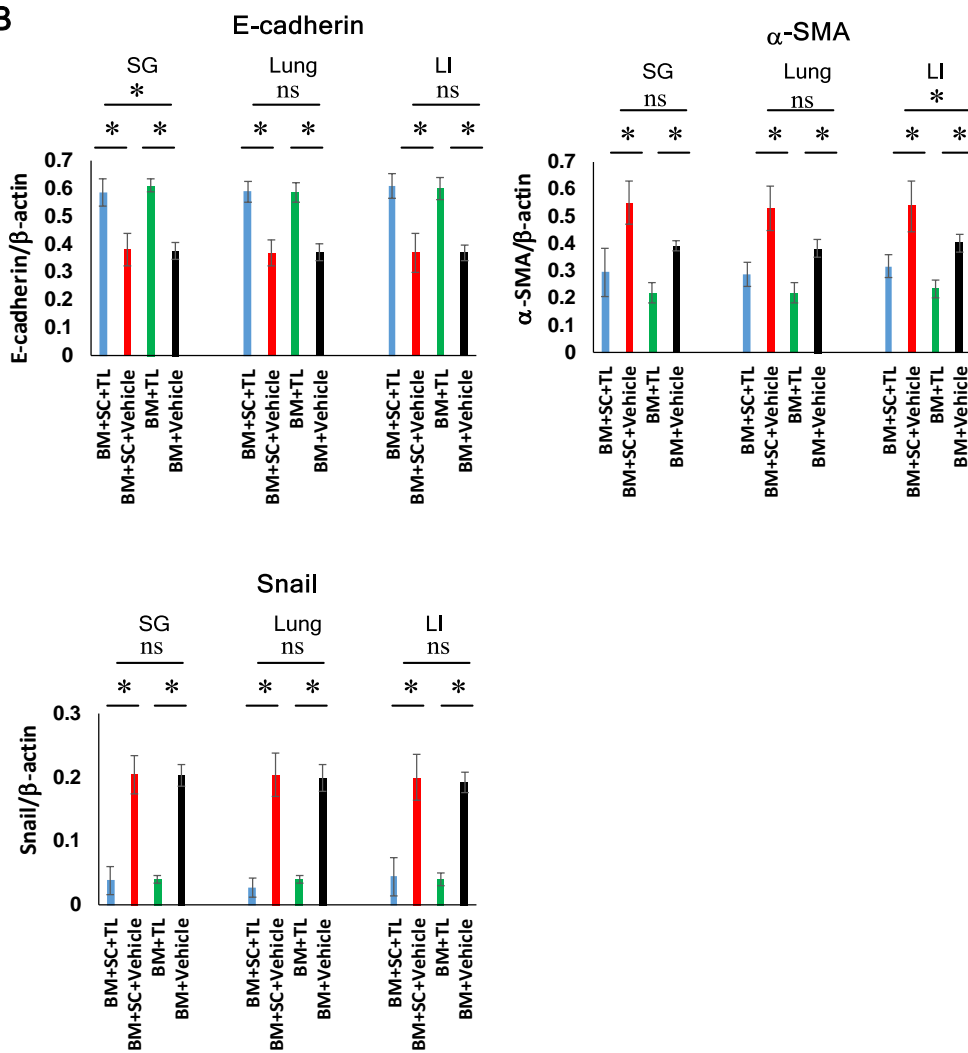

Supplement: S15 Fig — (A) Immunoblot analysis of E-cadherin, α-SMA and Snail was performed. (BM+SC Lanes 1, 3, 5: TL-treated organs, Lanes 2, 4, 6: vehicle-treated organs, BM only Lanes 7, 9, 11: TL-treated organs, Lanes 8, 10, 12: vehicle-treated organs) Note: A series of immunoblots is shown in S11 Fig, and blots of E-cadherin, α-SMA, Snail and β-actin are taken from S11 Fig. (C) TXNIP, NF-κB, 4-HNE in each organ were subsequently quantified by densitometry. TL-injected organs (BM+SC+TL: blue, BM+TL: green) and their vehicle-injected partners (BM+SC+Vehicle: red, BM+Vehicle: black). Data from one of two similar experiments are shown. Unpaired Student’s t-test was used to determine the statistical significance between the 2 groups of interest. One-way ANOVA was utilized for the 4 groups of interest. The data are presented as means ± SD. BM+SC+TL: n = 8, BM+SC+Vehicle: n = 8, BM+TL: n = 6, BM+Vehicle: n = 6. *P<0.05. (PDF) [file pone.0203742.s016.pdf]

**S16 Fig.**

**BM + SC**

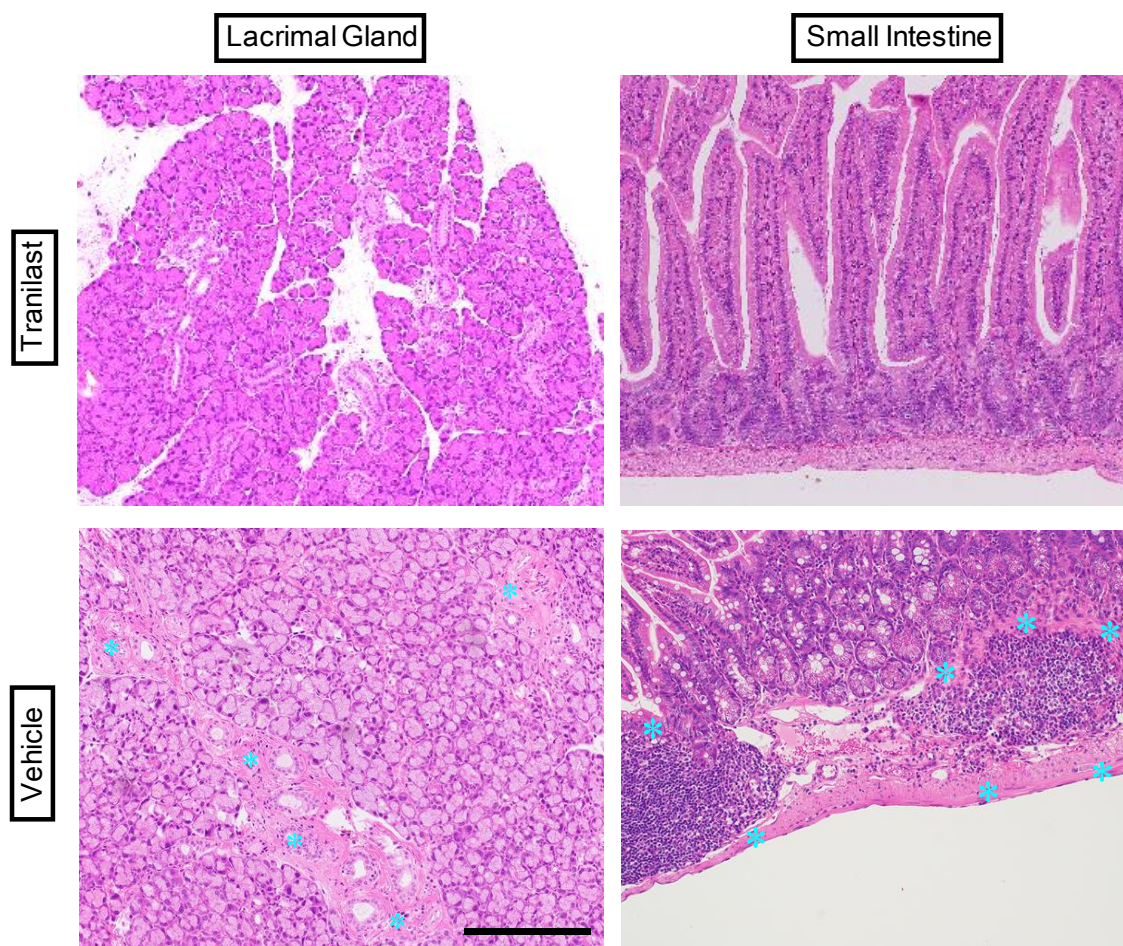

Supplement: S16 Fig — The images were taken at 200x magnification, and the scale bar is 200 μm. Severely inflamed portions are shown with blue asterisks. (PDF) [file pone.0203742.s017.pdf]

**S17 Fig.**

**BM + SC**

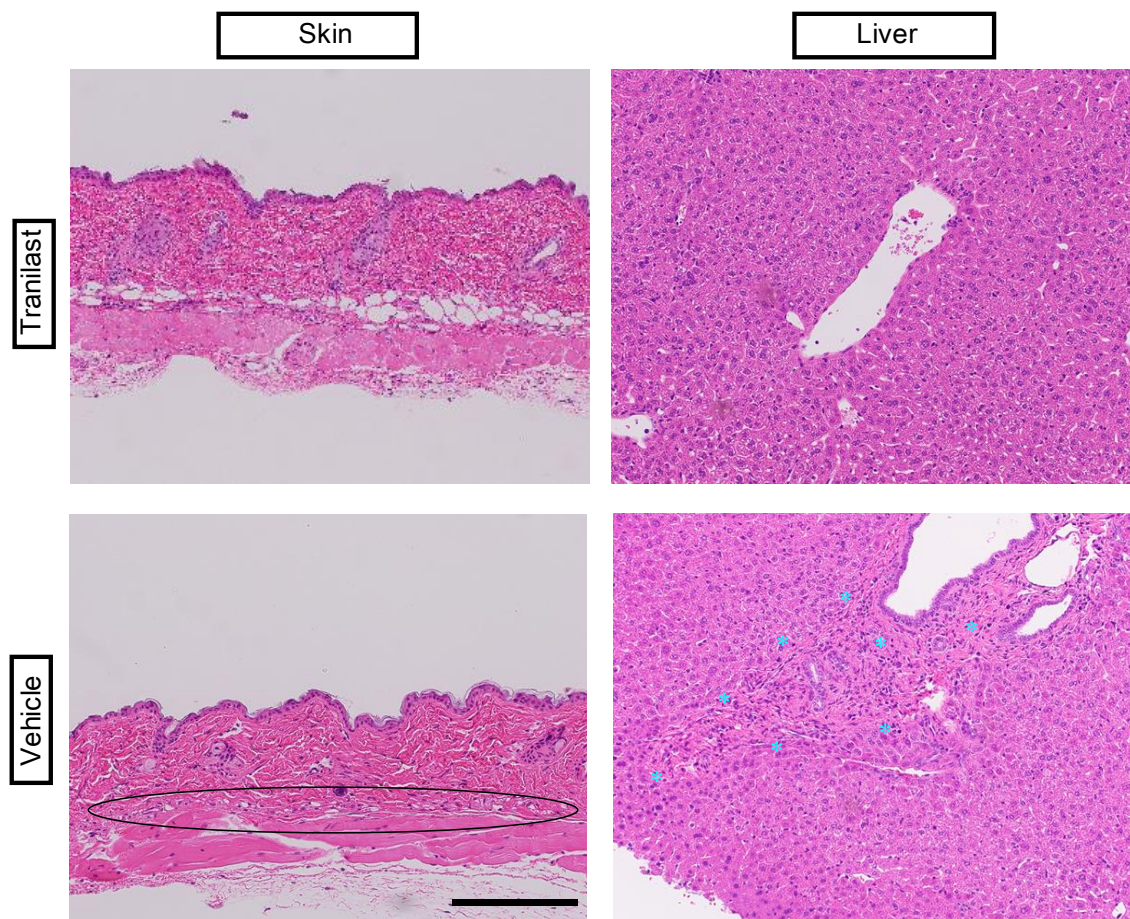

Supplement: S17 Fig — The images were taken at 200x magnification, and the scale bar is 200 μm. Severely inflamed portions are shown with blue asterisks. In the picture of the vehicle-medicated skin, an ellipse is placed where the fatty tissues were lost. (PDF) [file pone.0203742.s018.pdf]

**S18 Fig.**

**BM + SC**

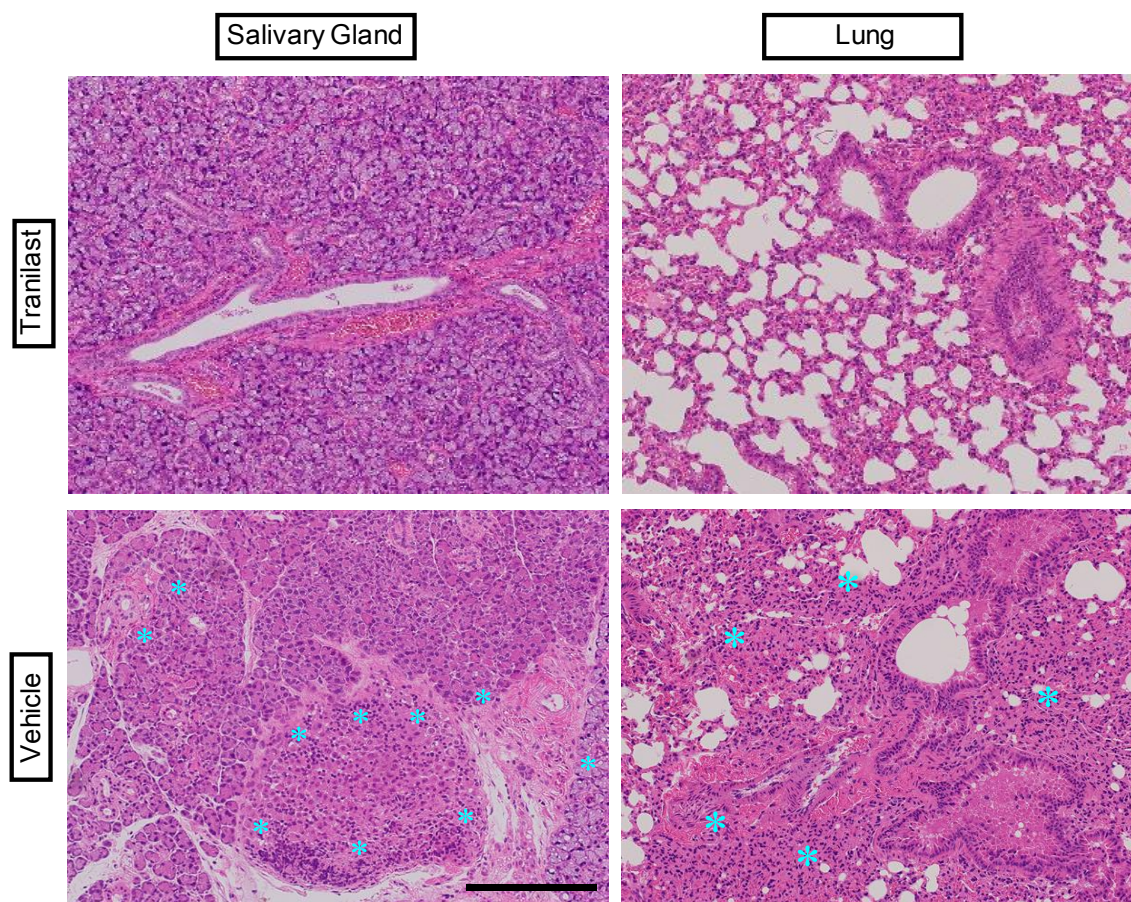

Supplement: S18 Fig — The images were taken at 200x magnification, and the scale bar is 200 μm. Severely inflamed portions are shown with blue asterisks. (PDF) [file pone.0203742.s019.pdf]

**S19 Fig.**

**BM + SC**

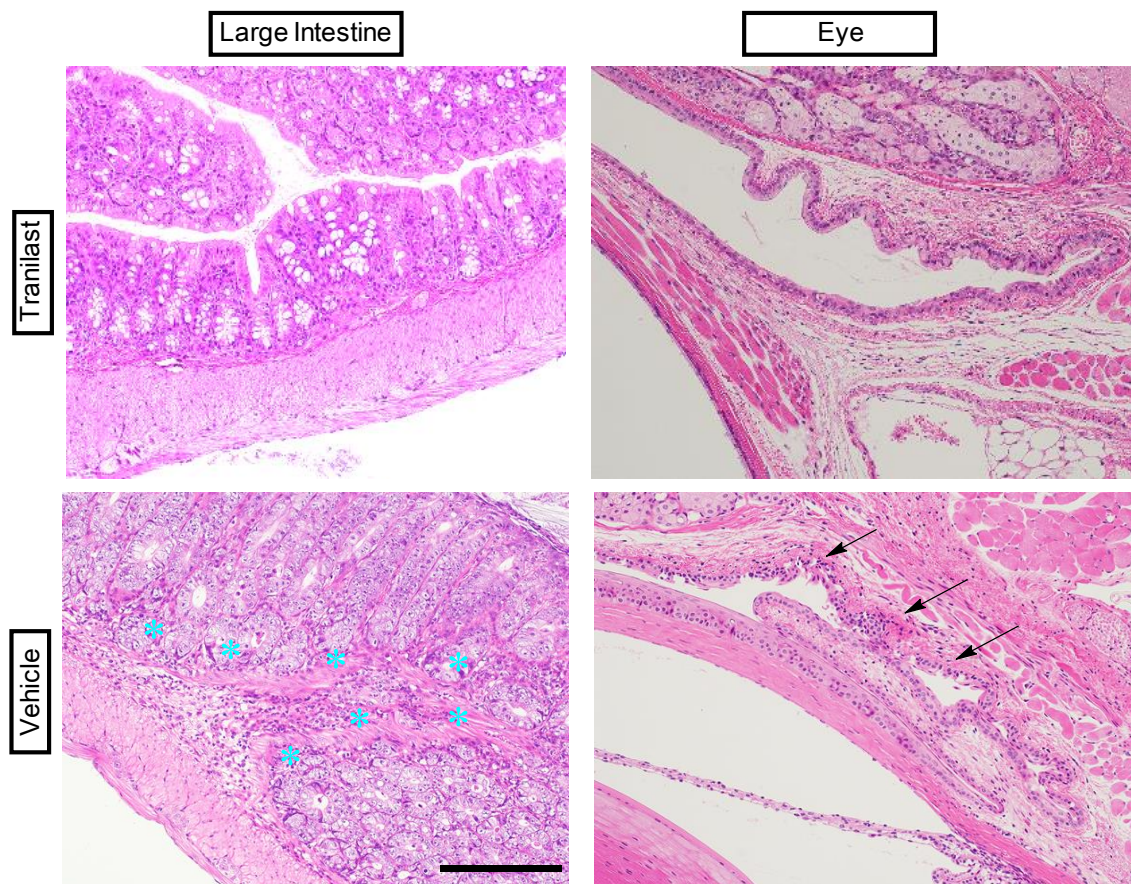

Supplement: S19 Fig — The images were taken at 200x magnification, and the scale bar is 200 μm. Severely inflamed portions are shown with blue asterisks. In the picture of the vehicle-medicated eye, arrows were placed where its conjunctiva was severely damaged. (PDF) [file pone.0203742.s020.pdf]

**S20 Fig.**

**BM + SC**

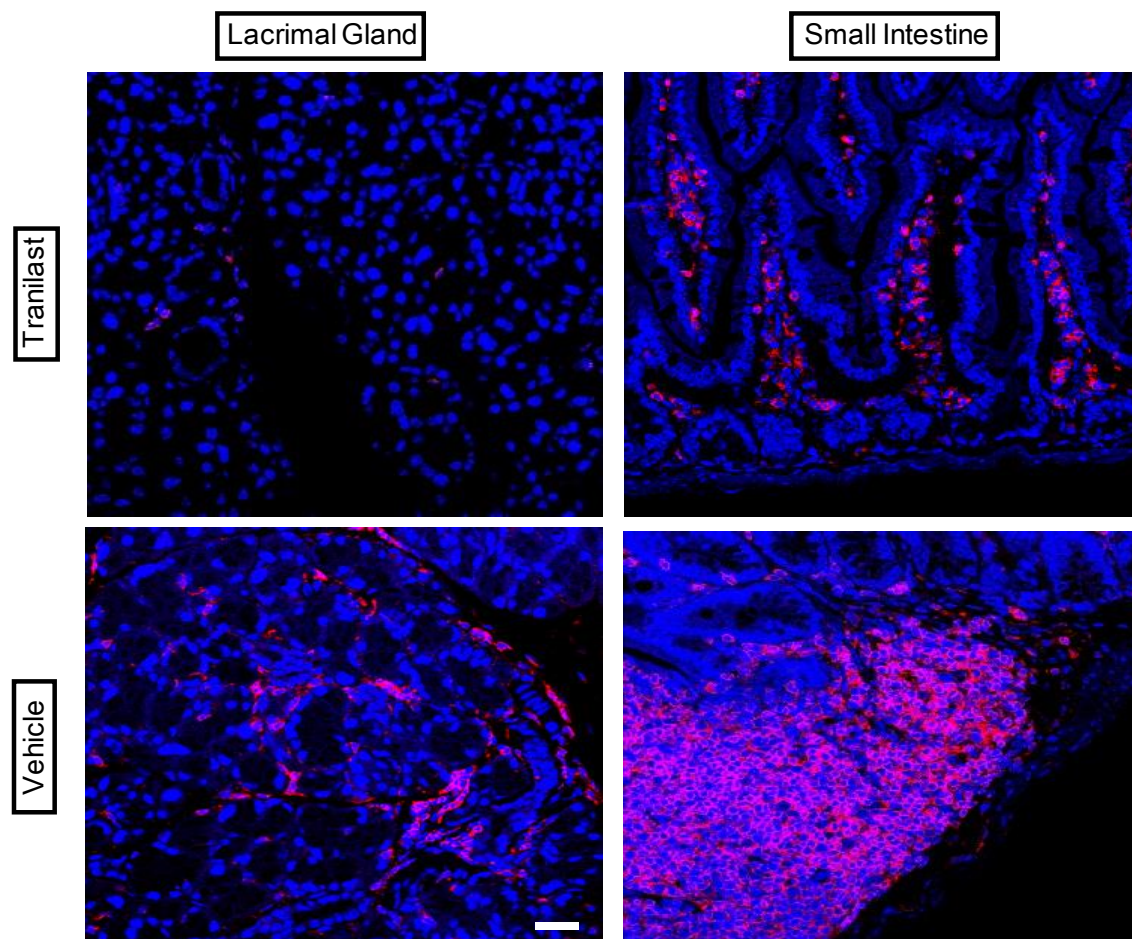

Supplement: S20 Fig — CD45 and cell nuclei are stained red and blue, respectively. The images were taken at 200x magnification, and the scale bar is 20 μm. (PDF) [file pone.0203742.s021.pdf]

**S21 Fig.**

**BM + SC**

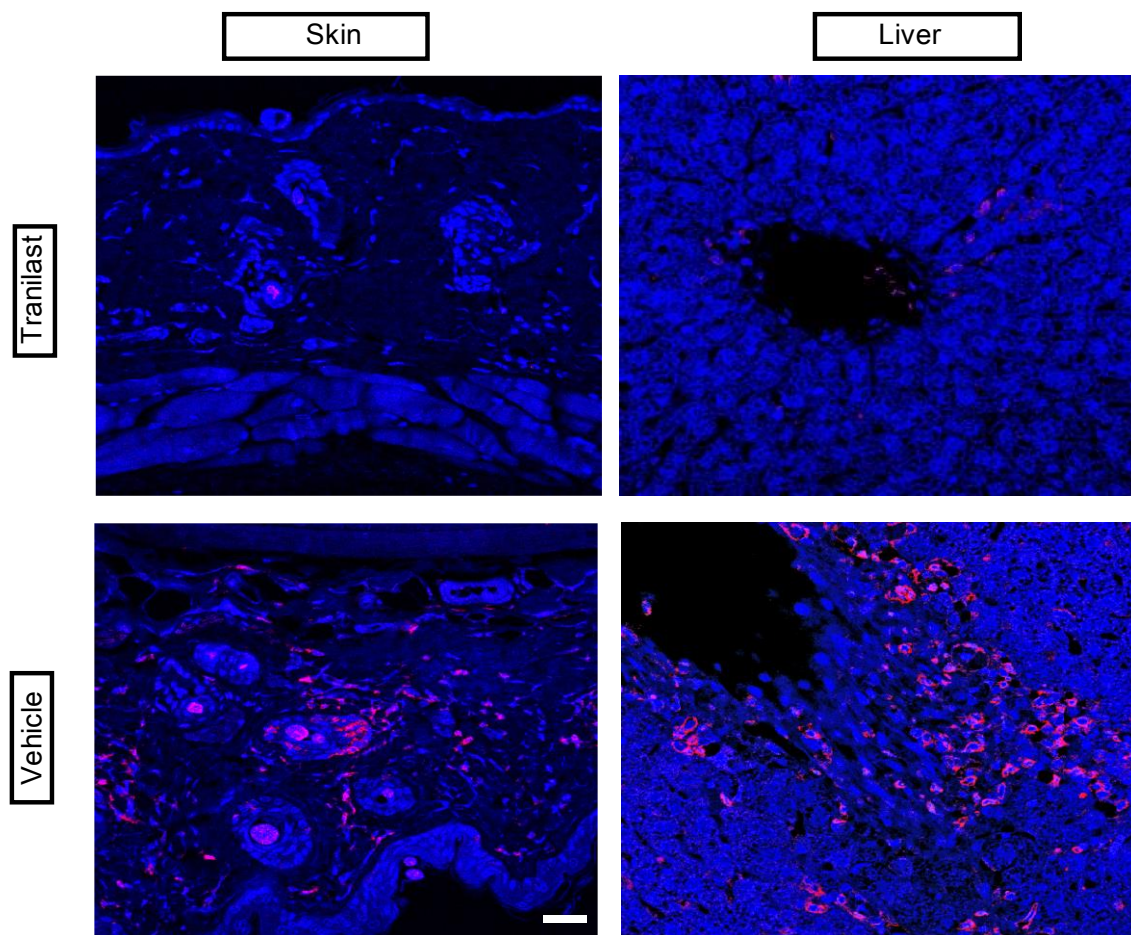

Supplement: S21 Fig — CD45 and cell nuclei are stained red and blue, respectively. The images were taken at 200x magnification, and the scale bar is 20 μm. (PDF) [file pone.0203742.s022.pdf]

**S22 Fig.**

**BM + SC**

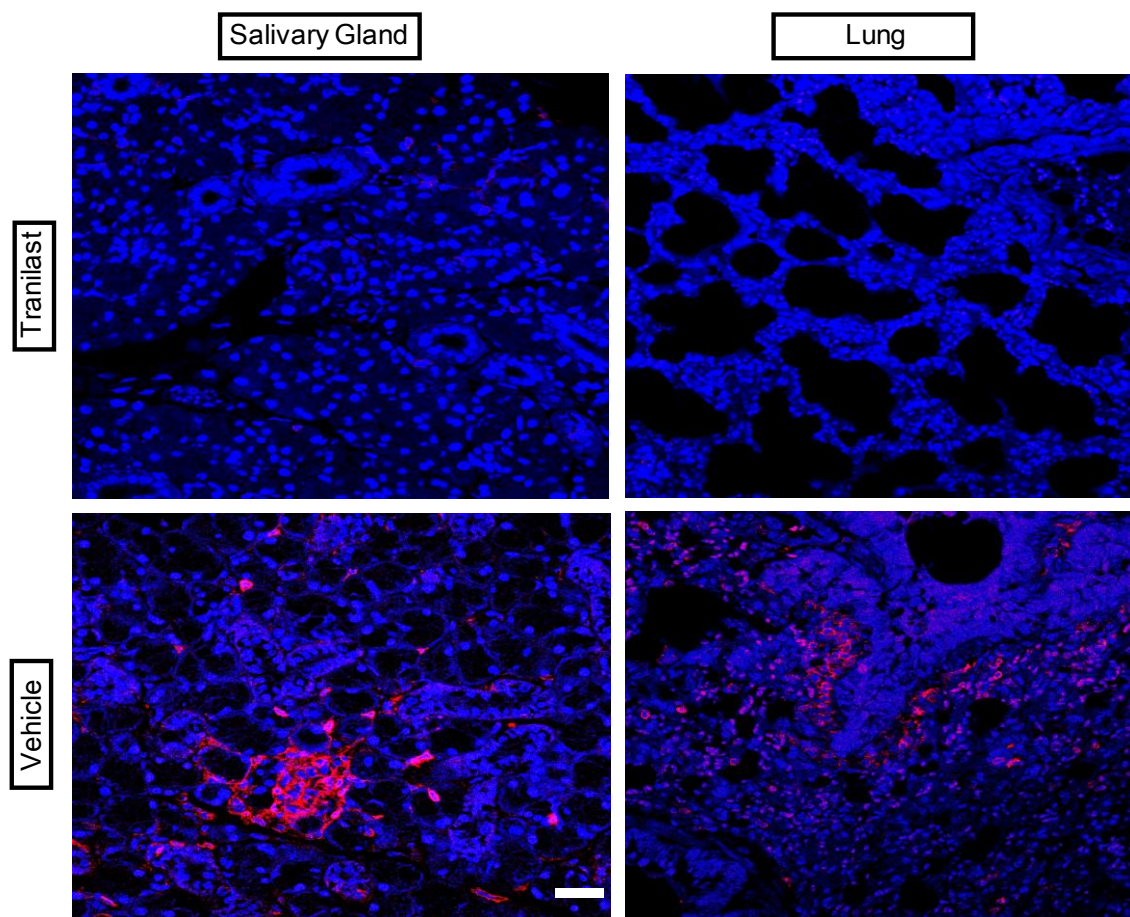

Supplement: S22 Fig — CD45 and cell nuclei are stained red and blue, respectively. The images were taken at 200x magnification, and the scale bar is 20 μm. (PDF) [file pone.0203742.s023.pdf]

S23 Fig.

BM + SC

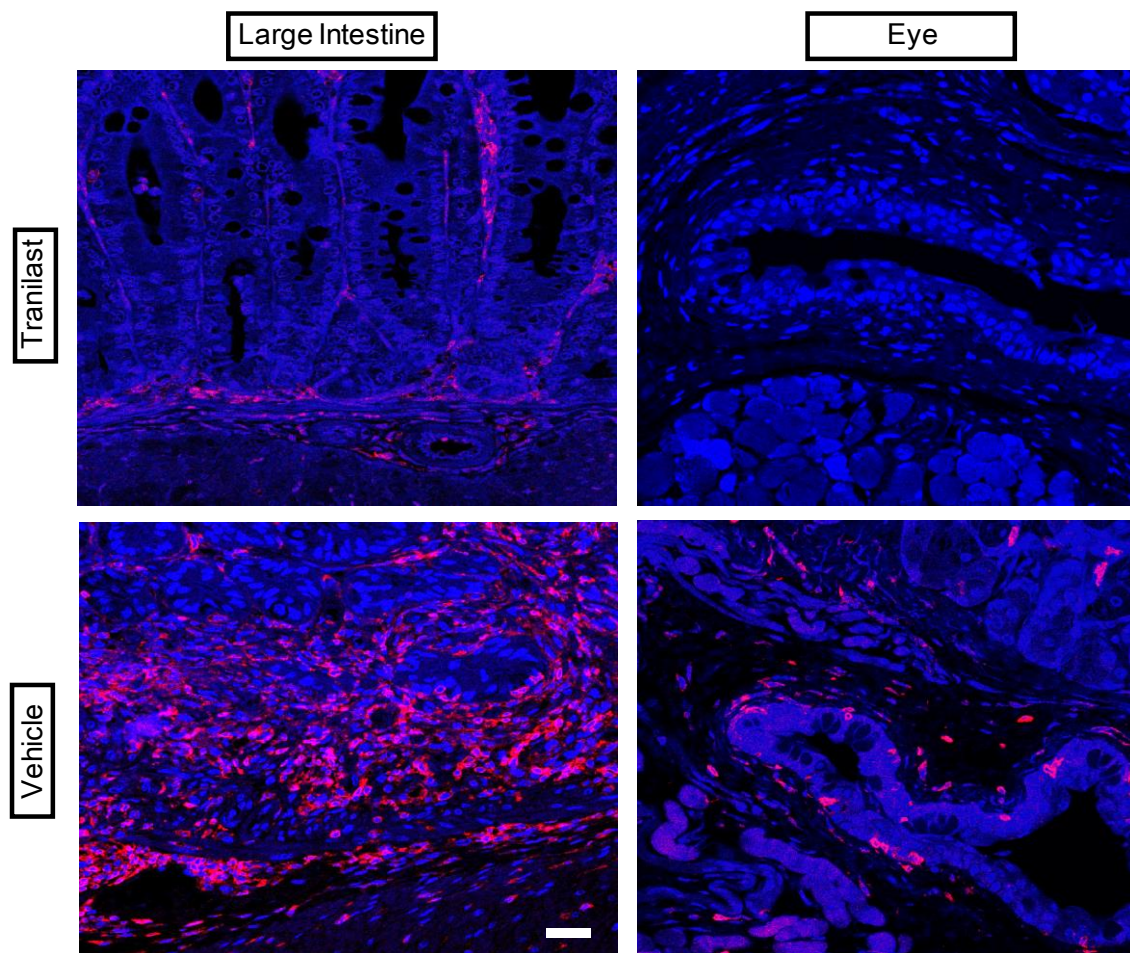

Supplement: S23 Fig — CD45 and cell nuclei are stained red and blue, respectively. The images were taken at 200x magnification, and the scale bar is 20 μm. (PDF) [file pone.0203742.s024.pdf]

**S24 Fig.**

**BM + SC**

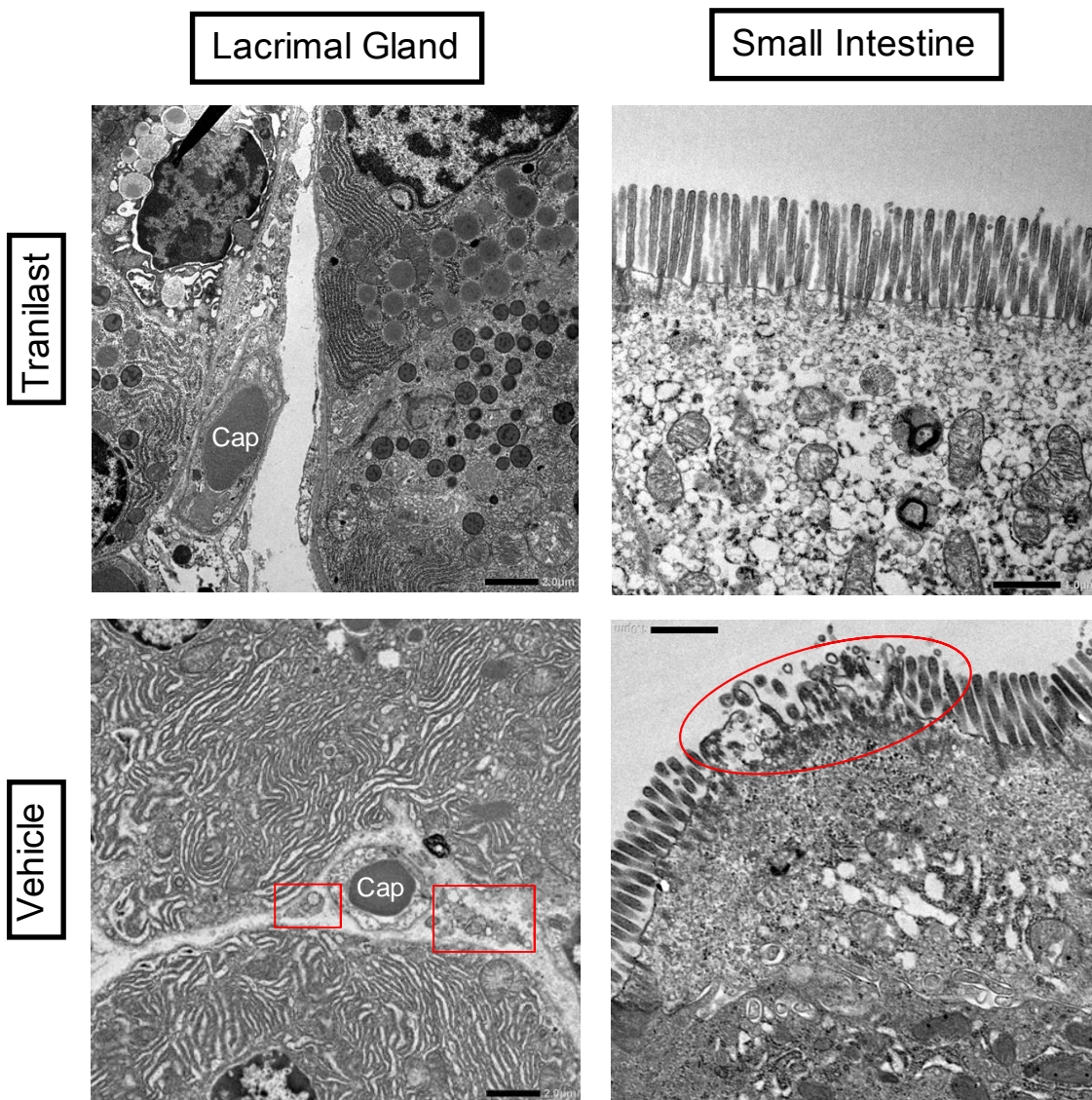

Supplement: S24 Fig — The pictures of stroma of the lacrimal glands (left) and epithelial cells of the small intestine (right) were taken at 2000x magnification and at 5000x magnification, respectively. The scale bar is 5 μm. Cap: Capillary. In the pictures of the vehicle-medicated lacrimal glands, cell debris is shown with a rectangle. In the photograph of the vehicle-injected small intestine, an ellipse is placed where microvilli were demolished. (PDF) [file pone.0203742.s025.pdf]

**S25 Fig.**

**BM + SC**

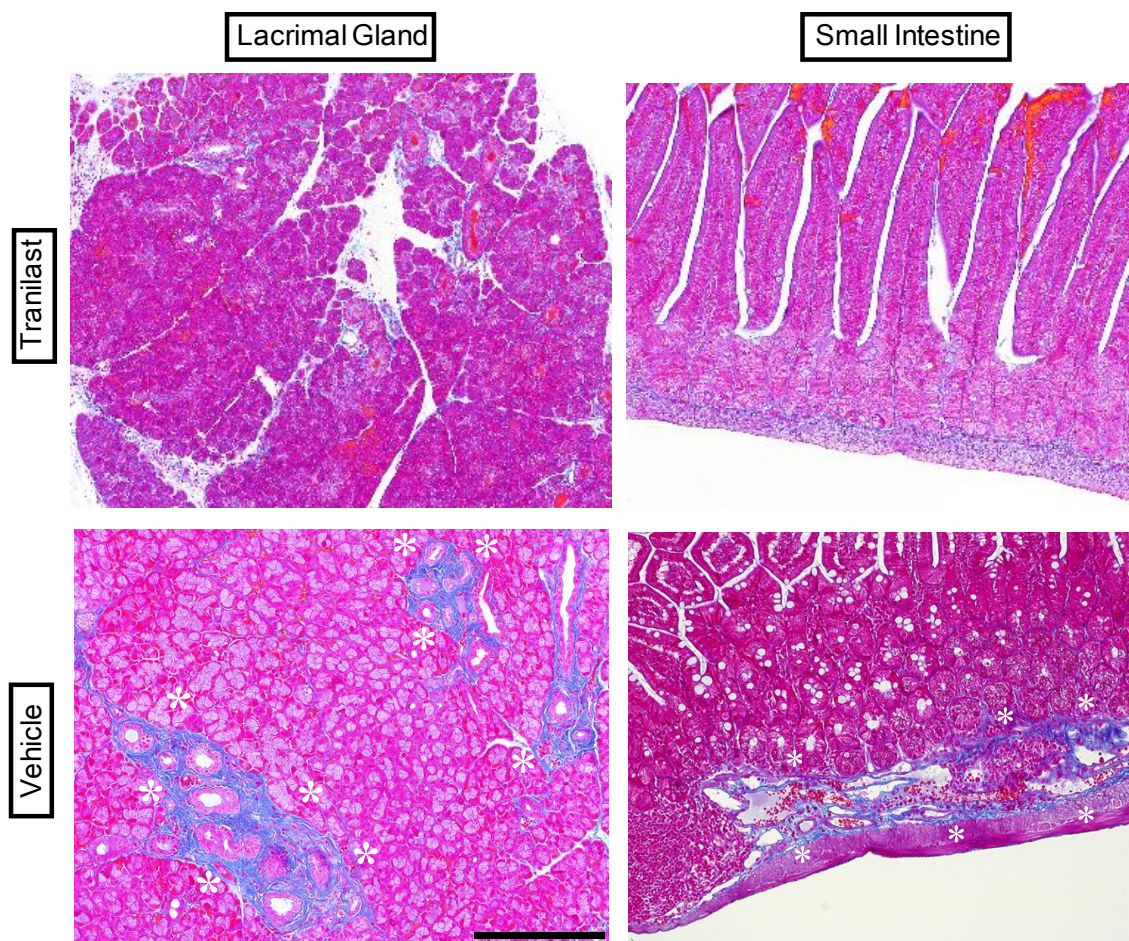

Supplement: S25 Fig — The pictures were taken at 200x magnification, and the scale bar is 200 μm. Excessively fibrotic areas are shown with white asterisks. (PDF) [file pone.0203742.s026.pdf]

**S26 Fig.**

**BM + SC**

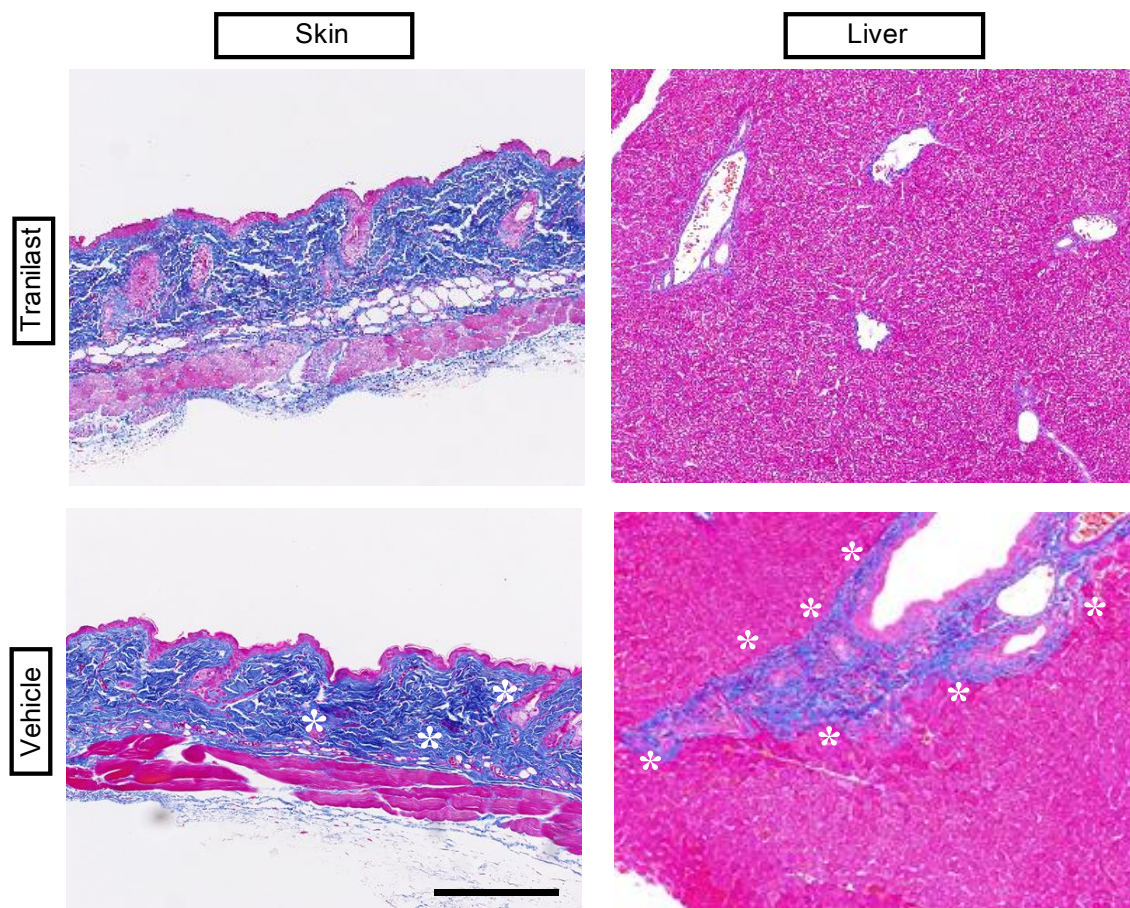

Supplement: S26 Fig — The pictures were taken at 200x magnification, and the scale bar is 200 μm. Excessively fibrotic areas are shown with white asterisks. (PDF) [file pone.0203742.s027.pdf]

**S27 Fig.**

**BM + SC**

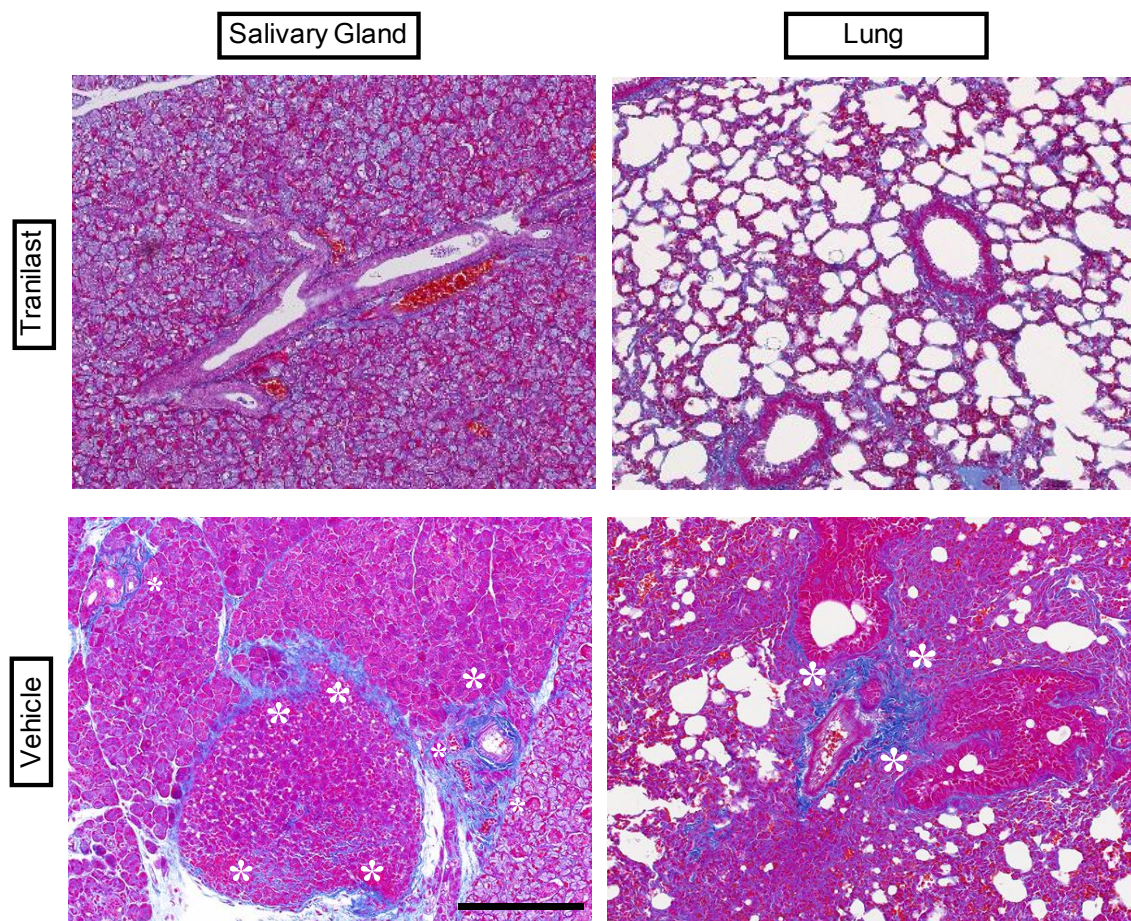

Supplement: S27 Fig — The pictures were taken at 200x magnification, and the scale bar is 200 μm. Excessively fibrotic areas are shown with white asterisks. (PDF) [file pone.0203742.s028.pdf]

**S28 Fig.**

**BM + SC**

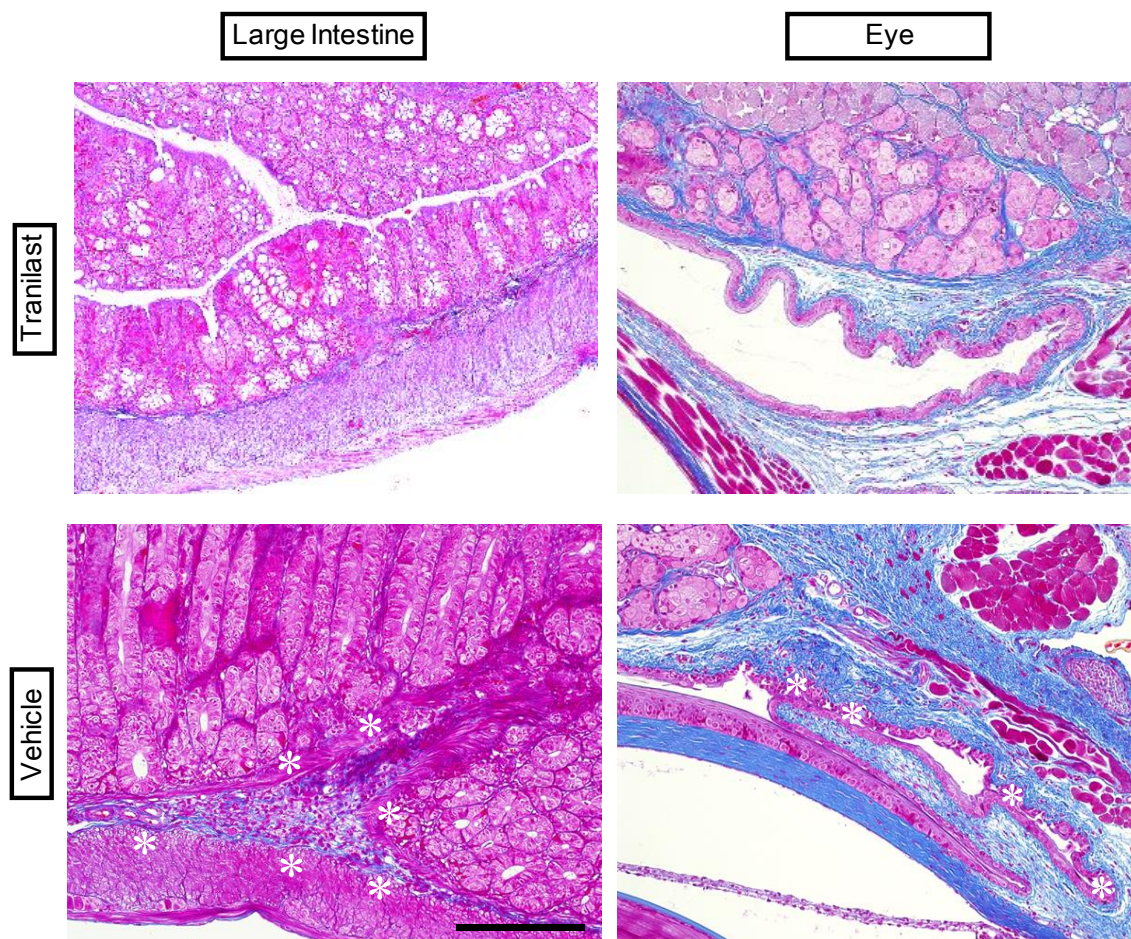

Supplement: S28 Fig — The pictures were taken at 200x magnification, and the scale bar is 200 μm. Excessively fibrotic areas are shown with white asterisks. (PDF) [file pone.0203742.s029.pdf]

**S29 Fig.**

**BM Only**

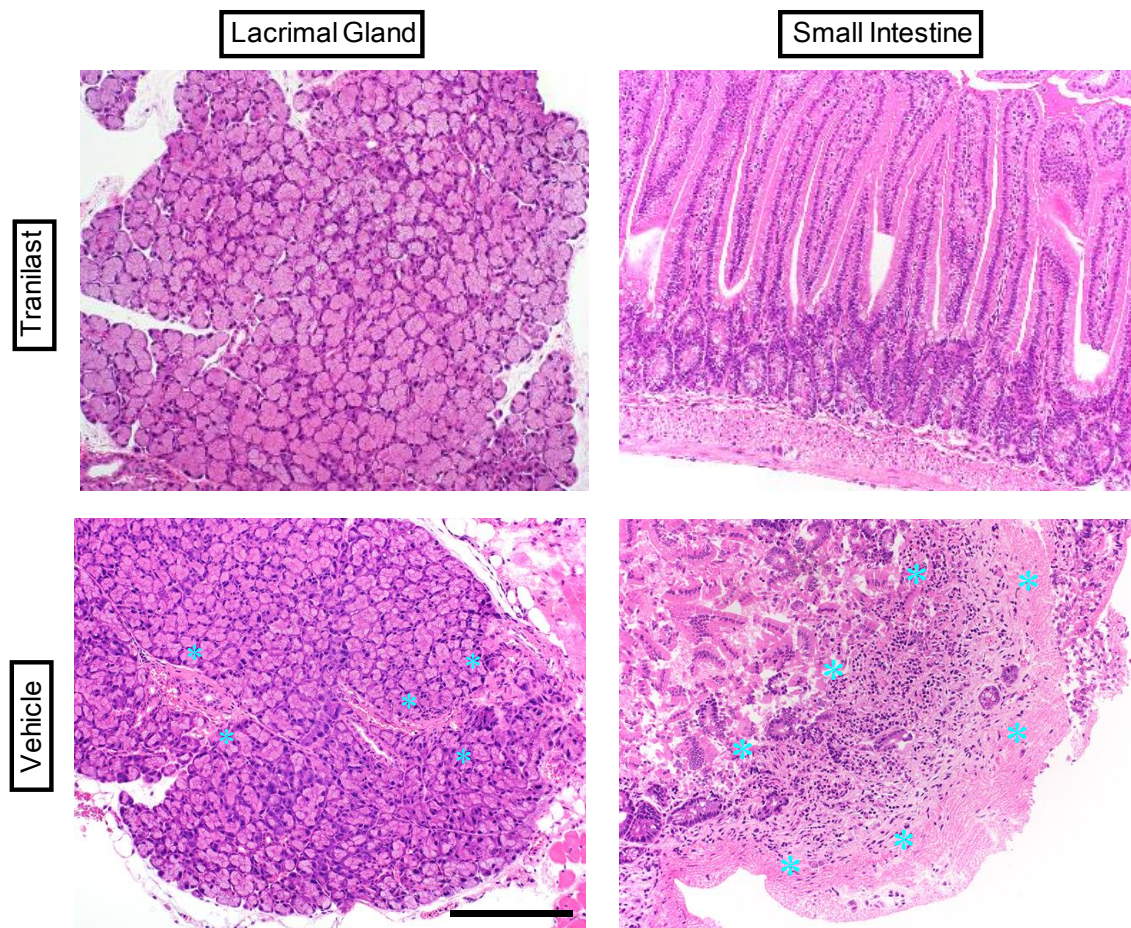

Supplement: S29 Fig — The images were taken at 200x magnification, and the scale bar is 200 μm. Severely inflamed portions are shown with blue asterisks. (PDF) [file pone.0203742.s030.pdf]

**S30 Fig.**

**BM Only**

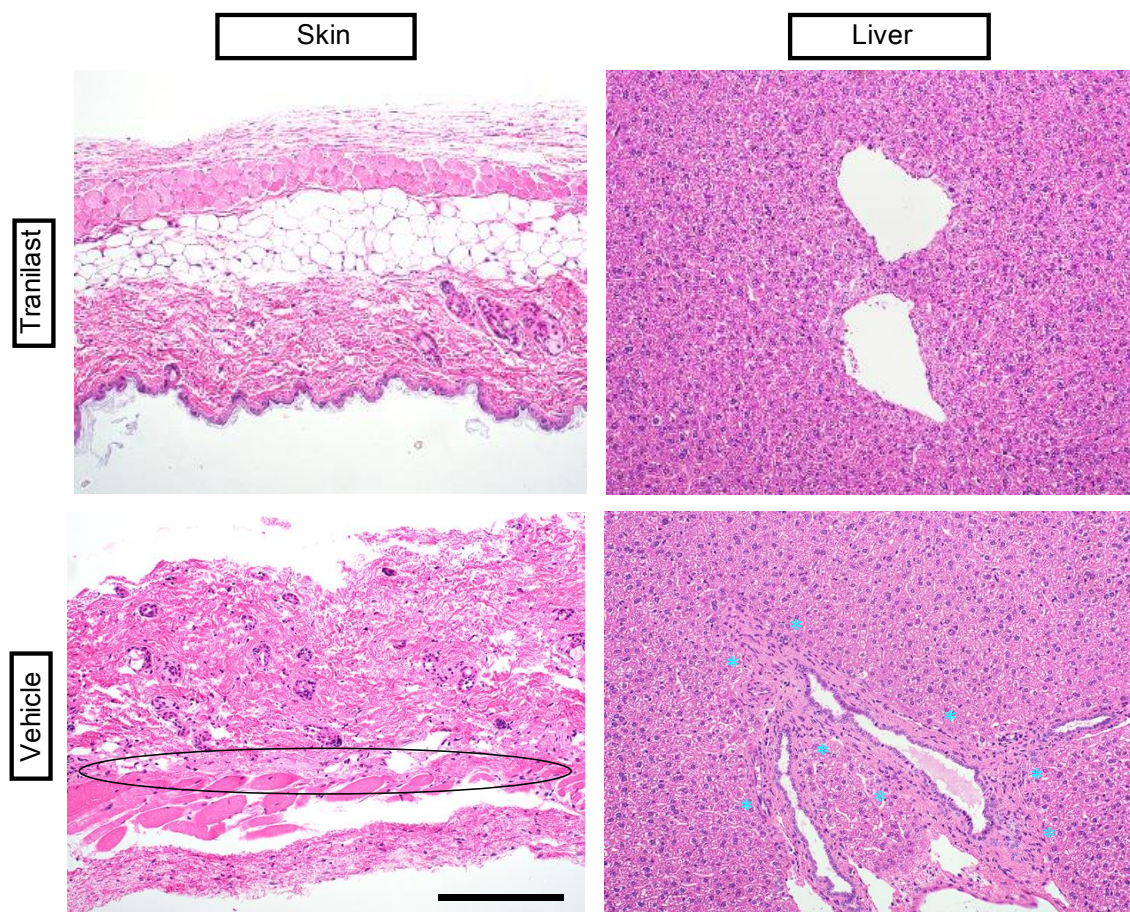

Supplement: S30 Fig — The images were taken at 200x magnification, and the scale bar is 200 μm. Severely inflamed portions are shown with blue asterisks. In the picture of the vehicle-medicated skin, loss of fatty tissues was indicated by an ellipse. (PDF) [file pone.0203742.s031.pdf]

**S31 Fig.**

**BM Only**

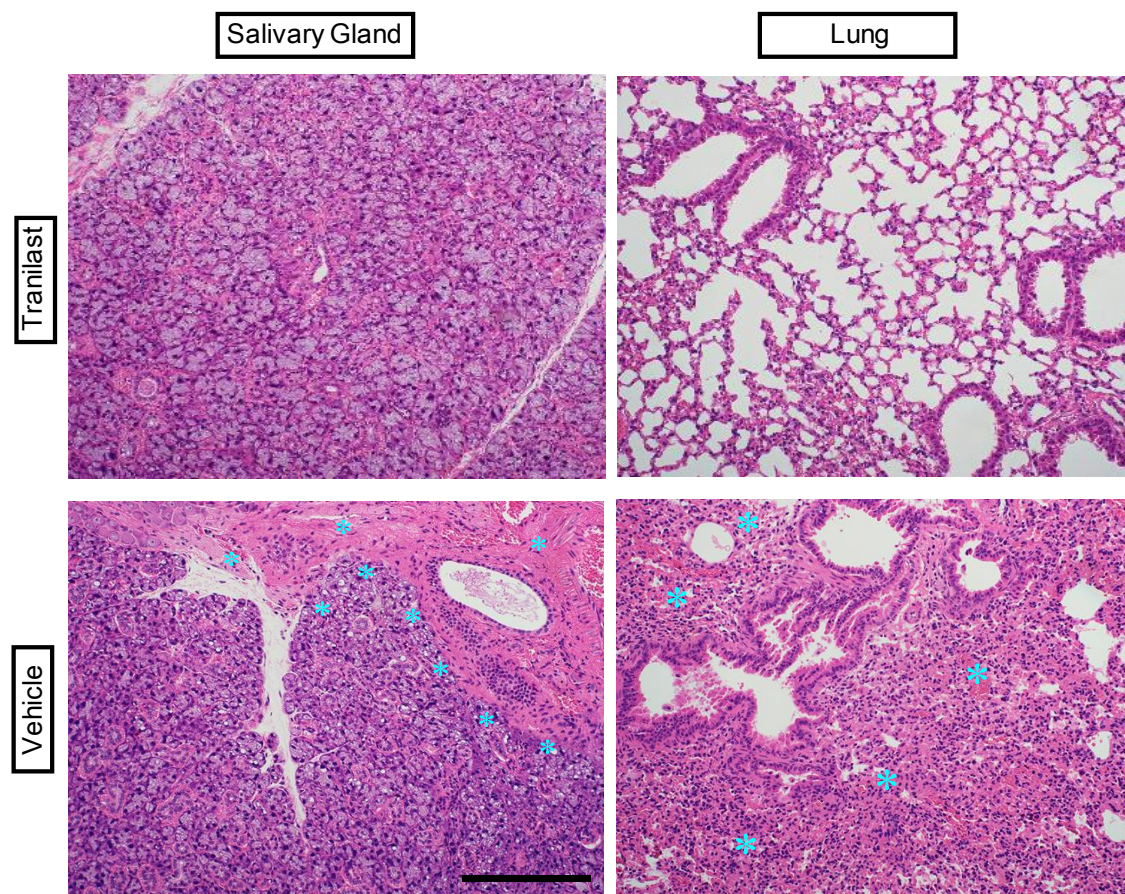

Supplement: S31 Fig — The images were taken at 200x magnification, and the scale bar is 200 μm. Severely inflamed portions are shown with blue asterisks. (PDF) [file pone.0203742.s032.pdf]

**S32 Fig.**

**BM Only**

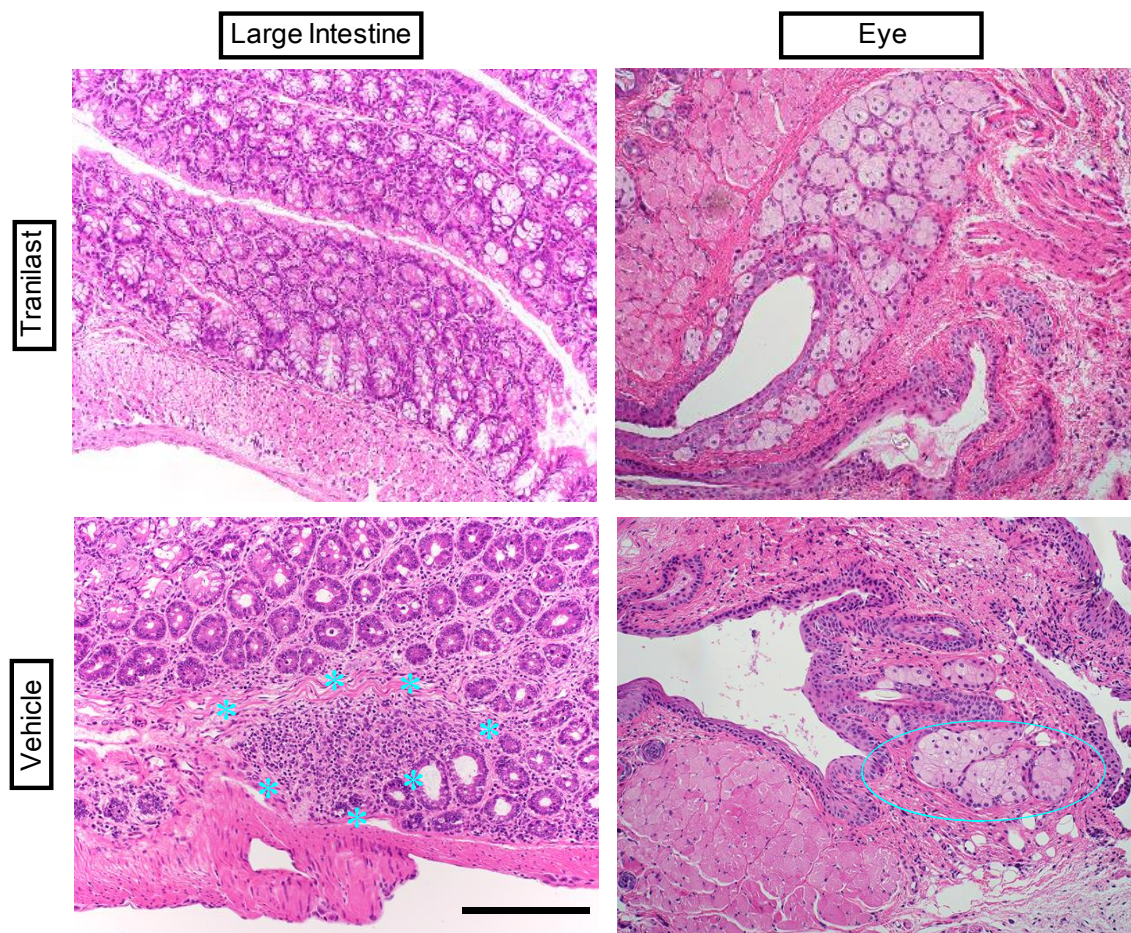

Supplement: S32 Fig — The images were taken at 200x magnification, and the scale bar is 200 μm. Severely inflamed portions are shown with blue asterisks. In the picture of the vehicle-medicated eye, the thinning and decrease of meibomian glands were indicated with a circle. (PDF) [file pone.0203742.s033.pdf]

**S33 Fig.**

**BM Only**

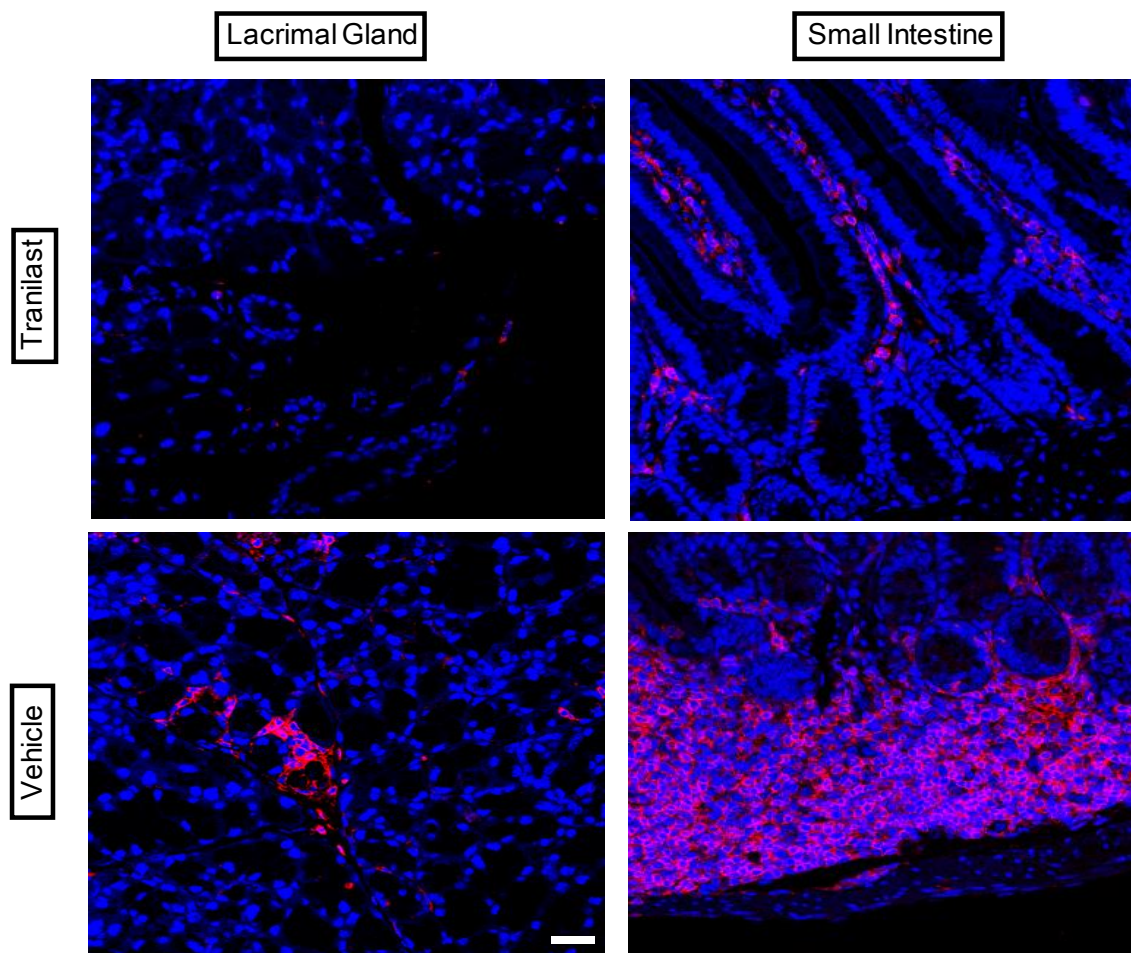

Supplement: S33 Fig — CD45 and cell nuclei are stained red and blue, respectively. The images were taken at 200x magnification, and the scale bar is 20 μm. (PDF) [file pone.0203742.s034.pdf]

**S34 Fig.**

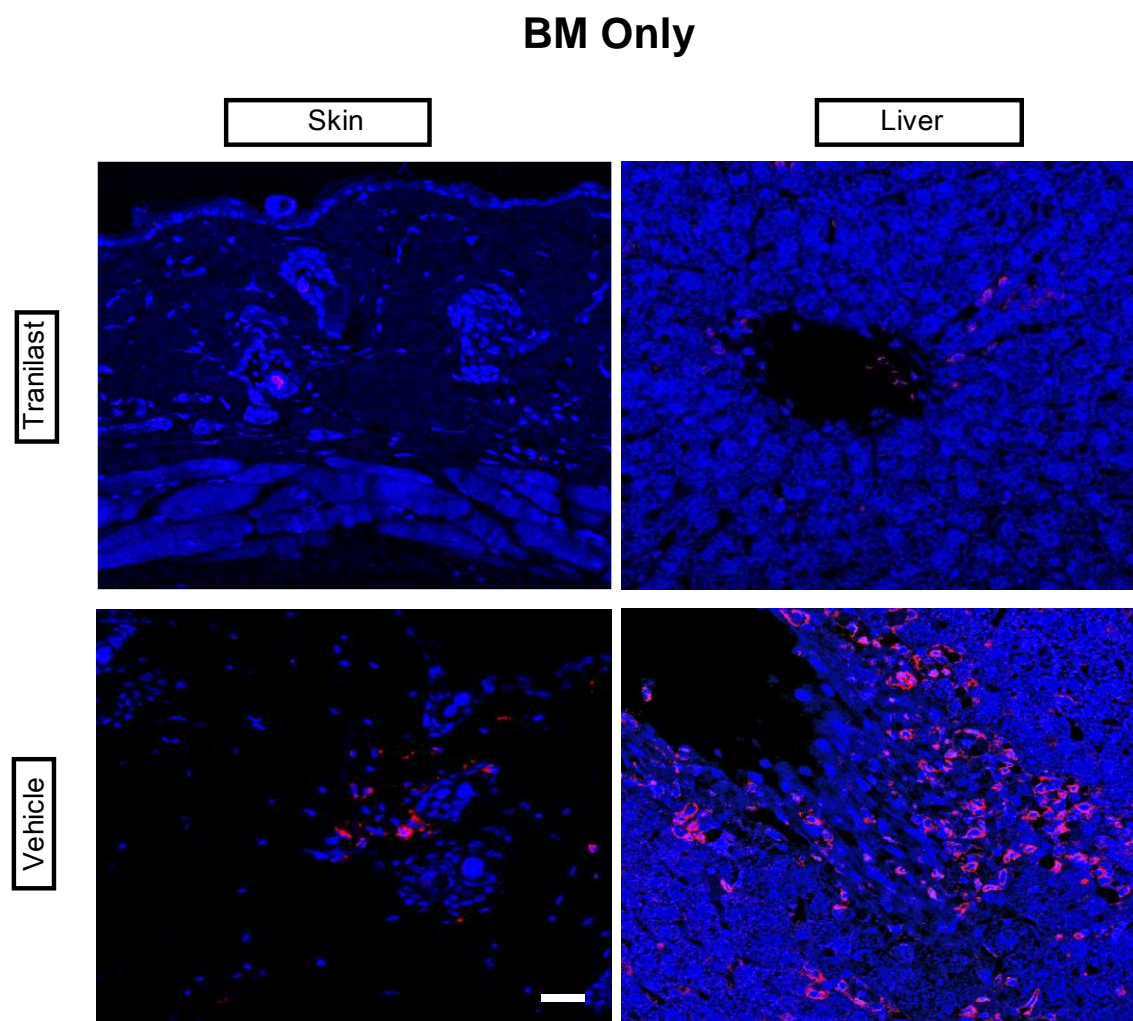

Supplement: S34 Fig — CD45 and cell nuclei are stained red and blue, respectively. The images were taken at 200x magnification, and the scale bar is 20 μm. (PDF) [file pone.0203742.s035.pdf]

**S35 Fig.**

**BM Only**

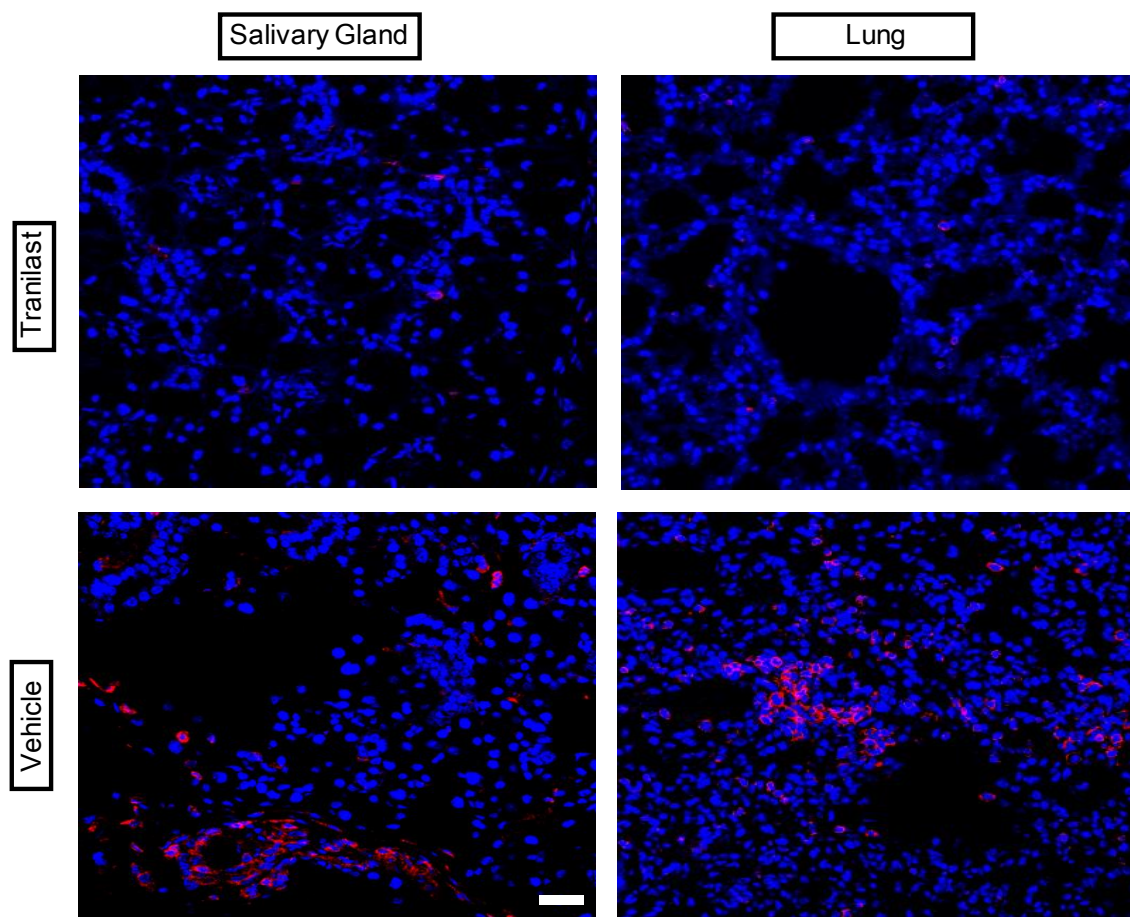

Supplement: S35 Fig — CD45 and cell nuclei are stained red and blue, respectively. The images were taken at 200x magnification, and the scale bar is 20 μm. (PDF) [file pone.0203742.s036.pdf]

**S36 Fig.**

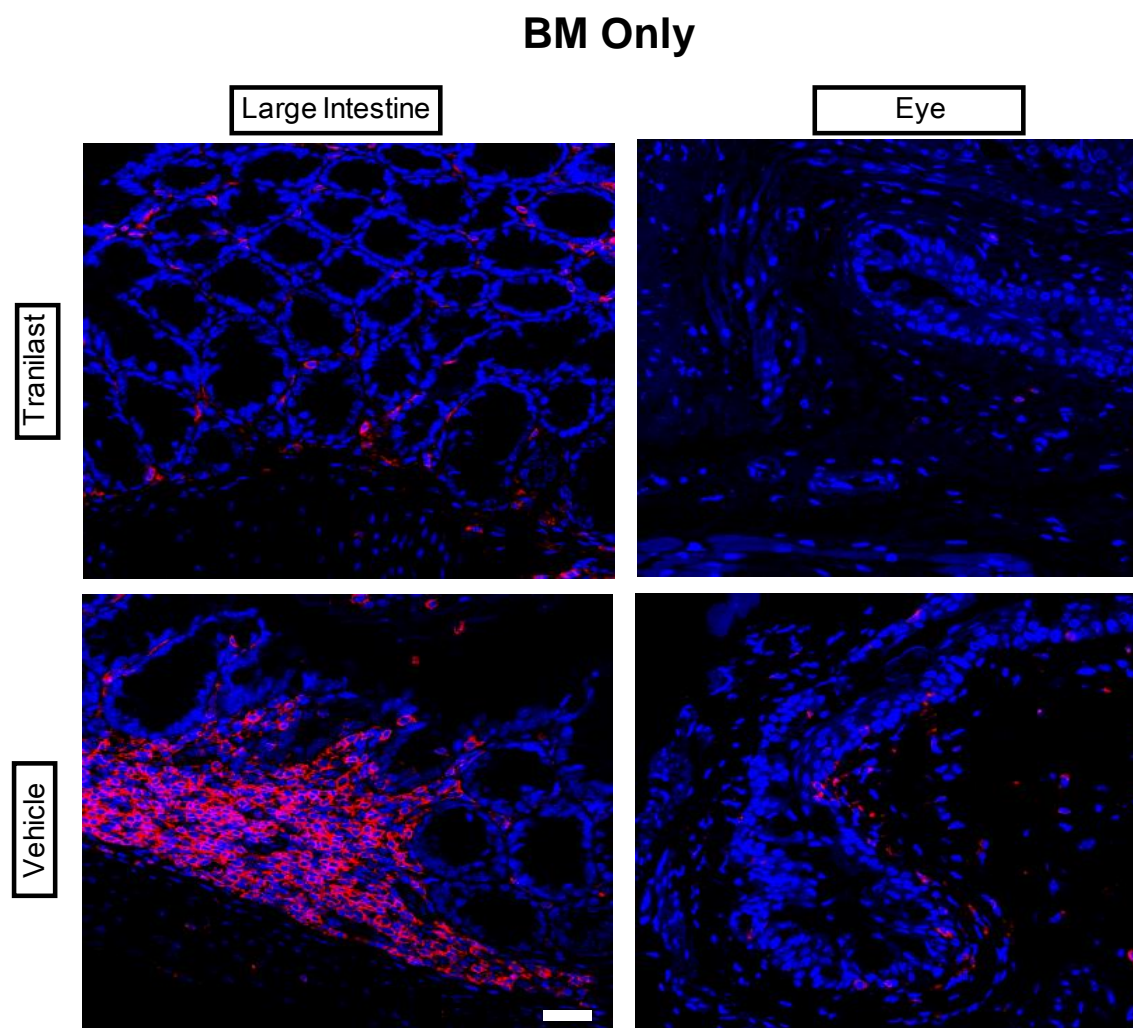

Supplement: S36 Fig — CD45 and cell nuclei are stained red and blue, respectively. The images were taken at 200x magnification, and the scale bar is 20 μm. (PDF) [file pone.0203742.s037.pdf]

**S37 Fig.**

**BM Only**

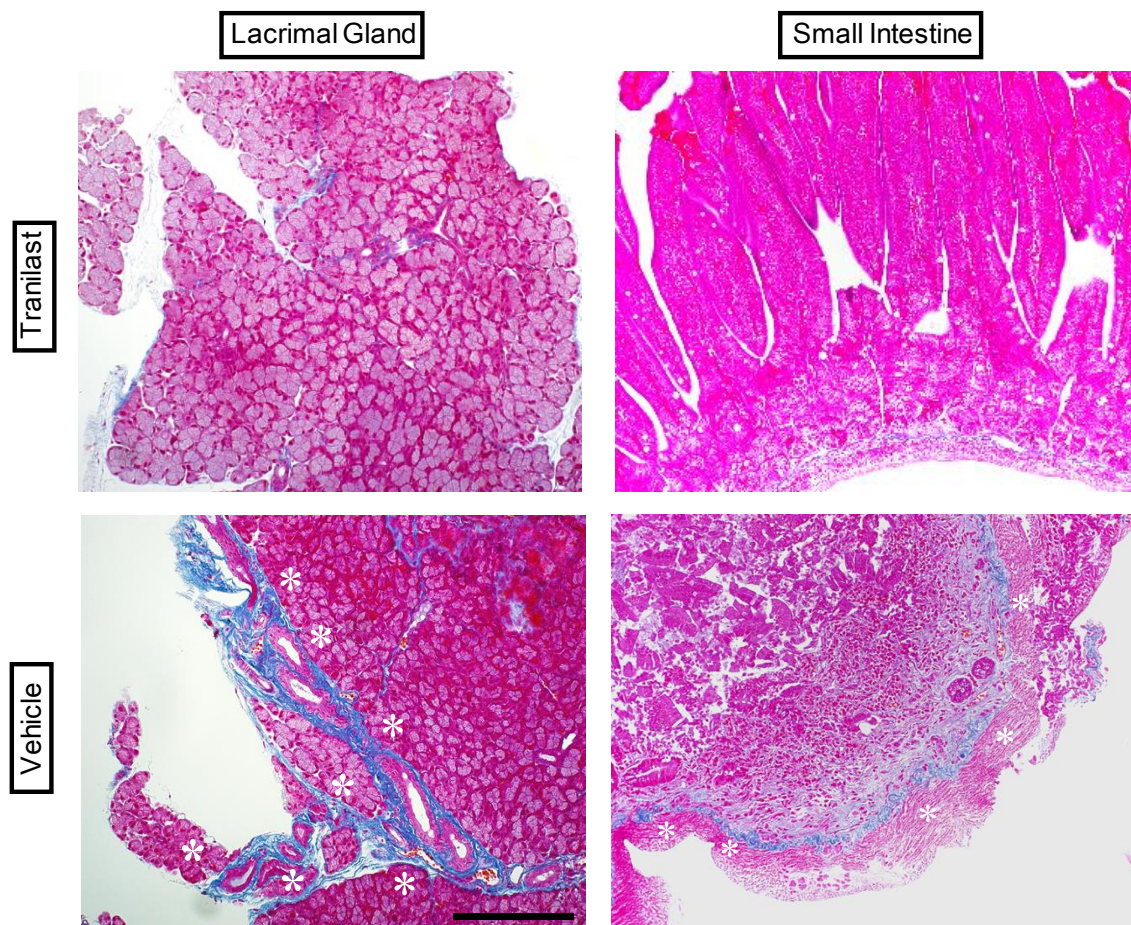

Supplement: S37 Fig — The pictures were taken at 200x magnification, and the scale bar is 200 μm. Excessively fibrotic areas are shown with white asterisks. (PDF) [file pone.0203742.s038.pdf]

**S38 Fig.**

**BM Only**

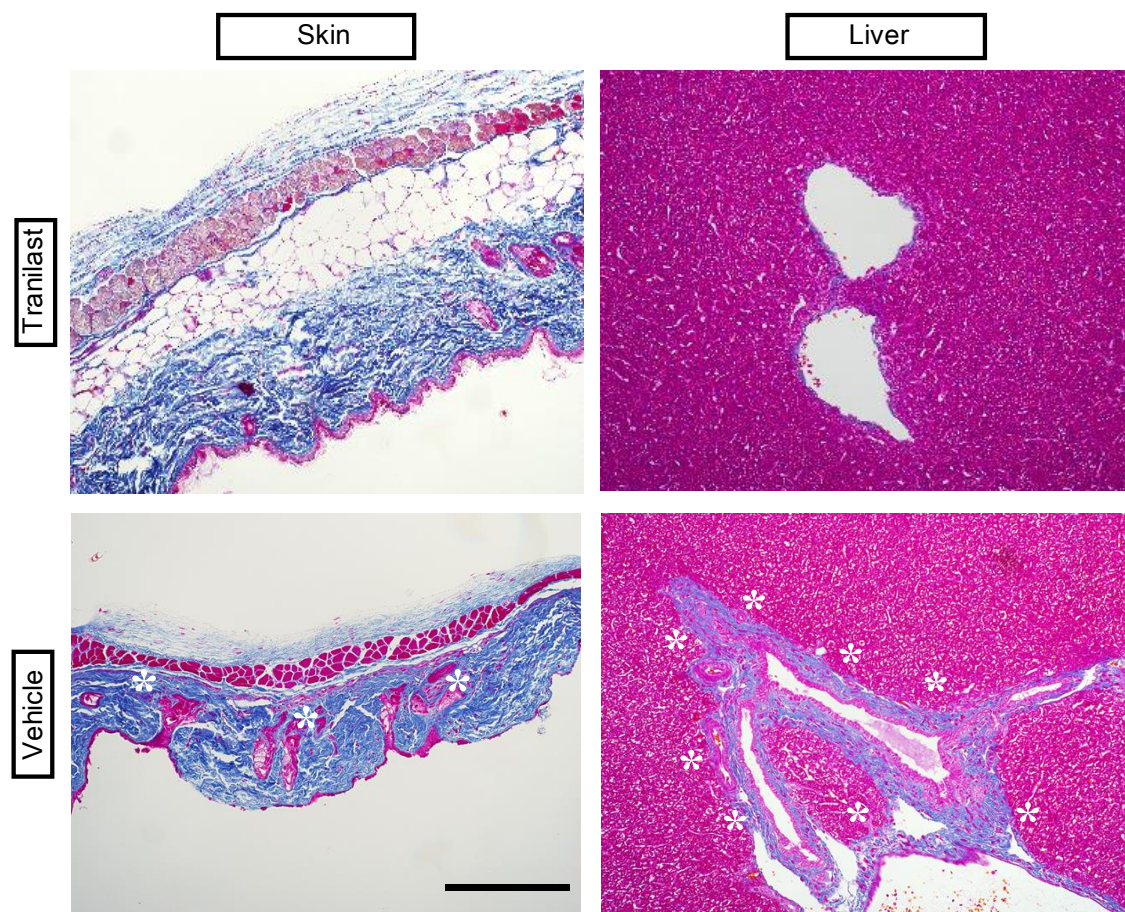

Supplement: S38 Fig — The pictures were taken at 200x magnification, and the scale bar is 200 μm. Excessively fibrotic areas are shown with white asterisks. (PDF) [file pone.0203742.s039.pdf]

**S39 Fig.**

**BM Only**

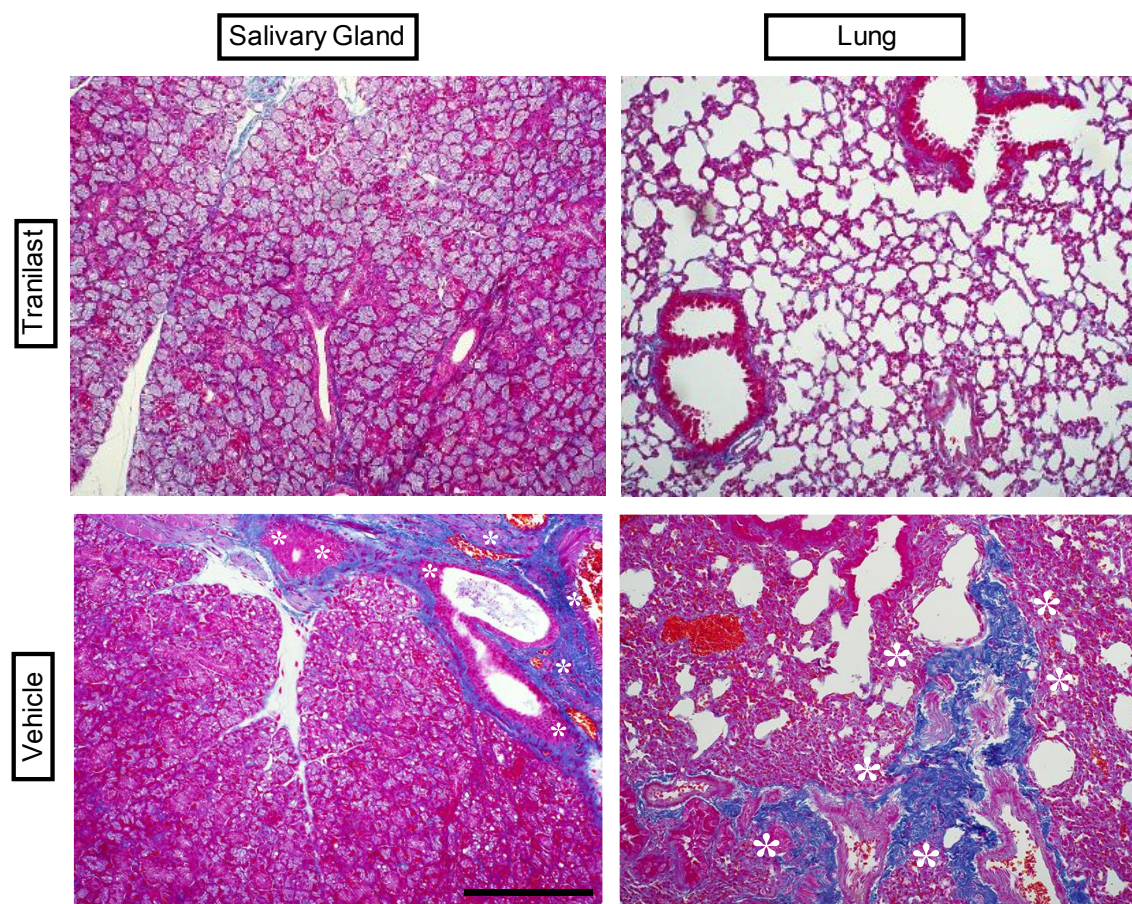

Supplement: S39 Fig — The pictures were taken at 200x magnification, and the scale bar is 200 μm. Excessively fibrotic areas are shown with white asterisks. (PDF) [file pone.0203742.s040.pdf]

**S40 Fig.**

**BM Only**

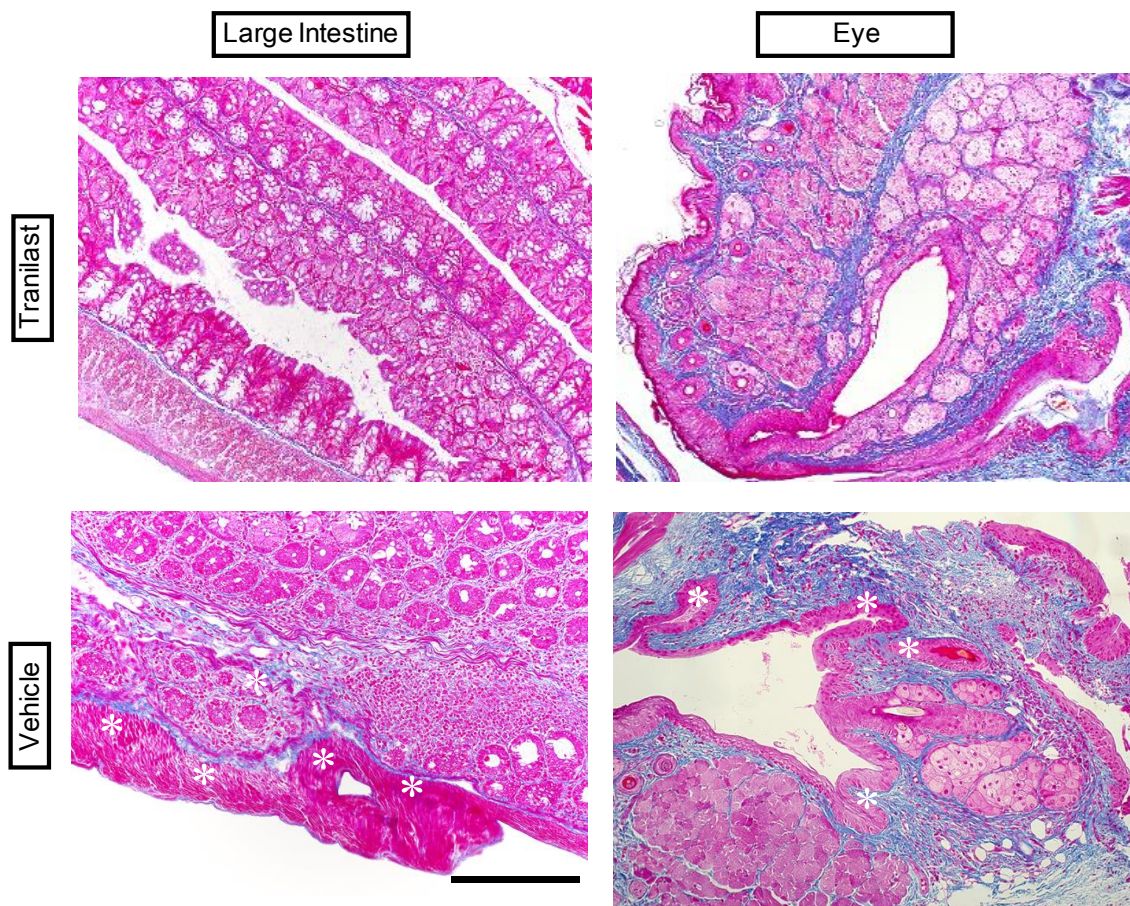

Supplement: S40 Fig — The pictures were taken at 200x magnification, and the scale bar is 200 μm. Excessively fibrotic areas are shown with white asterisks. (PDF) [file pone.0203742.s041.pdf]

**S41 Fig.**

**BM + SC**

TL-treated LG

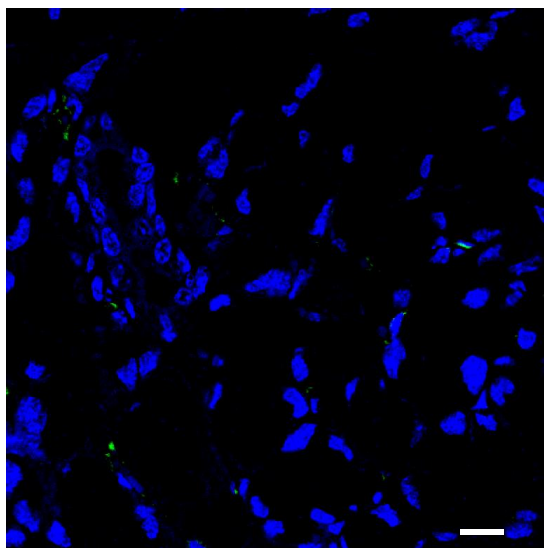

Vehicle-treated LG

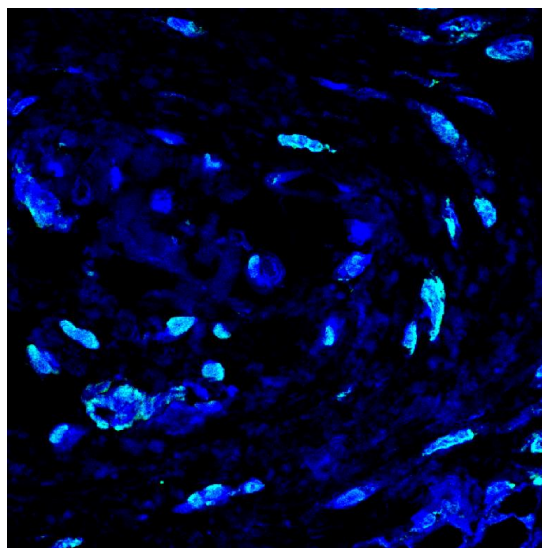

Supplement: S41 Fig — HSP47 and cell nuclei are stained green and blue, respectively. The images were taken at 400x magnification, and the scale bar is 10 μm. (PDF) [file pone.0203742.s042.pdf]

**S42 Fig.**

**BM + SC**

TL-treated LG

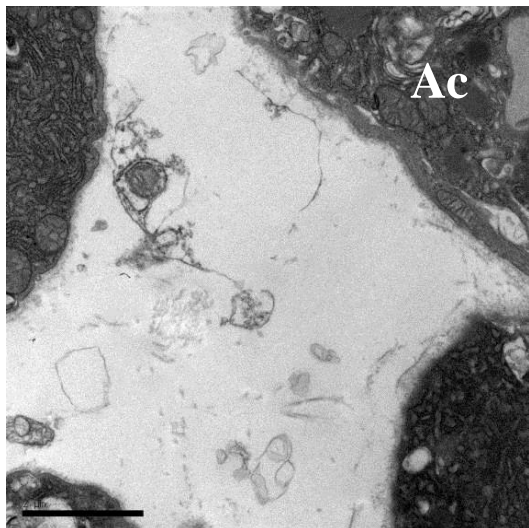

Vehicle-treated LG

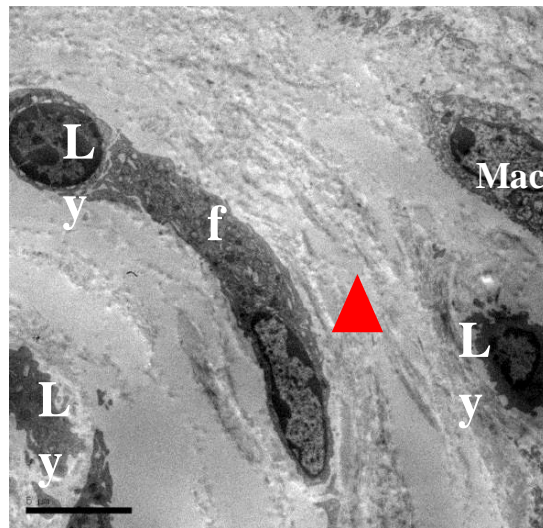

Supplement: S42 Fig — The pictures were taken at 5000x magnification and at 5000x magnification, respectively. The scale bar is 5 μm. Ly; lymphocyte, f, fibroblast, Mac; macrophage, Ac; Acinus. In the image of the vehicle-medicated lacrimal gland, an aberrant collagen bundle is shown with a red triangle. (PDF) [file pone.0203742.s043.pdf]

**S43 Fig.**

**BM + SC**

TL-treated LG

Vehicle-treated LG

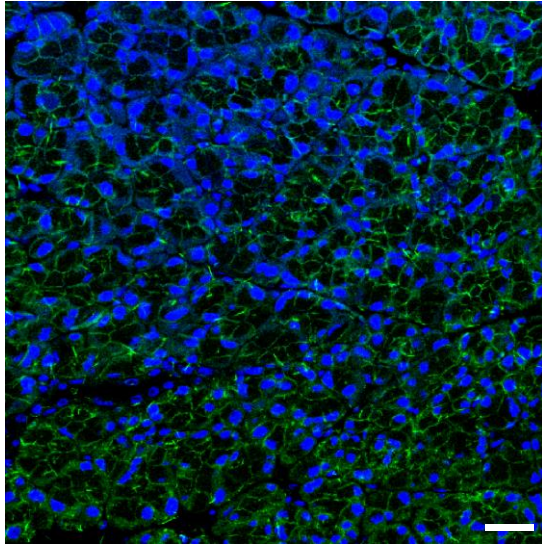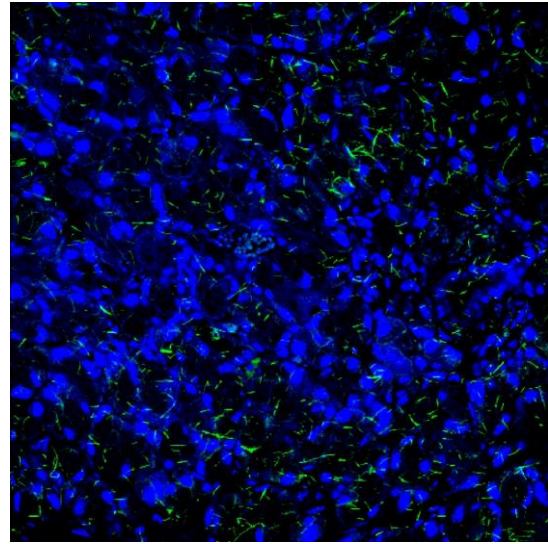

Supplement: S43 Fig — E-cadherin and cell nuclei are stained green and blue, respectively. The pictures were photographed at 200x magnification, and the scale bar is 20 μm. (PDF) [file pone.0203742.s044.pdf]
